# Discovery of novel thyrointegrin αvβ3 antagonist fb-PMT (NP751) in the management of human glioblastoma multiforme

**DOI:** 10.1093/noajnl/vdac180

**Published:** 2022-12-08

**Authors:** Kavitha Godugu, Bruce A Hay, Gennadi V Glinsky, Shaker A Mousa

**Affiliations:** The Pharmaceutical Research Institute, Albany College of Pharmacy and Health Sciences, Rensselaer & NanoPharmaceuticals LLC, Rensselaer, New York, USA; The Pharmaceutical Research Institute, Albany College of Pharmacy and Health Sciences, Rensselaer & NanoPharmaceuticals LLC, Rensselaer, New York, USA; Institute of Engineering in Medicine, University of California, San Diego, La Jolla, California, USA; The Pharmaceutical Research Institute, Albany College of Pharmacy and Health Sciences, Rensselaer & NanoPharmaceuticals LLC, Rensselaer, New York, USA

**Keywords:** antiangiogenesis, blood-brain barrier, gene expression profiling, glioblastoma, thyrointegrin αvβ3

## Abstract

**Background:**

Thyrointegrin αvβ3 receptors are unique molecular cancer therapeutic targets because of their overexpression on cancer and rapidly dividing blood vessel cells compared and quiescent on normal cells. A macromolecule, *T*ri*A*zole *T*etraiodothyroacetic acid (TAT) conjugated to polyethylene glycol with a lipophilic 4-fluorobenyl group (fb-PMT and NP751), interacts with high affinity (0.21 nM) and specificity with the thyrointegrin αvβ3 receptors on the cell surface without nuclear translocation in contrast to the non-polymer conjugated TAT.

**Methods:**

The following in vitro assays were carried out to evaluate NP751 including binding affinity to different integrins, *transthyretin* (TTR)-binding affinity, glioblastoma multiforme (GBM) cell adhesion, proliferation assays, nuclear translocations, chorioallantoic membrane model of angiogenesis, and microarray for molecular mechanisms. Additionally, in vivo studies were carried out to evaluate the anticancer efficacy of NP751, its biodistribution, and brain GBM tumor versus plasma levels kinetics.

**Results:**

NP751 demonstrated a broad spectrum of antiangiogenesis and anticancer efficacy in experimental models of angiogenesis and xenografts of human GBM cells. Tumor growth and cancer cells’ viability were markedly decreased (by > 90%; *P* < .001) in fb-PMT-treated U87-luc or 3 different primary human GBM xenograft-bearing mice based on tumor in vivo imaging system (IVIS) imaging and histopathological examination, without relapse upon treatment discontinuation. Additionally, it effectively transports across the blood-brain barrier via its high-affinity binding to plasma *TTR* with high retention in brain tumors. NP751-induced effects on gene expression support the model of molecular interference at multiple key pathways essential for GBM tumor progression and vascularization.

**Conclusions:**

fb-PMT is a potent thyrointegrin αvβ3 antagonist with potential impact on GBM tumor progression.

## Graphical Abstract

**Graphical Abstract:**
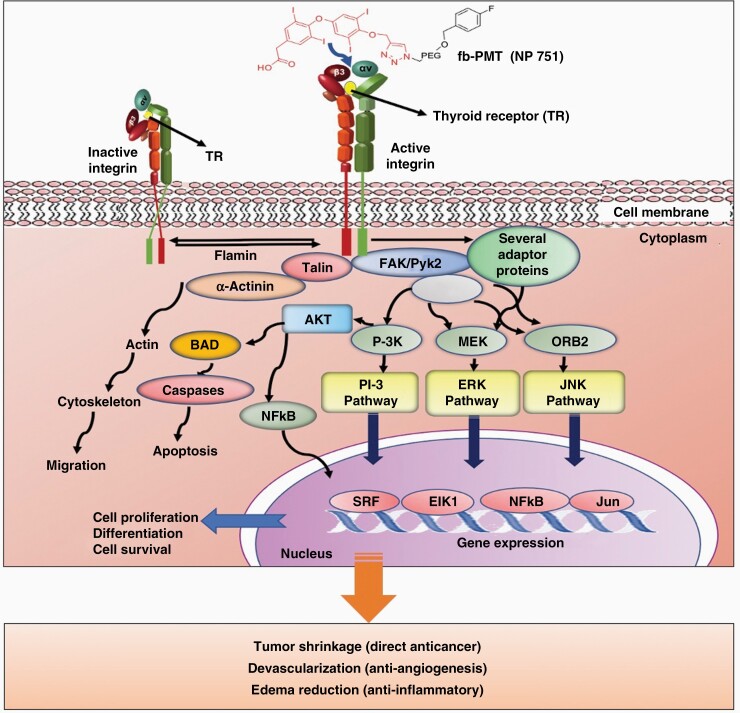

**Key Points**

- NP751 is a high affinity and specificity thyrointegrin αvβ3 antagonist.
- It effectively transports across blood-brain barrier and promotes GBM tumor necrosis, and devascularization without relapse.
- NP751 is a lead clinical candidate for the management of human GBM.

**Importance of the Study**

Relapsed glioblastoma multiforme (GBM) at initial diagnosis, has a poor prognosis and will express radio- and chemoresistance. Thyrointegrin αvβ3 receptors are unique molecular cancer therapeutic targets because of their overexpression of cancer and rapidly dividing blood vessel cells compared. In the present study, we focused on thyrointegrin αvβ3 antagonist, fb-PMT (NP751). Its high affinity and specificity interactions with the thyrointegrin αvβ3 receptors on the cell surface without nuclear translocation. NP751 showed antiangiogenesis and anticancer efficacy in experimental models. Tumor growth and cancer cells’ viability were markedly decreased (by > 90%) in NP-751-treated U87-luc or primary human GBM xenograft-bearing mice without relapse upon treatment discontinuation. NP751 is transported across the blood-brain barrier via its high-affinity binding to plasma *transthyretin*. Furthermore, overall gene expression profiling experiments indicated NP751-induced effects on gene expression support the model of molecular interference at multiple key pathways essential for GBM tumor progression, inflammation, and angiogenesis.

Glioblastoma multiforme (GBM) is a highly aggressive and fatal form of malignant brain cancer (classified as a grade IV glioma, according to World Health Organization classification system for tumors).^1–3^ Due to the severity of the disease, patients survive an average of 14 months and most do not survive beyond 2 years.^4,5^ Median time to recurrence with standard therapy (surgery, radiation, and conventional chemotherapy, eg, with temozolomide, TMZ) is 6.9 months. The tumors manifest inherent or acquired resistance to TMZ^6^ and other chemotherapeutic agents and are radioresistant.^7^ The cancer is highly vascular, but clinical antiangiogenic therapeutic intervention, such as with bevacizumab, has been disappointing.^8^ Incomplete understanding of the molecular basis of this complex disease and low effectiveness of current conventional therapies contribute to the poor prognosis of GBM.^4,5^

Overall, primary GBM are associated with shorter survival time. However, there is a need for the prediction of survival stratification in GBM patients with standard treatment to facilitate clinical decision-making.^9–11^ Relapsed GBM, regardless of the status at initial diagnosis, has a poor prognosis along with radio- and chemoresistance.^5,12^ GBM tumors can be found in all regions of the brain but are most commonly found in the central hemispheres. GBM is most commonly detected by brain imaging, including magnetic resonance imaging, magnetic resonance spectroscopy, positron emission tomography and computed tomography scan, and confirmation is established on biopsy.^11,13^

A number of novel therapeutic approaches to GBM are under investigation.^14–18^ However, resistance mechanisms are expressed by GBMs and readily contravene therapeutic interventions. The tumors rapidly repair DNA breaks induced by alkylating agents, such as TMZ, and by radiation, thus expressing chemo- and radioresistance.^12^ The tumors export chemotherapeutic agents such as TMZ^6^ and doxorubicin by several mechanisms, including plasma membrane P-glycoprotein (MDR1)^19^; thus, limiting the drug effectiveness. GBMs exhibit activation of multiple antiapoptotic pathways and often express constitutively active (mutated) epidermal growth factor receptor (EGFR)^1,20^ and hypoxia-initiated defensive responses^21^ that limit effectiveness of therapeutic interventions. Thus, new approaches to GBM that are directed at single individual pathway(s) have limited likelihood of success.

The αvβ3-dependent anticancer properties of tetrac are related to the regulation of cancer biology-relevant gene expression implicated in multiple cancer growth and survival-promoting pathways: Genes whose products control apoptosis, cell cycle and proliferation, survival pathway components, and angiogenesis.^22–24^ Notably, antiangiogenic agents such as bevacizumab that are targeted to a single vascular growth factor, such as VEGF, have improved 6-month progression-free survival in GBM, but not overall survival.^25^

Recently, our laboratory has described a high affinity “thyrointegrin” αvβ3 antagonist, P-bi-TAT, a tetraiodothyroacetic acid conjugated to polydisperse polyethylene glycol (PEG, MW 4000). It showed high affinity to the αvβ3 receptor (0.14 nM) and favorable anticancer properties in human GBM xenografts.^26^ However, the large polydisperse PEG portion of the molecule resulted in bioanalytical issues in quality control and difficulty in synthesis and scalability. Here, we focused on a novel thyrointegrin αvβ3 antagonist, fb-PMT (NP751) synthesized by chemically modifying tetrac by adding a triazole linker, which in turn is covalently bonded to a PEG chain with exactly 36 repeating units. The other end of the PEG chain is capped with a 4-fluorobenyl group. The resultant fb-PMT molecule constitutes a novel single chemical entity with good water solubility, stability, and scalability. Previously we reported the fb-PMT synthesis, documented fb-PMT bioavailability in vivo to target human GBM tumors implanted in the brain of mice, and demonstrated its potent anti-proliferative effect on glioblastoma cells.^27^ In the current study, we evaluated the anticancer actions and pharmacology of fb-PMT in human GBM tumor xenografts derived from cell lines and patient-derived xenografts (PDX) using 3 different human primary GBM cells. Furthermore, the molecular mechanisms of anticancer activities of fb-PMT were explored by employing genome-wide expression profiling experiments. Finally, the biologically relevant impacts of fb-PMT-mediated molecular interference mechanisms were investigated in an experimental model of growth factor-induced angiogenesis. Experimental observations demonstrate potent broad-spectrum anti-angiogenesis, anti-inflammatory, and anti-cancer activities of the fb-PMT, and taken together with its favorable safety, toxicity, biodistribution, and bioavailability profiles, make this novel molecule a promising anti-cancer drug candidate for further in-depth exploration in GBM clinical trial.

## Materials and Methods

### Chemicals and Reagents

fb-PMT (NP751) was synthesized according to our previously described method.^27^ The human glioblastoma U87-luc cells were purchased from ATCC and primary human glioblastoma cells (GBM 101813, GBM 021913, and GBM 052814) were a generous gift from the University at Pittsburgh Medical Center, Department of Neurosurgery (Pittsburgh, PA). Dulbecco’s Modified Eagle Medium (DMEM), penicillin/streptomycin, and trypsin/EDTA, fetal bovine serum, bovine serum albumin were purchased from Sigma-Aldrich. Integrins αvβ1, αvβ3, αvβ5, αvβ6, IIbβ3, and α5β1 were obtained from ACRO Biosystems. HRP-Anti His Tag Monoclonal Mouse antibody and biotinylated human integrins were purchased from R&D systems, streptavidin HRP conjugate was from ThermoFisher Scientific, fibrinogen was from Millipore Sigma, 3,3′,5,5′-tetramethylbenzidine (TMB), and TMB-stop solution were from ABCAM Inc.

### Binding Affinity Measurements of fb-PMT to a Panel of Molecularly Distinct Integrins

The binding affinity of fb-PMT to purified integrins, including the αvβ3 and 5 other distinct integrins (αvβ1, αvβ5, αvβ6, IIbβ3, α5β1) was measured using a previously described method^28–31^ (see Supplementary Material).

### Transthyretin Binding Assay

Transthyretin (TTR)-binding affinity of fb-PMT was tested in a competitive binding assay with a fluorescent conjugate of T4 and fluorescein 5-isothiocyanate (FITC) as previously described^32–34^ (see Supplementary Material).

### Chorioallantoic Membrane Model of Neovascularization Method

#### ***Animals***.—

Immunodeficient female NCr nude homozygous mice aged 5–6 weeks weighing 20–25 g were purchased from Taconic Biosciences, Inc. (see Supplementary Material). All animal studies were conducted at the animal facility of the Veteran Affairs Medical Center in accordance with approved institutional guidelines for humane animal treatment and according to the current guidelines. Mice were maintained under specific pathogen-free conditions and housed under controlled conditions of temperature (20–24°C) and humidity (60%–70 %) and 12 h light/dark cycle with ad libitum access to water and food. Mice were allowed to acclimatize for 5 days before the study.

#### *Glioblastoma xenografts*.—

For the subcutaneous (s.c.) GBM tumor model, the study was conducted in accordance with experimental protocols diagrammed in Supplementary Figure S1. U87-luc cells or primary GBM cells were harvested, suspended in 100 μL of DMEM with 50 % Matrigel, and 2 × 10^6^ cells were implanted s.c. dorsally in each flank to achieve 2 independent tumors per animal. Immediately prior to initiation of treatments, animals were randomized into treatment groups (5 animals/group) by tumor volume measurements with Vernier calipers. Treatments were begun after the detection of palpable tumor mass (4–5 days postimplantation). When compounds had different percentages of the active tetrac portion (s) of the molecule relative to the full molecule, we dosed the compounds at equivalent moles/kg triazole tetrac (TAT) levels rather than mg/kg of the intact compound. The treatments were control Phosphate Buffered Saline (PBS), 1.0 mg/kg, 3 mg/kg, 6 mg/kg, and 10 mg/kg, the agents were administered daily, s.c. for 21 days (ON Treatment) and in another set of animals, compounds were administrated daily for 21 days followed by 21 days discontinuation (ON Treatment + OFF Treatment). Animals were then humanely sacrificed, and tumors were harvested. Tumor weights and cell viability (bioluminescent signal intensity) were measured.

For Histopathology, the tumors were fixed in 10% formalin, processed and stained with Harris’s hematoxylin and eosin (H & E), and examined under a light microscope.

Uptake of dye-labeled compound in the brain, sample preparation of plasma and brain homogenate with liquid-liquid extraction and Liquid Chromatography/Mass Spectroscopy/ Mass Spectroscopy (LC-MS/MS) instrumentation method (see Supplementary Material).

RNA isolation from GBM cells and gene expression profiling experiments (see Supplementary Material).

### Statistical Analysis

An overall comparison of the means for all groups was carried out using a one-way ANOVA. Tukey confidence intervals were used to test for differences in means for each experimental group versus the control group. Results are presented as means ± SD. A value of *P* < .05 indicated a statistically significant difference. Statistical analysis was performed with GraphPad Prism 7 software. Data are presented as mean ± SD. For comparison between 2 or more sets of data, ANOVA was used. *P *<* .05, **P < .01, and ***P < .001 were considered statistically significant.

## Results

### Binding Affinity of fb-PMT to Distinct Molecular Subtypes of Integrins

**Figure 1.**
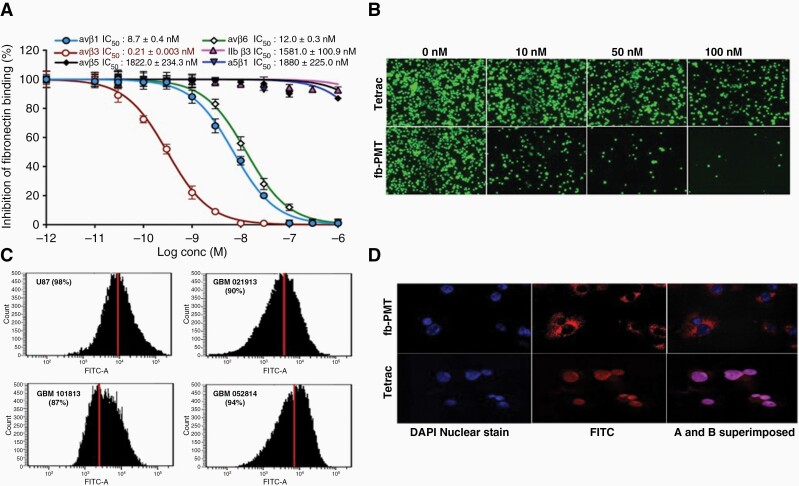
Binding affinity of fb-PMT to purified integrin receptors and αvβ3 overexpressing cancer cells and expression of αvβ3 protein in glioblastoma cancer cells and localization of fb-PMT.

To evaluate the specificity and selectivity of fb-PMT binding to integrins, we investigated fb-PMT binding to the 6 distinct molecular subtypes of integrins (αvβ1, αvβ3, αvβ5, αvβ6, IIbβ3, and α5β1) in an integrin-ligand binding assay. fb-PMT showed IC_50_ values 8.7 nM, 0.21 nM, 1822 nM, 12 nM, 158 nM, and 1880 nM for integrins αvβ1, αvβ3, αvβ5, αvβ6, IIbβ3, α5β1, respectively. The lowest IC_50_ value for fb-PMT, 0.21 nM, was observed in experiments with αvβ3 (Figure 1A). Therefore, fb-PMT appears to manifest a relatively high binding affinity to the αvβ3 integrin compared to the other molecular types of integrins. The binding affinity toward αvβ3 integrin was 41-fold higher compared to the second most active target, αvβ1 integrin, and as high as 8952-fold compared to the least active target, α5β1 integrin. Furthermore, a cell-based adhesion assay showed that with increasing concentrations of fb-PMT the adhesion of αvβ3-expressing U87 cells to the fibrinogen-coated plates decreased in a dose-dependent manner (Figure 1B).

(A) Binding affinity of fb-PMT to integrins αvβ1, αvβ3, αvβ5, αvβ6, IIbβ3, and α5β1 αvβ3. Integrin-coated 96-well plates were incubated with increasing concentrations of fb-PMT in the presence and absence of fibrinogen. Dose response curve (mean IC_50_ ± SD) of 3 independent experiments. fb-PMT has a significantly higher binding affinity to αvβ3 with lower IC_50_ values compared to other integrins. (B) Dose-dependent binding of fb-PMT to αvβ3-over expressing U87 cells by adhesion assay. U87 cells labeled with calcein AM were seeded onto fibrinogen-coated wells in the presence of increasing concentrations of fb-PMT and incubated for 2 h before measuring the fluorescence of the intact cells. Representative fluorescent images at 0, 10, 50, and 100 nM of fb-PMT. (C) Expression of αvβ3 protein in glioblastoma cancer cells was shown by Flow cytometry. (D) Confocal imaging showing the uptake of fb-PMT and Tetrac in U87-luc cells (incubation time = 3 h), 63Å~. Excitation wavelength was 405 nm and emission was detected between 565 and 688 nm. Cells were mounted with Prolong Gold antifade reagent with 4′, 6-diamidino-2-phenylindole, which is DNA nuclear stain (DAPI), and DAPI was used for staining the nucleus. Representative images of cellular distribution of DAPI, FITC fb-PMT, and both superimposed, showing a lack of nuclear translocation for fb-PMT.

### TTR Binding Assay and In vivo Biodistribution and Bioaccumulation of fb-PMT

**Figure 2.**
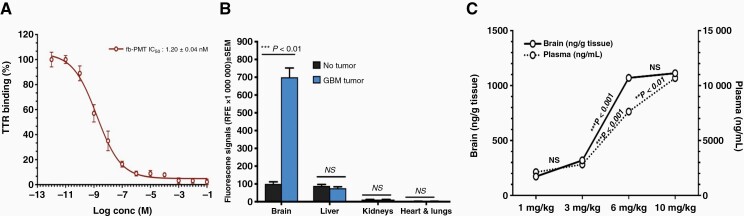
TTR binding assay and bioaccumulation in vivo of fb-PMT in various tissues of control and human GBM-bearing mice.

Brain penetration is critical for GBM therapies, and we postulated that the tetrac moiety in fb-PMT would result in fb-PMT being a substrate of the thyroid hormone transporter TTR, facilitating fb-PMT crossing the blood-brain barrier. To estimate fb-PMT binding affinity in interactions with biologically relevant forms of thyroxine, we utilized the TTR binding assay. TTR is a protein component of blood serum that functions to specifically bind and transport thyroxine in the body. We tested the fb-PMT-TTR-thyroxine (T_4_) interactions using a competitive fluorescence binding assay. FITC-T_4_ and TTR were mixed in a 96-well plate and incubated with different concentrations of fb-PMT, and the intensity of fluorescence was measured at 518 nm. In these experiments, fb-PMT interacts with TTR via its tetrac moiety, binds to TTR, and displaces FITC-thyroxine from TTR. The FITC-T_4_ conjugate shows high fluorescence when its T4 group is bound to TTR, while FITC-T_4_ not bound to TTR does not fluoresce due to the intramolecular quenching of the fluorescein group by the free T4 group. Therefore, the competitive fb-PMT binding to TTR was estimated by measuring a decrease in bound FITC-T4 quantified as a decrease in fluorescence, The IC_50_ value of fb-PMT binding to TTR in this assay was determined to be 1.20 nM (Figure 2A).

(**A**) TTR and FITC-T_4_ were mixed and incubated with different concentrations of fb-PMT. Competitive fluorescence binding curves (mean IC_50_ ± SD) of 3 independent experiments. (B). Bioaccumulation in vivo of fb-PMT in various tissues of control and GBM-bearing mice. (C) Plasma and brain pharmacokinetics of fb-PMT in nude mice with orthotopic glioblastoma multiforme (GBM) tumors, 0–24 h.^27^ Values are presented as mean ± SD. ***P < .001, **P < .01, *P < .05, compared to control. NS, not significant.

Bioaccumulation (Figure 2B) and biodistribution (Figure 2C) of fb-PMT in orthotopic GBM tumors and various tissues were analyzed in athymic female mice. fb-PMT at 1, 3, 6, and 10 mg/kg was administrated subcutaneously, and over a 24 h period plasma and brain samples were collected and fb-PMT concentration was determined using LC-MS/MS (Figure 2C). fb-PMT showed a dose-dependent relationship with plasma Area Under the Curve (AUC), with 0–24 h AUCs of 2118, 2816, 7631, and 10 677 ng/mL*h at 1, 3, 6, and 10 mg/kg, respectively. There were no significant differences in AUC between the 1 and 3 mg/kg doses, but a statistically significant increase in AUC going from the 3 to 6 to 10 mg/kg doses was observed. Furthermore, a dose-dependent increase in delivery and brain tumor uptake and retention was demonstrated with a maximal profile at the 6 and 10 mg/kg doses.

Figure 2B demonstrates 7 folds greater differential brain uptake kinetic for fluorescence labeled fb-PMT in orthotopically implanted GBM tumors versus no brain GBM implanted one as monitored by IVIS imaging. Furthermore, Figure 2C demonstrates a dose-response (1, 3, 6, and 10 mg/kg after single s.c. dose), brain uptake kinetic of fb-PMT in orthotopically implanted GBM tumors versuss plasma kinetics. Data demonstrated maximal brain GBM tumor uptake at 6 mg/kg with no further increase after 10 mg/kg. In contrast to linear PK in the blood (Figure 2C).

### Antiangiogenesis Efficacy of fb-PMT Versus Bevacizumab (Avastin) in the Chorioallantoic Membrane Model of Neovascularization

**Figure 3.**
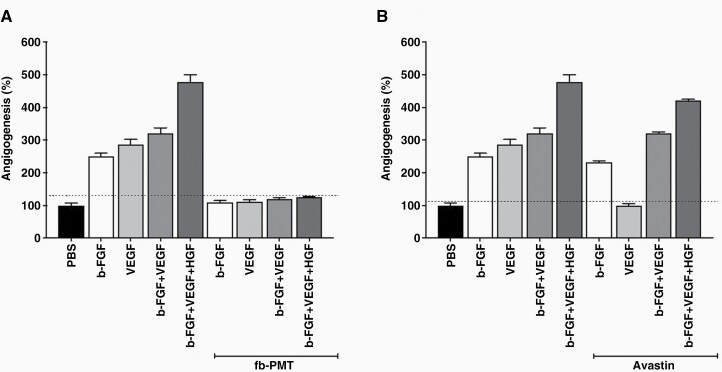
Effect of fb-PMT and Avastin on growth factor-induced angiogenesis in chick chorioallantoic membrane (CAM).

The effect of fb-PMT on micro-vessel formation was studied in the Chorioallantoic Membrane Model (CAM) model. To compare the antiangiogenic efficacy of the fb-PMT, a CAM assay was used. As shown in Figure 3, growth factors (Basic-Fibroblast Growth factor [b-FGF] or VEGF or Hepatocyte Growth Factor [HGF]) were first used to stimulate angiogenesis, then compounds were administrated. The growth factors (b-FGF or VEGF or HGF) at 10 ng/CAM induced angiogenesis 4-fold more than the control (PBS). B-FGF, VEGF, and HGF-mediated angiogenesis were significantly eliminated by fb-PMT at a concentration of 1 μg/CAM (Figure 3A) However, while Avastin (bevacizumab) the known inhibitor of VEGF-induced angiogenesis, inhibited VEGF induced angiogenesis, it had no effect on bFGF and HGF induced angiogenesis, or their combinations (Figure 3B). The CAM study results provided evidence that the multiple modes of angiogenesis inhibition seen with fb-PMT might play an important role in its anticancer activity.

Inhibition of pro-angiogenesis factors (b-FGF, VEGF, and HGF) (10 ng/CAM) induced angiogenesis (A) fb-PMT (1 µg/CAM); (B) Avastin (10 µg/CAM). b-FGF, fibroblast growth factor; VEGF, vascular endothelial illustrated the decrease of compounds. Values are presented as mean ± SD. ***P *<* .001, compared to control.

### Antitumor Efficacy

**Figure 4.**
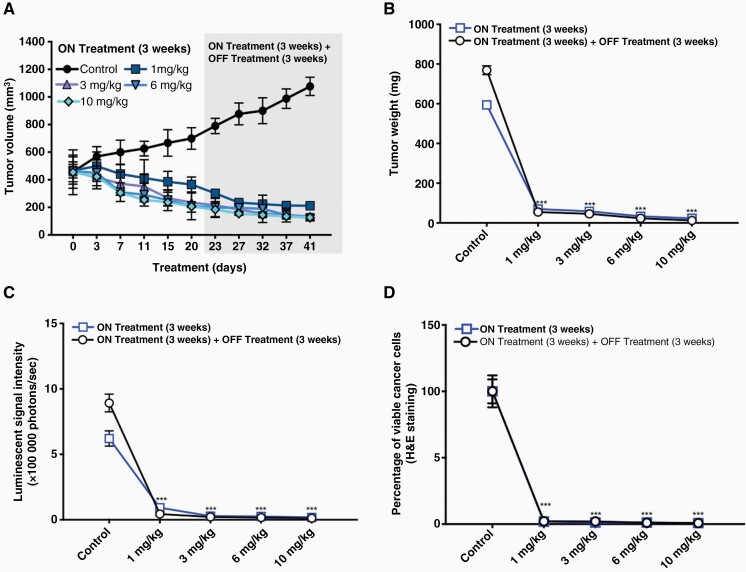
Antitumor efficacy of fb-PMT in U87-luc implanted xenografts. U87-luc glioblastoma cells implanted mice were treated daily with fb-PMT (ON Treatment) for 21 days and in the second group the xenografts were observed for an additional 21 days with no further treatment (ON Treatment + OFF Treatment). (A) Tumor volume; (B) Tumor weight; (C) Luminescent signals; (D) Viable cancer cells (H & E staining). Values are presented as mean ± SD. ***P *<* .001, **P *<* .01, *P *<* .05, compared to control.

With the intent to study the in vivo antitumor efficacies of fb-PMT on tumor growth, U87-luc glioblastoma cells were implanted in each flank of each animal, and then the mice were treated daily for 21 days with fb-PMT. Tumor volumes were decreased in dose-dependent manner when treated with fb-PMT compared to controls (Figure 4).  All doses (1 mg/kg, 3 mg/kg, 6 mg/kg, and 10 mg/kg)  reduced tumor weight by > 90% (ON Treatment, ***P < .001) compared to control after daily treatment for 21 days. In the second group of fb-PMT-treated mice, the xenografts were observed for an additional 21 days with no further treatment (ON Treatment + OFF Treatment). There was no regrowth of tumors in these groups of animals and the absence of cell viability persisted (Figure 4A and B).

The in vivo luminescent signals of viable cancer cells were quantified (photons/second) for the different groups using Xenogen-IVIS Spectrum. A statistically significant (*P < *.001) decrease of viable U87-luc cells was observed in groups treated with fb-PMT compared to control. No significant difference was observed between the treated groups (Figure 4C).

Furthermore, the histological sections obtained from the U87 xenografts were used to evaluate the antagonist treatments on cell proliferation. A large necrotic area was observed in the tumor masses from all doses of the treatment groups. However, there was no significant difference between the 3 antagonist treatments (Figure 4D).

### PDX Model

**Figure 5.**
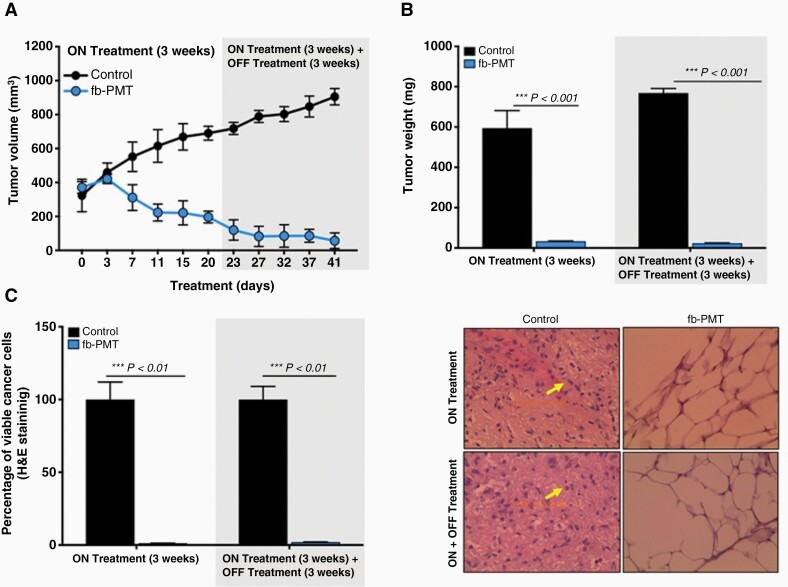
Antitumor efficacy of fb-PMT in GBM 101813. Glioblastoma primary cells GBM 101 813 cells implanted mice were treated daily with fb-PMT at 6 mg/kg body weight (ON Treatment) for 21 days and in the second group the xenografts were observed for an additional 21 days with no further treatment (ON Treatment + OFF Treatment). (A) Tumor volume; (B) Tumor weight; (C) Viable cancer cells (H & E staining); (D) Representative images of H & E staining. Values are presented as mean ± SD. ***P *<* .001, **P *<* .01, *P < .05, compared to control.

Furthermore, the anticancer efficacy of fb-PMT was examined using PDX. GBM 101813, GBM 021913, or GBM 052814 cells were implanted in each flank of the animal, and then the mice were treated daily with fb-PMT at 6 mg/kg for 21 days. Tumor volumes were decreased with fb-PMT (6 mg/kg) for 21 days (ON Treatment) and another arm was treated daily for 21 days followed by discontinuation for 21 days (ON + OFF Treatment) (Figure 5 and Supplementary Figures S1 and S2).

### Overview of Mechanisms of Anticancer Activities of fb-PMT Using Genome-Wide Expression Profiling of Human GBM Cells

We carried out genome-wide expression profiling analyses of U87-luc human GBM cells treated with nontoxic doses of fb-PMT. RNAseq experiments identified 1397 genes, the expression of which was significantly altered by fb-PMT (Supplementary Table S1). There were 633 down-regulated and 764 up-regulated differentially expressed genes (DEGs) identified at the *P* value < .05 adjusted for multiple hypothesis testing (Supplementary Table S1). At the threshold of nominal *P* value < .05, 3673 DEGs were identified, 2085 and 1588 of which were up- and down-regulated, respectively. To understand potential mechanisms of fb-PMT anticancer activities and gain insights into biological and molecular functions of genes expression of which was significantly affected in GBM cells by fb-PMT treatment, Gene Set Enrichment Analyses (GSEA) of 764 up-regulated and 633 down-regulated DEGs were carried out using the Enrichr bioinformatics platform (Methods). These analyses applied to ~ 30 genomics and proteomics databases identified hundreds of significantly enriched records (adjusted *P* value < .05) and associated sub-sets of DEGs significantly affected by fb-PMT treatment of human GBM cells (Supplementary Table S1). Notable examples of significantly affected signal transduction pathways of direct relevance to pathogenesis of GBM include Glioma Stem Cell Program Activation and Secondary Glioblastoma pathways (Elsevier Pathway Collection database) as well as Glioblastoma Signaling Pathway (WikiPathways 2021 Human database). Several signal transduction pathways with established or suspected roles in GBM pathogenesis have been identified by independent GSEA of multiple databases: Notch Signaling Pathway (NCI-Nature 2016; WikiPathways 2021 Human; KEGG 2021 Human database; BioPlanet 2019 database; Reactome 2016 database); Hedgehog Signaling Pathway (NCI-Nature 2016; WikiPathways 2021 Human; KEGG 2021 Human database; BioPlanet 2019 database; Panther 2016 database); NGF Signaling Pathway (Reactome 2016 database; BioPlanet 2019 database); TGF-beta Signaling Pathway (WikiPathways 2021 Human; BioPlanet 2019). Other examples of significantly affected signal transduction pathways of clearly defined potential biological relevance to regulation of angiogenesis as well as tumor cells’ growth and survival include VEGFA-VEGFR2 Signaling Pathway; EGF/EGFR Signaling Pathway; PI3K-AKT Signaling Pathways; PDGF Signaling Pathway; FGFR1/2/3 Signaling Pathway; Integrin’s Signaling in Angiogenesis; HIF2A & HIF1A Signaling Pathway; VEGFR1 & VEGFR2 Signaling Pathway; Pathways Regulating Hippo Signaling; Nuclear Receptors Meta Pathway and Androgen Signaling Pathway; Thyroid Hormone Signaling Pathways and WNT Signaling Pathway; YAP1- and WWTR1 (TAZ) Pathway. A graphical summary of these observations is presented in Supplementary Figure S4 and extended comprehensive documentation of these findings, including corresponding lists of fb-PMT down-regulated DEGs, are reported in Supplementary Table S1.

We observed apparently distinct quantitative patterns of significantly enriched records for signal transduction pathways associated with up-regulated and down-regulated DEGs (Supplementary Tables S1). Compared with up-regulated genes, genes down-regulated following the fb-PMT treatment manifest larger numbers of significant associations among genes whose protein products are engaged in protein-protein interactions (PPI) with transcription factors (reported in the TFs PPI database) and PPI Hub Proteins (proteins known to interact with at least 50 other proteins to form biologically active multi-protein complexes reported in the PPI Hub Proteins database). Gene expression that is up-regulated following fb-PMT treatment manifests a markedly larger number of significant enrichment records among genes whose expression is altered following exposure of cells to various endogenous ligands such as growth factors, cytokines, interleukins, hormones, etc. (reported in the Ligand Perturbations from GEO databases). Noteworthy significantly enriched records of fb-PMT treatment-affected signal transduction pathways triggered by exposures to endogenous ligands include signaling pathways induced by FGF2; Estradiol; TGF-beta; Neuromedin U; Growth Hormone; Thyroid Hormone; IGF-1; and multiple interleukins, including IL-1-beta; IL-2; IL-10; IL-15; IL-33; IL-6; IL-12 (Supplementary Table S1). Notably, GSEA of the DisGeNET database reporting genes implicated in pathogenesis of several thousand human disorders revealed that genes expression of which is down-regulated after fb-PMT treatment manifests significant enrichment among genes identified in 502 various disease records, while fb-PMT up-regulated genes are significantly enriched among genes implicated in 59 disease records (Supplementary Tables S1).

### Integrative and Case-Specific Analyses of Putative Molecular Mechanisms of Anticancer Activity of fb-PMT Inferred From Gene Expression Profiling Experiments

**Figure 6.**
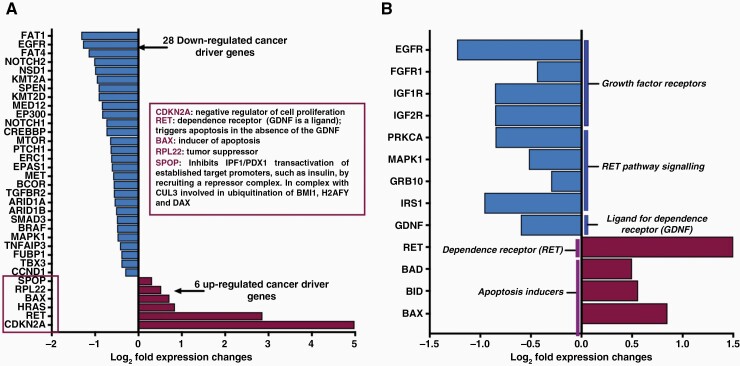
Effects of fb-PMT on the expression of cancer driver genes in human GBM cells. (A) fb-PMT treatment significantly decreases the expression of 26 cancer driver genes (adjusted *P* value < .05). Expression of 6 cancer driver genes was up-regulated, a majority of which displays biological activities consistent with their functions as tumor suppressors. (B) Effects of fb-PMT treatment on expression of selected genes biological functions of which are highly consistent with potent anticancer activity of the fb-PMT. Known RET functions and mechanisms of action support the hypothesis that fb-PMT anticancer activity may be responsible, in part, by effects on RET signaling pathway (see text for details).

We carried out additional analyses to explore the potential molecular mechanisms of the apparently global effects of fb-PMT on signal transduction pathways in GBM cells. To this end, we focused our analytical effort on 2 aspects of fb-PMT actions: (1) integrative analysis of effects on expression of genes whose protein products are engaged in PPI with multiple TFs (TFs PPI) and/or with intracellular proteins known to form the biologically active multiprotein complexes via PPI with at least 50 other proteins (Supplementary Figure S5) and (2) case-specific analysis of effects on expression of cancer driver genes  (Figure 6).

Integrative analyses of fb-PMT down-regulated DEGs whose protein products are engaged in PPI with multiple TFs (TFs PPI) and/or with PPI Hub Proteins reveal the auto-regulatory PPI networks of TFs PPI and PPI Hub proteins that (1) regulate expression of DEGs down-regulated by fb-PMT treatment in human GBM cells, and (2) fb-PMT down-regulate expression of genes encoding the TFs PPI and PPI Hub Proteins constituting members of these PPI networks (Supplementary Figure S5). For example, a set of 62 TFs PPI was identified from 99 TFs interacting via PPI with 633 fb-PMT down-regulated targets (Supplementary Table S1) that regulate expression of fb-PMT target genes (these 62 TFs PPI were designated up-stream regulatory TFs PPI).

Follow-up GSEA of 62 upstream regulatory TFs PPI revealed their interactions in human cells with 196 TFs PPI and 121 PPI Hub Proteins (Supplementary Figure S5A). fb-PMT treatment down-regulated expression of 13/99 TFs PPI (Supplementary Table S1; last column), as well as 7/62 up-stream regulatory TFs PPI; 25/196 TFs PPI; 22/121 PPI Hub Proteins. Similarly, a set of 14 PPI Hub Proteins was selected from 84 PPI Hubs interacting via PPI with 633 fb-PMT down-regulated targets (Supplementary Table S1) that regulate expression of fb-PMT target genes (these 14 PPI Hub Proteins are designated up-stream regulatory PPI Hub Proteins). They interact in human cells via PPI with 184 TFs PPI and 146 PPI Hub Proteins (Supplementary Figure S5B).  fb-PMT treatment down-regulated expression of 21/84 PPI Hub Proteins, as well as 2/14 upstream regulatory PPI Hubs; 23/184 TFs PPI; 29/146 PPI Hub Proteins.

Cancer driver genes, when mutated, promote cancer initiation, development, and progression toward metastatic disease. We observed that the expression of 26 cancer driver genes is downregulated by fb-PMT (Figure 6), representative examples of which include EGFR, NOTCH1/2, MAPK1, CCND1, and BRAF. Among 6 up-regulated cancer driver genes, 4 genes encode proteins known to function as negative regulators of cell proliferation (CDKN2A); inducers of apoptosis (BAX); tumor suppressor (RPL22); inhibitors of transactivation of insulin promoter by recruiting a repressor complex (SPOP). One of fb-PMT up-regulated cancer driver genes (RET) encodes a dependence receptor, which promotes cell survival following interactions with its ligand, GDNF. However, when the GDNF level is diminished, RET triggers apoptosis in target cells. Notably, fb-PMT treatment significantly down-regulates the GDNF expression (Figure 6), suggesting that fb-PMT treatment may activate RET dependence receptor-triggered programmed cell death of GBM cells. Consistent with this hypothesis, we observed that multiple genes engaged in signaling events regulated by RET tyrosine kinase in human cells (ID: e4431190-6195-11e5-8ac5-06603eb7f303) are significantly down-regulated by fb-PMT (*IRS1*; *GRB10*; *MAPK1*; *PRKCA*). We experimentally validated this mode of actions of fb-PMT by demonstrating that anticancer effects of suboptimal 50% inhibitory doses of fb-PMT on human GBM cells could be rescued by GDNF in a dose-dependent manner (Supplementary Figure S3B and E). Conversely, growth-promoting, and pro-survival effects of GDNF on human GBM cells could be markedly inhibited by fb-PMT in a dose-dependent manner (Supplementary Figure S3C and F).

Collectively, these observations support the idea that b-PMT effects on *RET* and *GDNF* expression could be sufficient to induce the RET-dependent programmed cell death of glioblastoma cells. fb-PMT markedly increases *RET* expression and significantly inhibits *GDNF* expression, thus removing the survival ligand for dependence receptor RET and triggering the apoptosis of GBM cells. Known RET functions and mechanisms of action further support the hypothesis that fb-PMT anticancer activity may be responsible, in part, by effects on RET signaling pathway. RET regulates both cell death/survival balance and positional information and modulates cell adhesion via its cleavage by caspase in sympathetic neurons as well as mediates cell migration in an integrin (eg, ITGB1 and ITGB3)-dependent manner. RET is biologically active in the absence of ligands, triggering apoptosis. Thus, RET acts as a classical dependence receptor. In the presence of the ligand GDNF, RET promotes survival and down-regulates growth hormone production. In the absence of GDNF, RET triggers cell death via apoptosis.

Overall, gene expression profiling experiments indicate that there are 2 principal biologically meaningful mechanisms explaining how drugs may cause the potent anticancer effects: (1) molecular interference with multiple signaling pathways specified in the reports and (2) molecular mimicry of the effects of specific genes, in particular, genes with known functions as tumor suppressors, defined by gene silencing and/or gene overexpression experiments. It is important to note that fb-PMT exhibited significant inhibitory effects on the Hedgehog pathway, which are very important to overcome TMZ resistance in GBM patien**ts.**

## Discussion

The integrin αvβ3 plays a critical role in glioblastoma-associated biological processes, making it an important target for the development of novel targeted ligands. GBM is a thyroid hormone-dependent tumor and this effect was mediated at least in part via non-genomic actions at the cell-surface receptor on integrin αvβ3.^35^ Although various integrin ligands have been reported, most of them are universal ligands for multiple integrin receptors and show limited binding affinity for integrin αvβ3. A number of in vitro and in vivo studies have supported a role of thyroid hormones (l-thyroxine, T_4_; 3,5,3′-triido-L-thyronine, T_3_) in the proliferation of tumor cells. Recently we showed that the incorporation of a triazole group and PEG molecules within P-bi-TAT and without any change to the carboxylic acid group significantly increased the integrin binding affinity compared to tetrac.^36^ Our group previously compared the potency of Nano Di Aminopropane Tetrac (NDAT) in in vitro and in vivo studies using U87 glioblastoma cells as well as different tumors that express αvβ3.^29,30^ Broad Spectrum Antiangiogenesis Activities for fb-PMT versus the anti-VEGF bevacizumab (Avastin) was demonstrated suggesting a potentially more effective strategy in combating pathological angiogenesis (devascularization) in GBM as well as potentially reduce edema via its anti-inflammatory activity as demonstrated from the modulation pro-inflammatory genomic pathways.

In the past decade, our studies have shown the αvβ3-dependent antiproliferative, anti-angiogenic, and anticancer properties of agonist thyroid hormones (T_4_ and T_3_) by tetrac in various cancer types. Cody et al. showed that unmodified tetrac may penetrate into cells and can interact with the Thyroid hormone nuclear receptor to where it is a low potency agonist (thyromimetic).^37^ On the other hand, fb-PMT does not gain access to the cell nucleus and shows a more robust antiproliferative effect. In our previous studies, we showed that increasing PEG substitution can lower the binding affinities of different therapeutics.^38–41^ In earlier in vitro studies, we showed that 3 rodent glioma cell lines proliferated in response to thyroid hormone that is blocked by tetrac.^26^ Beyond antiproliferation at the level of the tumor cell, a second important facet of the properties of αvβ3 antagonists is that they have anticancer efficacy by multiple mechanisms. Here, the in vivo antitumor efficacies of the αvβ3 antagonists were evaluated in U87-luc glioblastoma tumor-bearing nude mice, and the compounds significantly reduced tumor volumes and impaired tumor growth in a dose-dependent manner by suppressing angiogenesis (ON Treatment). In the second group of treated mice, the xenografts were observed for an additional 21 days with no further treatment (ON + OFF Treatment). No regrowth of the tumor was observed, and the absence of cell viability persisted. One explanation for these observed effects is that tetrac impairs tumor growth by blocking angiogenesis and by impairing the endothelial cell function rather than by impeding tumor cell growth directly. We have ascribed conventional pro-apoptotic activity to tetrac which would account for the progressive decrease in tumor volume that occurred over 21 days of treatment. Additional in vivo studies with an orthotopic brain tumor are planned.

Our group previously formulated a polymeric nanoparticle, NDAT against a variety of xenografts. Chemical changes to the tetrac molecule at the outer ring hydroxyl by adding a triazole and PEG molecule did not allow the agent to gain access to the cell interior and thus the tetrac ether-bonded to the PLGA particle *via* the outer ring hydroxyl can act only at the integrin receptor where it is exclusively an antagonist and not at the nuclear receptor for thyroid hormone. Furthermore, the histopathological sections represent that the extensive necrosis induced in the tumor mass is present in all treated tumors, causing apoptosis.

We have also developed convincing evidence that fb-PMT crosses the blood-brain barrier. The luminescent signals of the single molecular target on αvβ3, the target that when activated by chemically modified tetrac, regulates a network of intracellular signaling pathways and plasma membrane functions and further control specific gene transcription and cell surface vascular growth factor receptor functions that are highly relevant to cancer and cancer-linked angiogenesis. Previous studies showed that αvβ3 antagonists multivalency results in increased binding affinity, which then improved targeted therapeutic delivery.^26,42,43^ However, despite many studies over years there are no reports that have demonstrated improved therapeutic effectiveness of dimer αvβ3 antagonists over monomer αvβ3 antagonists.

RNA-seq analyses of fb-PMT effects on gene expression in GBM cells demonstrated consistent evidence of molecular interferences with gene expression pathways implicated in tumor progression, cancer cells’ growth and survival, and tumor neovascularization. Among the genes whose expression is downregulated in tumor cells following fb-PMT treatment, are MAPK1, AKT, Hedgehog, NOTCH1, and Wnt, all of which encode products involved in intracellular signaling. The respective gene products are related to cell division and cell migration. Examples of genes upregulated by fb-PMT include BAD, BID, and BAX, the protein products of which are pro-apoptotic. The examples of genomic actions of fb-PMT of potential relevance for inhibition of neovascularization include decreased expression of FGFR1, EGFR, and components of the downstream signaling pathways for VEGFA, VEGFR2, and PDGF.

Currently, there is no efficient single molecular target-tailored therapy against GBM because the cancer cells rely on so many different growth and survival pathways. Our extensive preclinical pharmacological, pharmacokinetic and pharmacodynamic studies show that fb-PMT treatment exerts remarkable molecular interference effects on several growth and survival pathways even when examined in a single-cell line model of human cancer. The broad-spectrum antiangiogenesis activities of fb-PMT, induced by multiple distinct endogenous ligands, offer a distinct advantage over single-mechanism anti-angiogenesis drugs.

### Limitations

PK and biodistribution data in the brain GBM implant in nude mice was performed using U87 cells and further validation in the future using patient-derived glioblastoma cells or transgenic mouse models is warranted.

## Conclusion

In conclusion, we found that the thyrointegrin avb3 antagonist fb-PMT is potent, selective vs other integrins, penetrates the blood-brain barrier via a thyroid hormone transporter, localizes in GBM tumors, and is extremely effective in vivo rodent efficacy models. It reverses angiogenesis stimulated by multiple routes, and gene expression profiling experiments found that fb-PMT dosing affects the expression of a number of genes that are important in cancer progression.

## Supplementary Material

vdac180_suppl_Supplementary_Materials

## Data Availability

All data are available and stored at PRI intranet. All materials related to the project are available at PRI laboratories.
