# Supplementary material for: Discovery of novel thyrointegrin αvβ3 antagonist fb-PMT (NP751) in the management of human glioblastoma multiforme
Source: Neurooncol Adv. 2022 Dec 8;5(1):vdac180. doi: 10.1093/noajnl/vdac180 (PMC9985163; doi:10.1093/noajnl/vdac180)
Supplement: vdac180_suppl_Supplementary_Materials [file vdac180_suppl_supplementary_materials.zip › Supplemental Summary SS1. GSEA of DEGs fb_PMT U87 RNA_seq.pptx]

## Slide 1
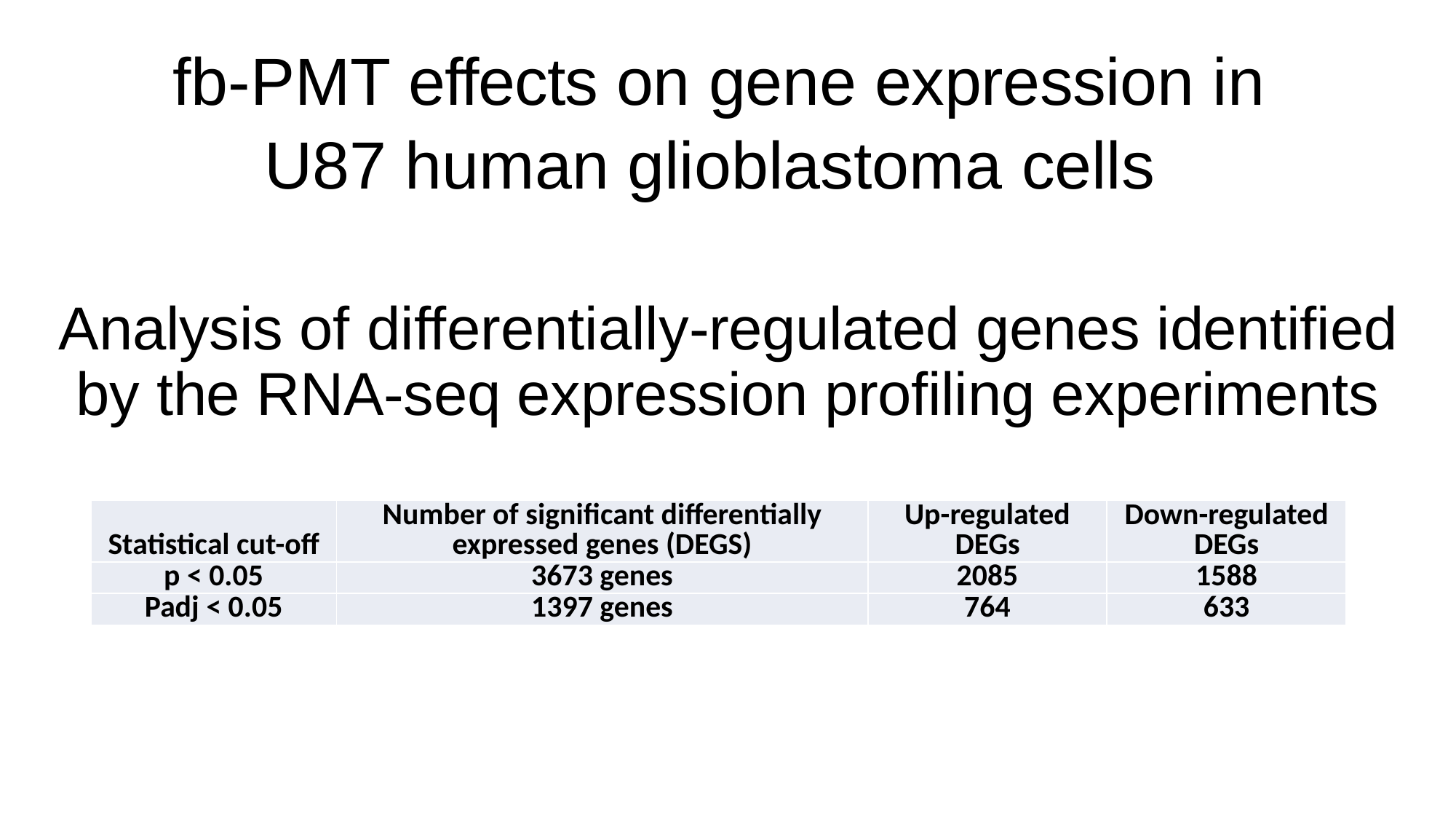

fb-PMT effects on gene expression in
U87 human glioblastoma cells
Analysis of differentially-regulated genes identified by the RNA-seq expression profiling experiments
| Statistical cut-off | Number of significant differentially expressed genes (DEGS) | Up-regulated DEGs | Down-regulated DEGs |
| --- | --- | --- | --- |
| p < 0.05 | 3673 genes | 2085 | 1588 |
| Padj < 0.05 | 1397 genes | 764 | 633 |

## Slide 2
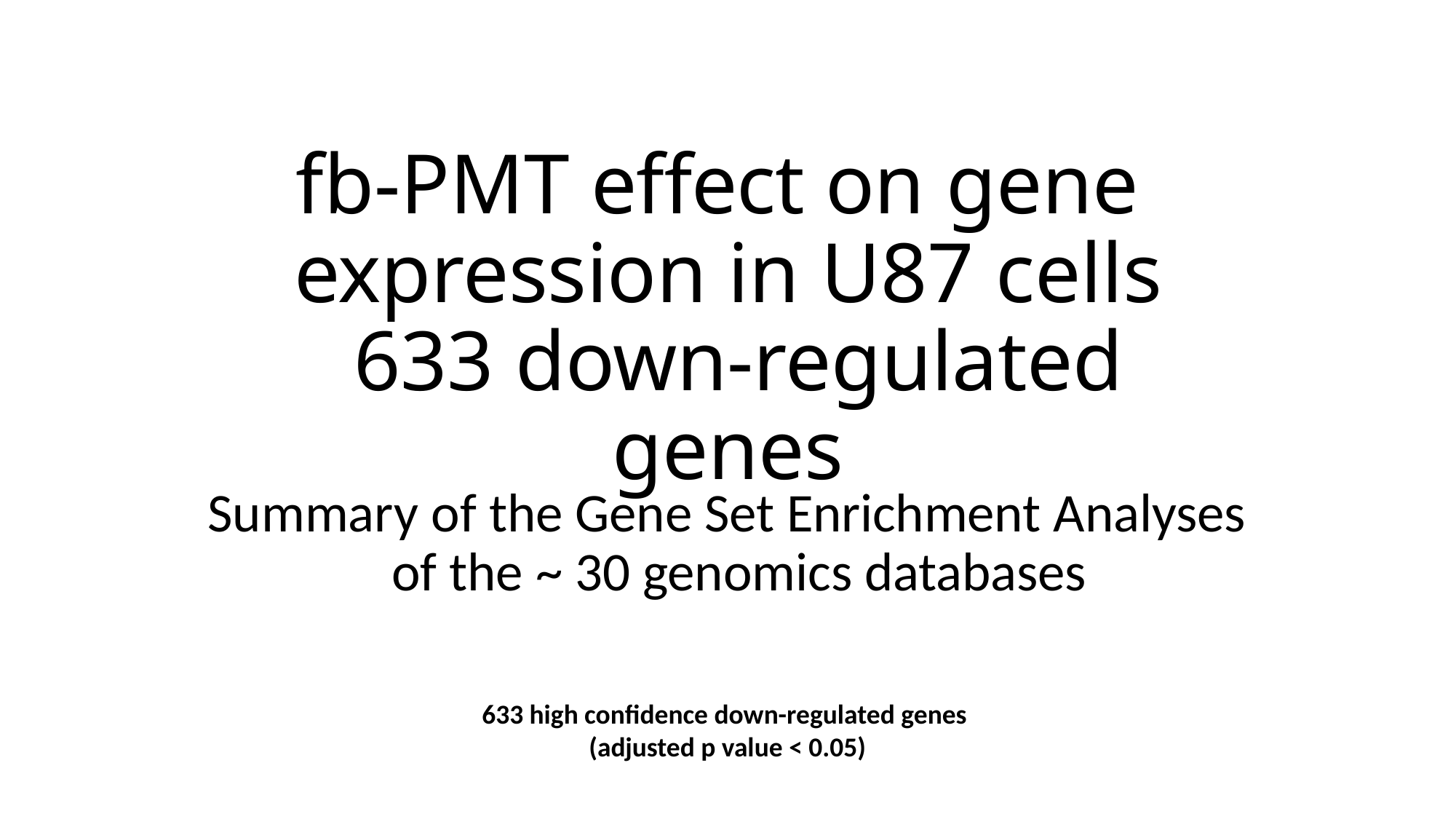

fb-PMT effect on gene expression in U87 cells 633 down-regulated genes
Summary of the Gene Set Enrichment Analyses of the ~ 30 genomics databases
633 high confidence down-regulated genes
(adjusted p value < 0.05)

## Slide 3
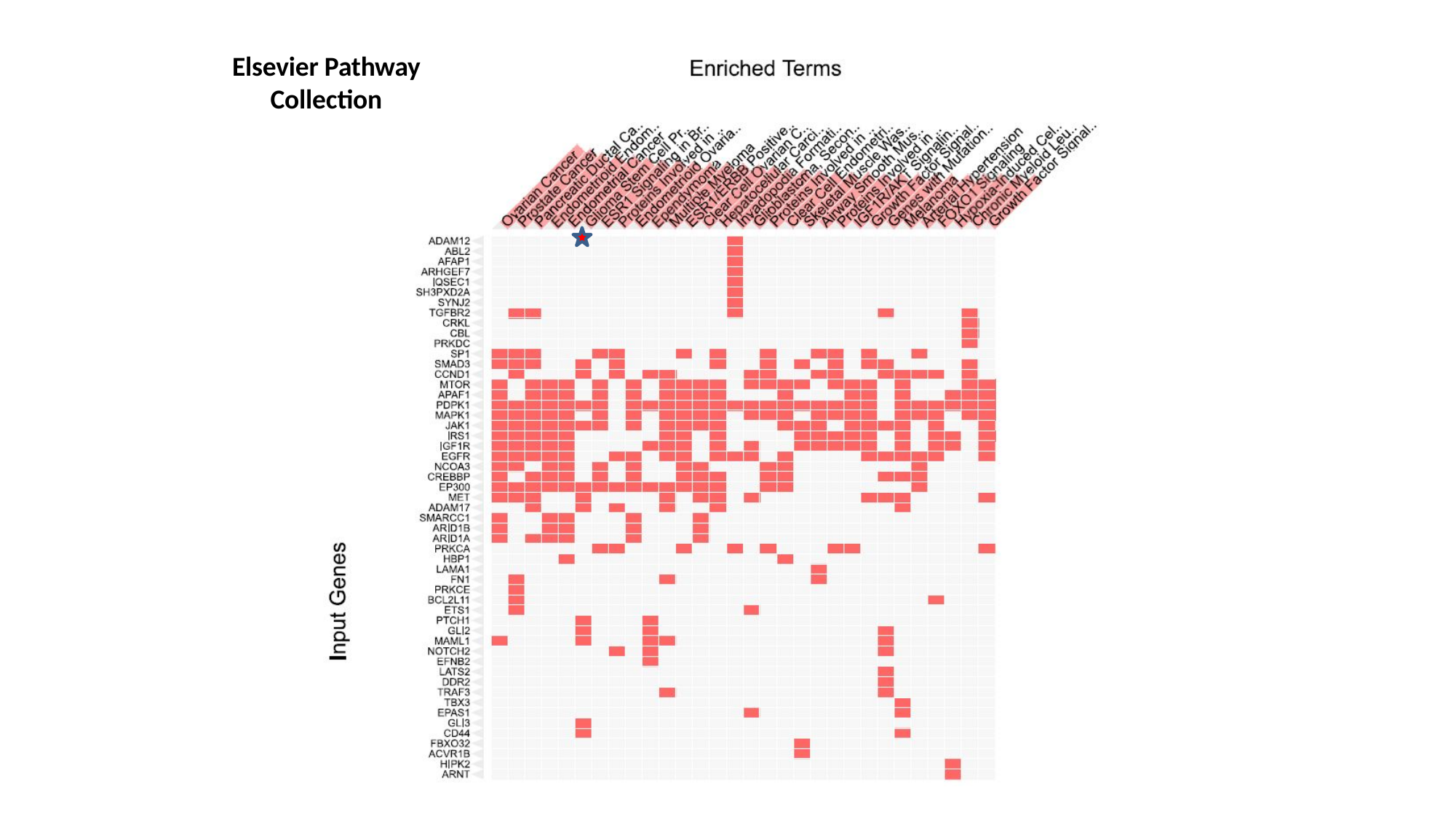

Elsevier Pathway Collection

## Slide 4
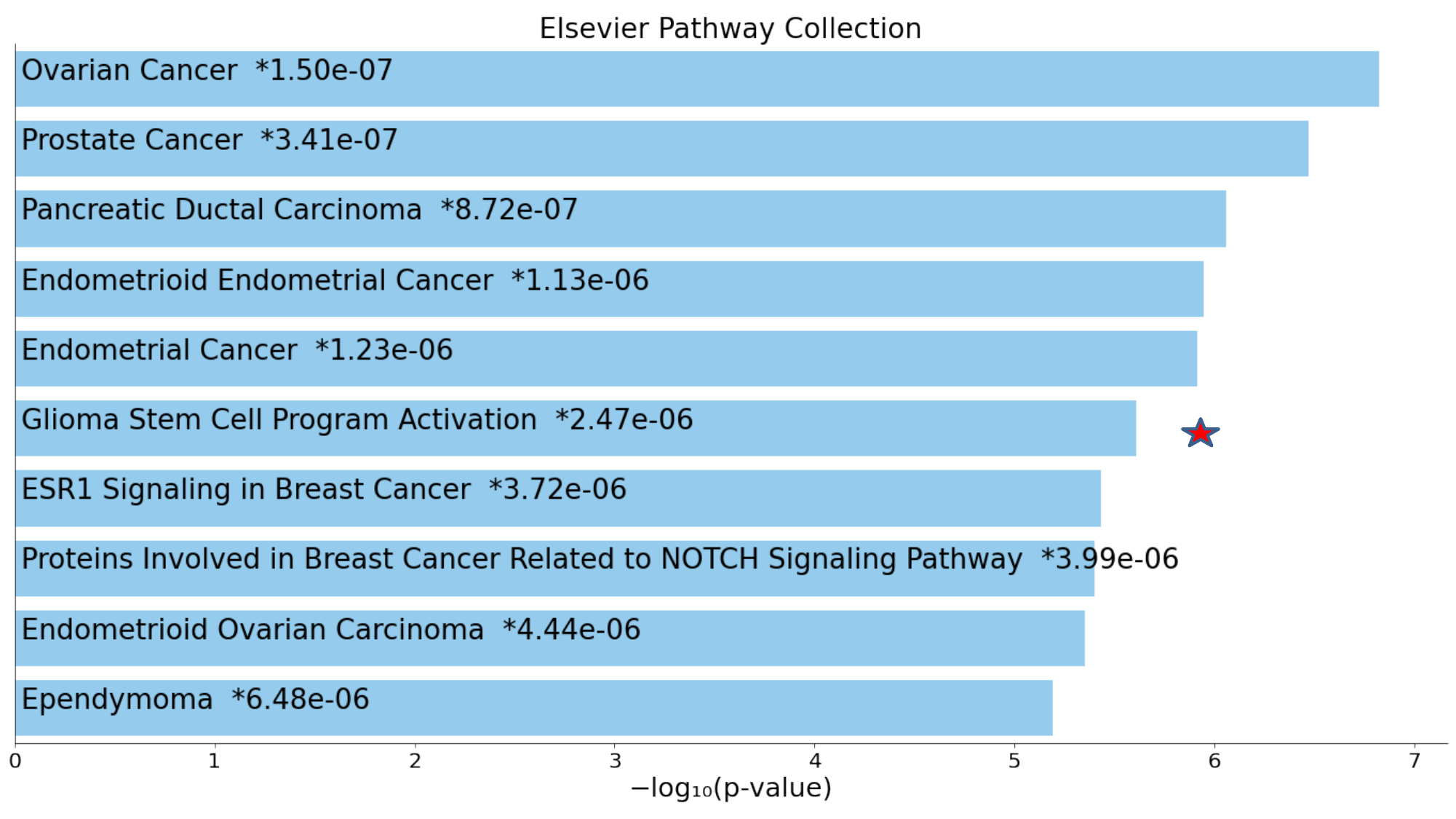

## Slide 5
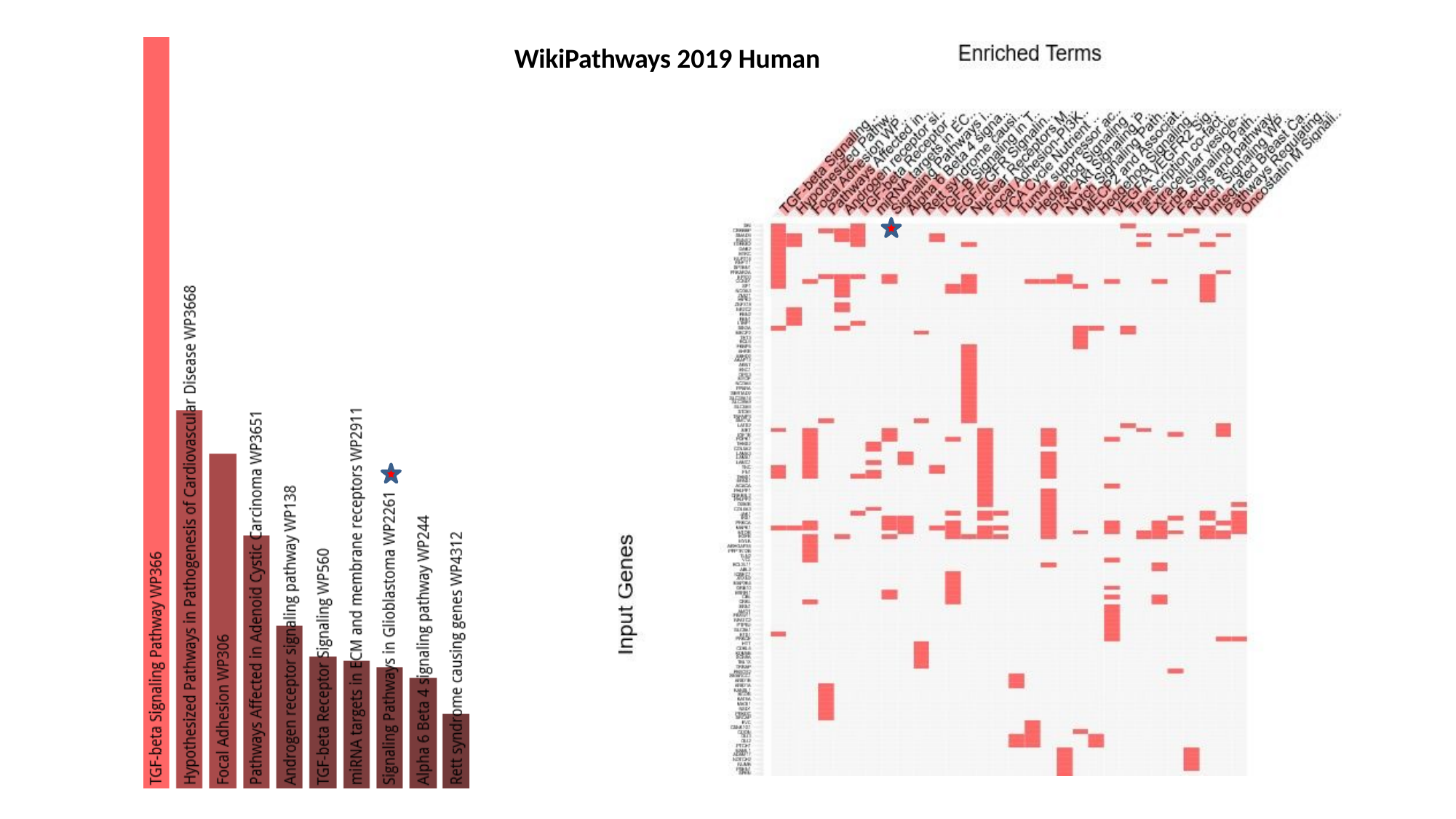

WikiPathways 2019 Human

## Slide 6
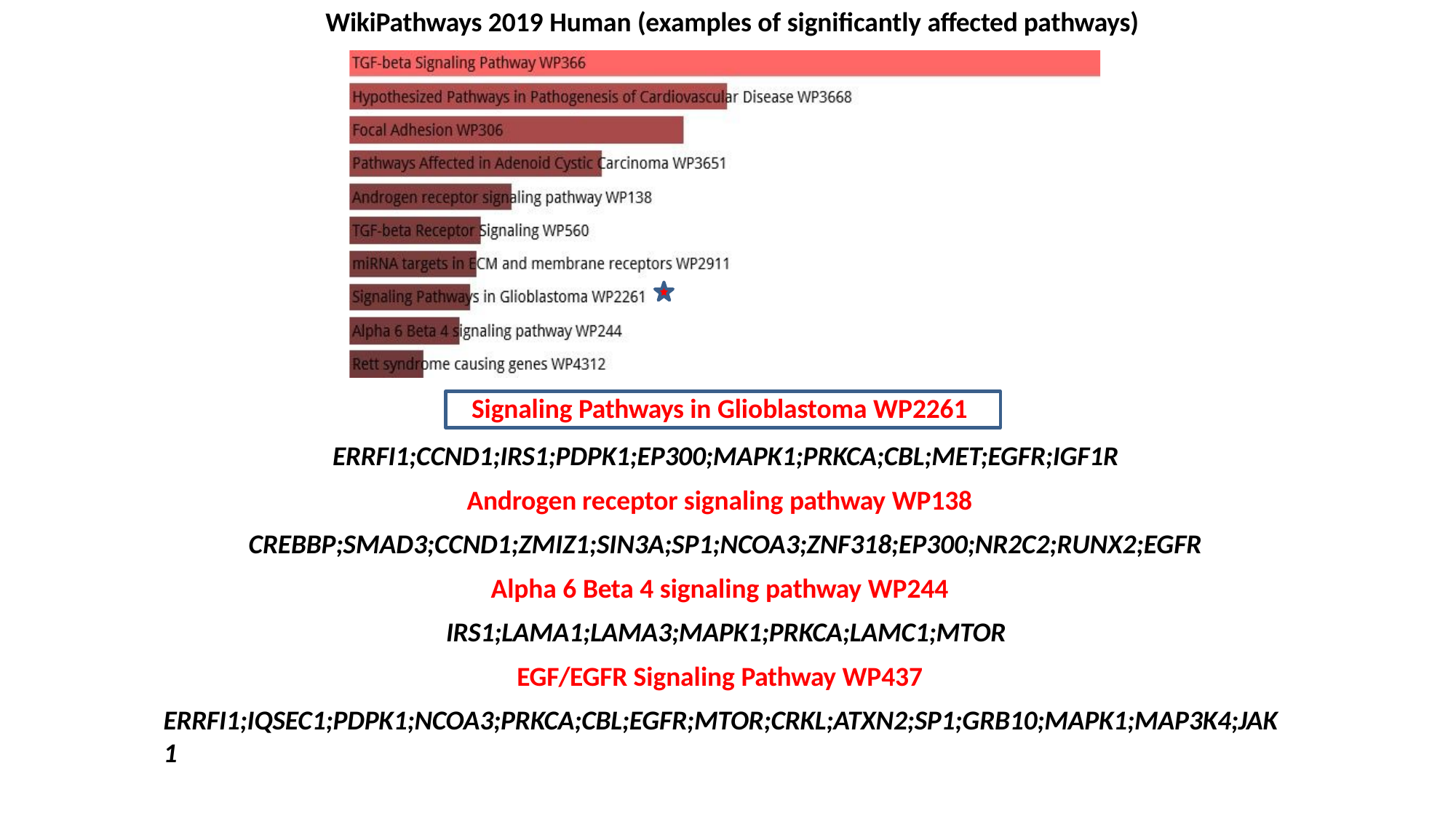

# WikiPathways 2019 Human (examples of significantly affected pathways)
Signaling Pathways in Glioblastoma WP2261
ERRFI1;CCND1;IRS1;PDPK1;EP300;MAPK1;PRKCA;CBL;MET;EGFR;IGF1R
Androgen receptor signaling pathway WP138
CREBBP;SMAD3;CCND1;ZMIZ1;SIN3A;SP1;NCOA3;ZNF318;EP300;NR2C2;RUNX2;EGFR
Alpha 6 Beta 4 signaling pathway WP244
IRS1;LAMA1;LAMA3;MAPK1;PRKCA;LAMC1;MTOR
EGF/EGFR Signaling Pathway WP437
ERRFI1;IQSEC1;PDPK1;NCOA3;PRKCA;CBL;EGFR;MTOR;CRKL;ATXN2;SP1;GRB10;MAPK1;MAP3K4;JAK1

## Slide 7
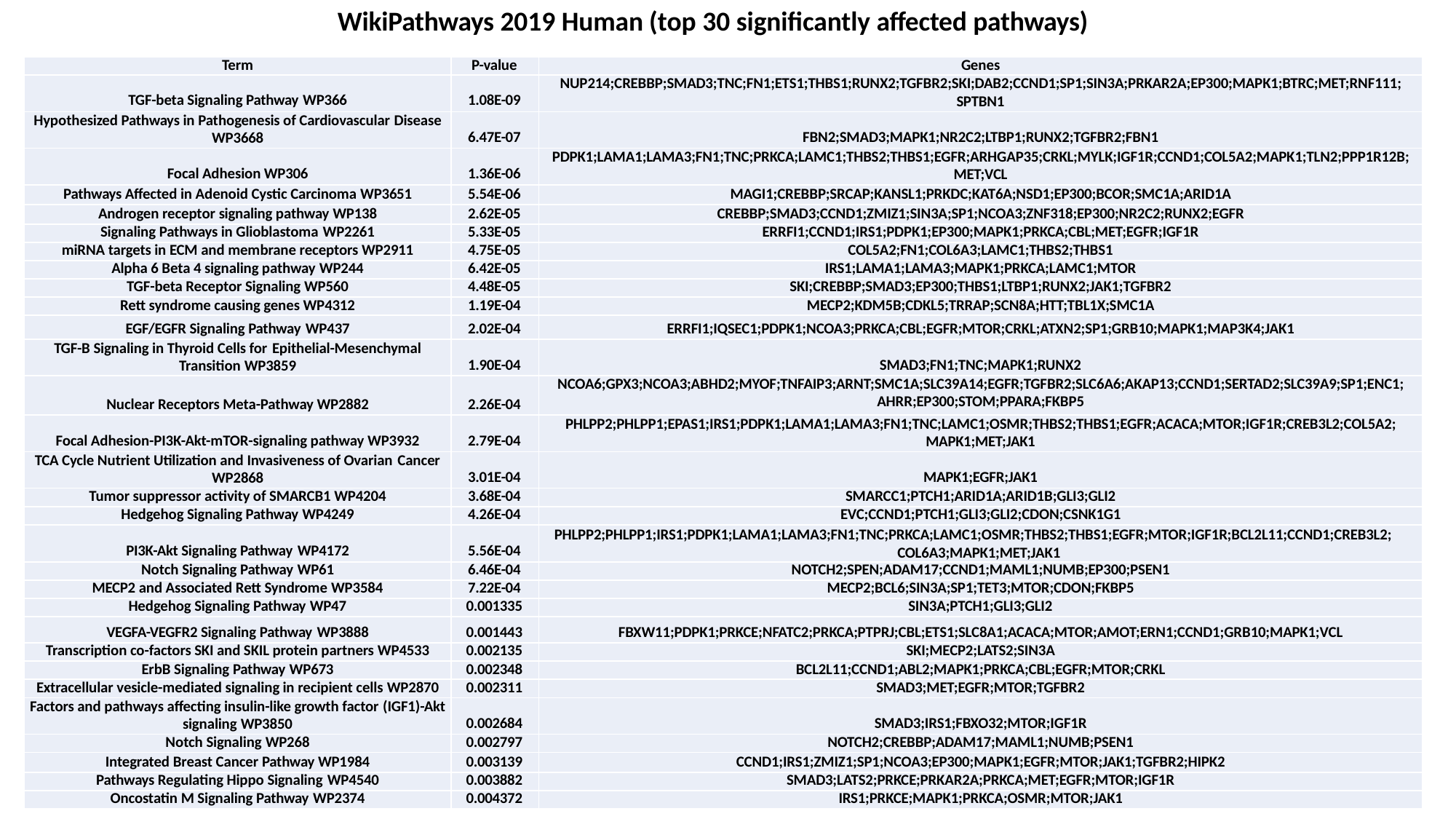

WikiPathways 2019 Human (top 30 significantly affected pathways)
| Term | P-value | Genes |
| --- | --- | --- |
| TGF-beta Signaling Pathway WP366 | 1.08E-09 | NUP214;CREBBP;SMAD3;TNC;FN1;ETS1;THBS1;RUNX2;TGFBR2;SKI;DAB2;CCND1;SP1;SIN3A;PRKAR2A;EP300;MAPK1;BTRC;MET;RNF111; SPTBN1 |
| Hypothesized Pathways in Pathogenesis of Cardiovascular Disease WP3668 | 6.47E-07 | FBN2;SMAD3;MAPK1;NR2C2;LTBP1;RUNX2;TGFBR2;FBN1 |
| Focal Adhesion WP306 | 1.36E-06 | PDPK1;LAMA1;LAMA3;FN1;TNC;PRKCA;LAMC1;THBS2;THBS1;EGFR;ARHGAP35;CRKL;MYLK;IGF1R;CCND1;COL5A2;MAPK1;TLN2;PPP1R12B; MET;VCL |
| Pathways Affected in Adenoid Cystic Carcinoma WP3651 | 5.54E-06 | MAGI1;CREBBP;SRCAP;KANSL1;PRKDC;KAT6A;NSD1;EP300;BCOR;SMC1A;ARID1A |
| Androgen receptor signaling pathway WP138 | 2.62E-05 | CREBBP;SMAD3;CCND1;ZMIZ1;SIN3A;SP1;NCOA3;ZNF318;EP300;NR2C2;RUNX2;EGFR |
| Signaling Pathways in Glioblastoma WP2261 | 5.33E-05 | ERRFI1;CCND1;IRS1;PDPK1;EP300;MAPK1;PRKCA;CBL;MET;EGFR;IGF1R |
| miRNA targets in ECM and membrane receptors WP2911 | 4.75E-05 | COL5A2;FN1;COL6A3;LAMC1;THBS2;THBS1 |
| Alpha 6 Beta 4 signaling pathway WP244 | 6.42E-05 | IRS1;LAMA1;LAMA3;MAPK1;PRKCA;LAMC1;MTOR |
| TGF-beta Receptor Signaling WP560 | 4.48E-05 | SKI;CREBBP;SMAD3;EP300;THBS1;LTBP1;RUNX2;JAK1;TGFBR2 |
| Rett syndrome causing genes WP4312 | 1.19E-04 | MECP2;KDM5B;CDKL5;TRRAP;SCN8A;HTT;TBL1X;SMC1A |
| EGF/EGFR Signaling Pathway WP437 | 2.02E-04 | ERRFI1;IQSEC1;PDPK1;NCOA3;PRKCA;CBL;EGFR;MTOR;CRKL;ATXN2;SP1;GRB10;MAPK1;MAP3K4;JAK1 |
| TGF-B Signaling in Thyroid Cells for Epithelial-Mesenchymal Transition WP3859 | 1.90E-04 | SMAD3;FN1;TNC;MAPK1;RUNX2 |
| Nuclear Receptors Meta-Pathway WP2882 | 2.26E-04 | NCOA6;GPX3;NCOA3;ABHD2;MYOF;TNFAIP3;ARNT;SMC1A;SLC39A14;EGFR;TGFBR2;SLC6A6;AKAP13;CCND1;SERTAD2;SLC39A9;SP1;ENC1; AHRR;EP300;STOM;PPARA;FKBP5 |
| Focal Adhesion-PI3K-Akt-mTOR-signaling pathway WP3932 | 2.79E-04 | PHLPP2;PHLPP1;EPAS1;IRS1;PDPK1;LAMA1;LAMA3;FN1;TNC;LAMC1;OSMR;THBS2;THBS1;EGFR;ACACA;MTOR;IGF1R;CREB3L2;COL5A2; MAPK1;MET;JAK1 |
| TCA Cycle Nutrient Utilization and Invasiveness of Ovarian Cancer WP2868 | 3.01E-04 | MAPK1;EGFR;JAK1 |
| Tumor suppressor activity of SMARCB1 WP4204 | 3.68E-04 | SMARCC1;PTCH1;ARID1A;ARID1B;GLI3;GLI2 |
| Hedgehog Signaling Pathway WP4249 | 4.26E-04 | EVC;CCND1;PTCH1;GLI3;GLI2;CDON;CSNK1G1 |
| PI3K-Akt Signaling Pathway WP4172 | 5.56E-04 | PHLPP2;PHLPP1;IRS1;PDPK1;LAMA1;LAMA3;FN1;TNC;PRKCA;LAMC1;OSMR;THBS2;THBS1;EGFR;MTOR;IGF1R;BCL2L11;CCND1;CREB3L2; COL6A3;MAPK1;MET;JAK1 |
| Notch Signaling Pathway WP61 | 6.46E-04 | NOTCH2;SPEN;ADAM17;CCND1;MAML1;NUMB;EP300;PSEN1 |
| MECP2 and Associated Rett Syndrome WP3584 | 7.22E-04 | MECP2;BCL6;SIN3A;SP1;TET3;MTOR;CDON;FKBP5 |
| Hedgehog Signaling Pathway WP47 | 0.001335 | SIN3A;PTCH1;GLI3;GLI2 |
| VEGFA-VEGFR2 Signaling Pathway WP3888 | 0.001443 | FBXW11;PDPK1;PRKCE;NFATC2;PRKCA;PTPRJ;CBL;ETS1;SLC8A1;ACACA;MTOR;AMOT;ERN1;CCND1;GRB10;MAPK1;VCL |
| Transcription co-factors SKI and SKIL protein partners WP4533 | 0.002135 | SKI;MECP2;LATS2;SIN3A |
| ErbB Signaling Pathway WP673 | 0.002348 | BCL2L11;CCND1;ABL2;MAPK1;PRKCA;CBL;EGFR;MTOR;CRKL |
| Extracellular vesicle-mediated signaling in recipient cells WP2870 | 0.002311 | SMAD3;MET;EGFR;MTOR;TGFBR2 |
| Factors and pathways affecting insulin-like growth factor (IGF1)-Akt signaling WP3850 | 0.002684 | SMAD3;IRS1;FBXO32;MTOR;IGF1R |
| Notch Signaling WP268 | 0.002797 | NOTCH2;CREBBP;ADAM17;MAML1;NUMB;PSEN1 |
| Integrated Breast Cancer Pathway WP1984 | 0.003139 | CCND1;IRS1;ZMIZ1;SP1;NCOA3;EP300;MAPK1;EGFR;MTOR;JAK1;TGFBR2;HIPK2 |
| Pathways Regulating Hippo Signaling WP4540 | 0.003882 | SMAD3;LATS2;PRKCE;PRKAR2A;PRKCA;MET;EGFR;MTOR;IGF1R |
| Oncostatin M Signaling Pathway WP2374 | 0.004372 | IRS1;PRKCE;MAPK1;PRKCA;OSMR;MTOR;JAK1 |

## Slide 8
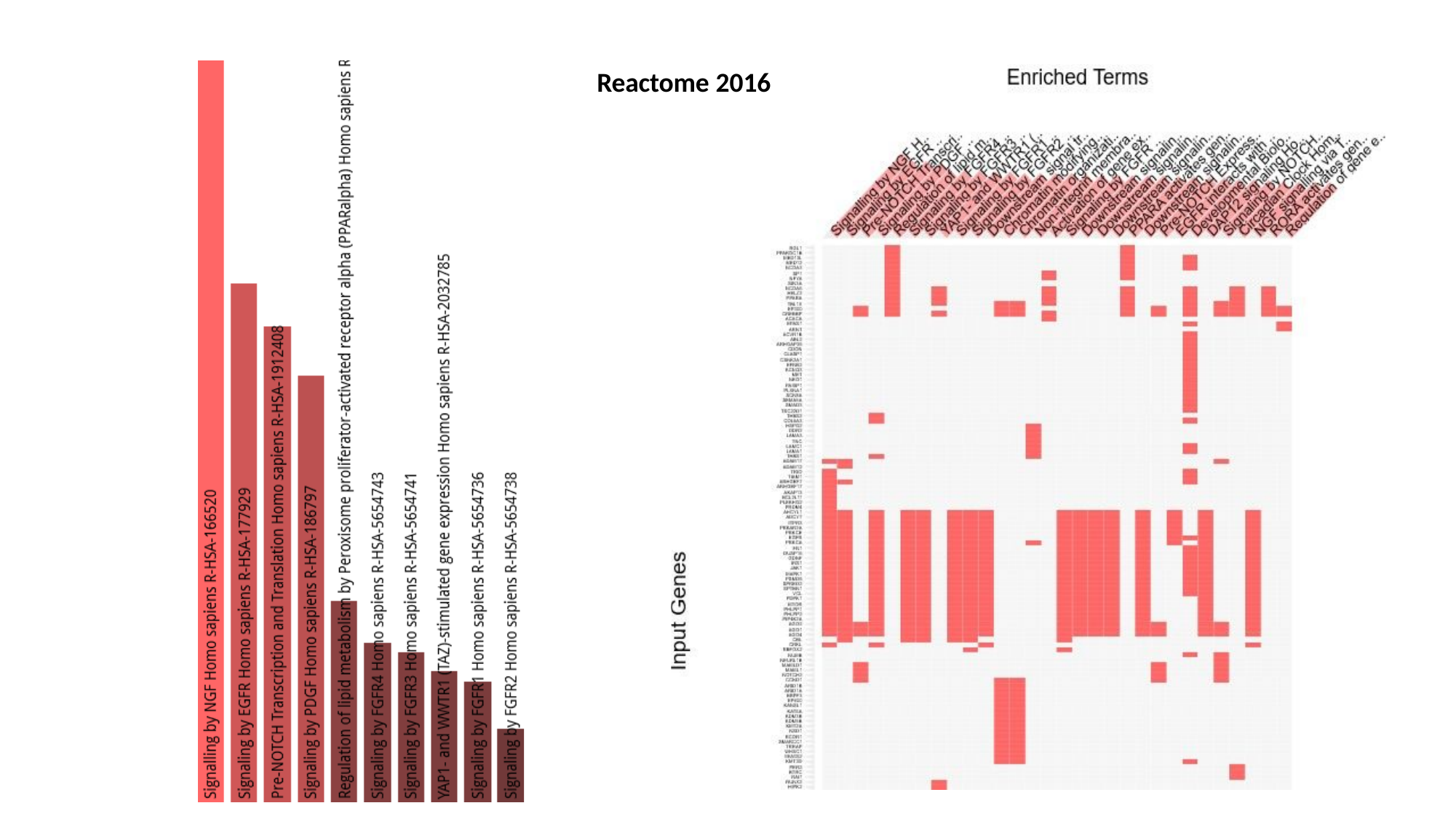

Reactome 2016

## Slide 9
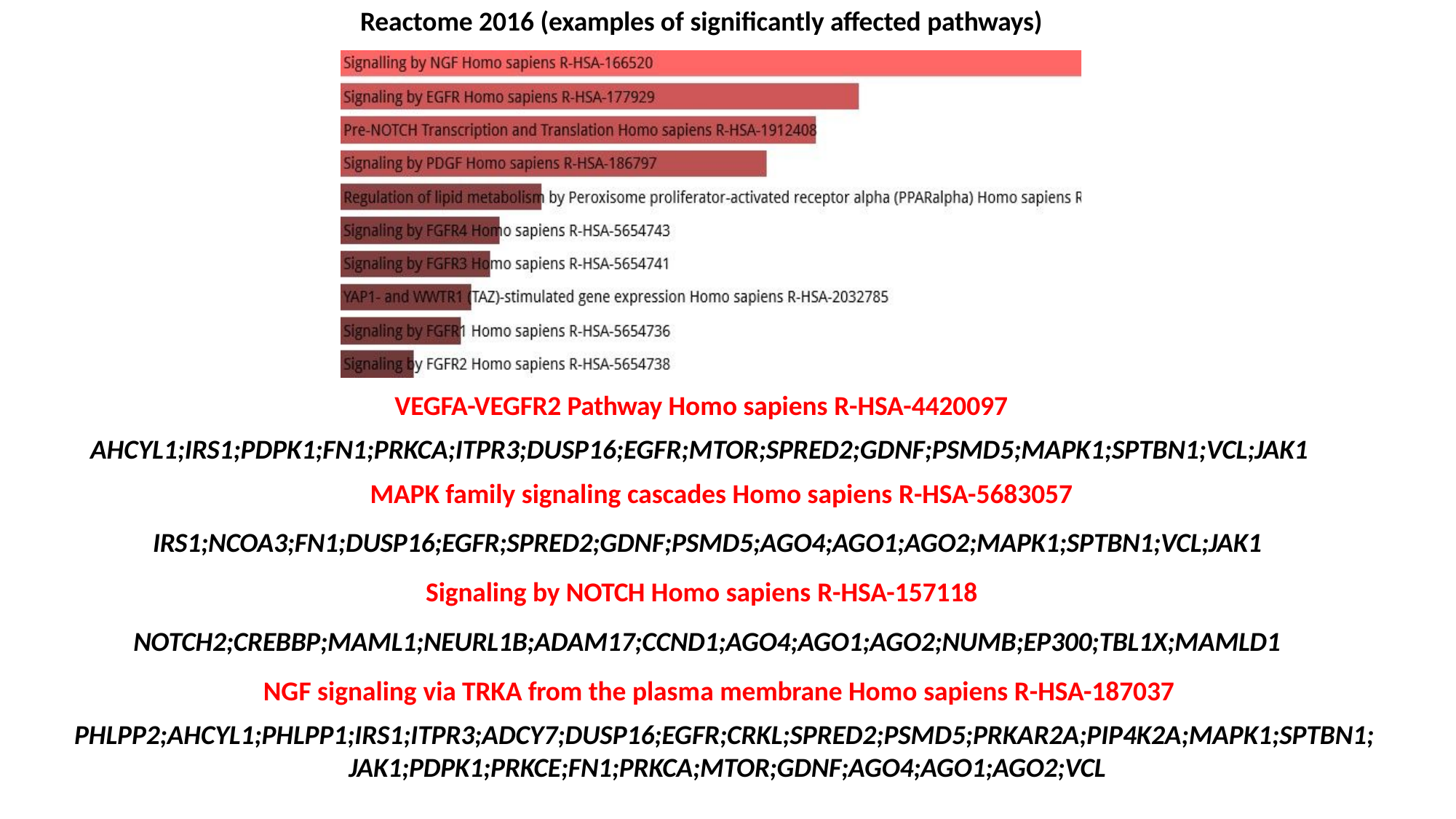

# Reactome 2016 (examples of significantly affected pathways)
VEGFA-VEGFR2 Pathway Homo sapiens R-HSA-4420097
AHCYL1;IRS1;PDPK1;FN1;PRKCA;ITPR3;DUSP16;EGFR;MTOR;SPRED2;GDNF;PSMD5;MAPK1;SPTBN1;VCL;JAK1
MAPK family signaling cascades Homo sapiens R-HSA-5683057
IRS1;NCOA3;FN1;DUSP16;EGFR;SPRED2;GDNF;PSMD5;AGO4;AGO1;AGO2;MAPK1;SPTBN1;VCL;JAK1
Signaling by NOTCH Homo sapiens R-HSA-157118
NOTCH2;CREBBP;MAML1;NEURL1B;ADAM17;CCND1;AGO4;AGO1;AGO2;NUMB;EP300;TBL1X;MAMLD1
NGF signaling via TRKA from the plasma membrane Homo sapiens R-HSA-187037
PHLPP2;AHCYL1;PHLPP1;IRS1;ITPR3;ADCY7;DUSP16;EGFR;CRKL;SPRED2;PSMD5;PRKAR2A;PIP4K2A;MAPK1;SPTBN1; JAK1;PDPK1;PRKCE;FN1;PRKCA;MTOR;GDNF;AGO4;AGO1;AGO2;VCL

## Slide 10
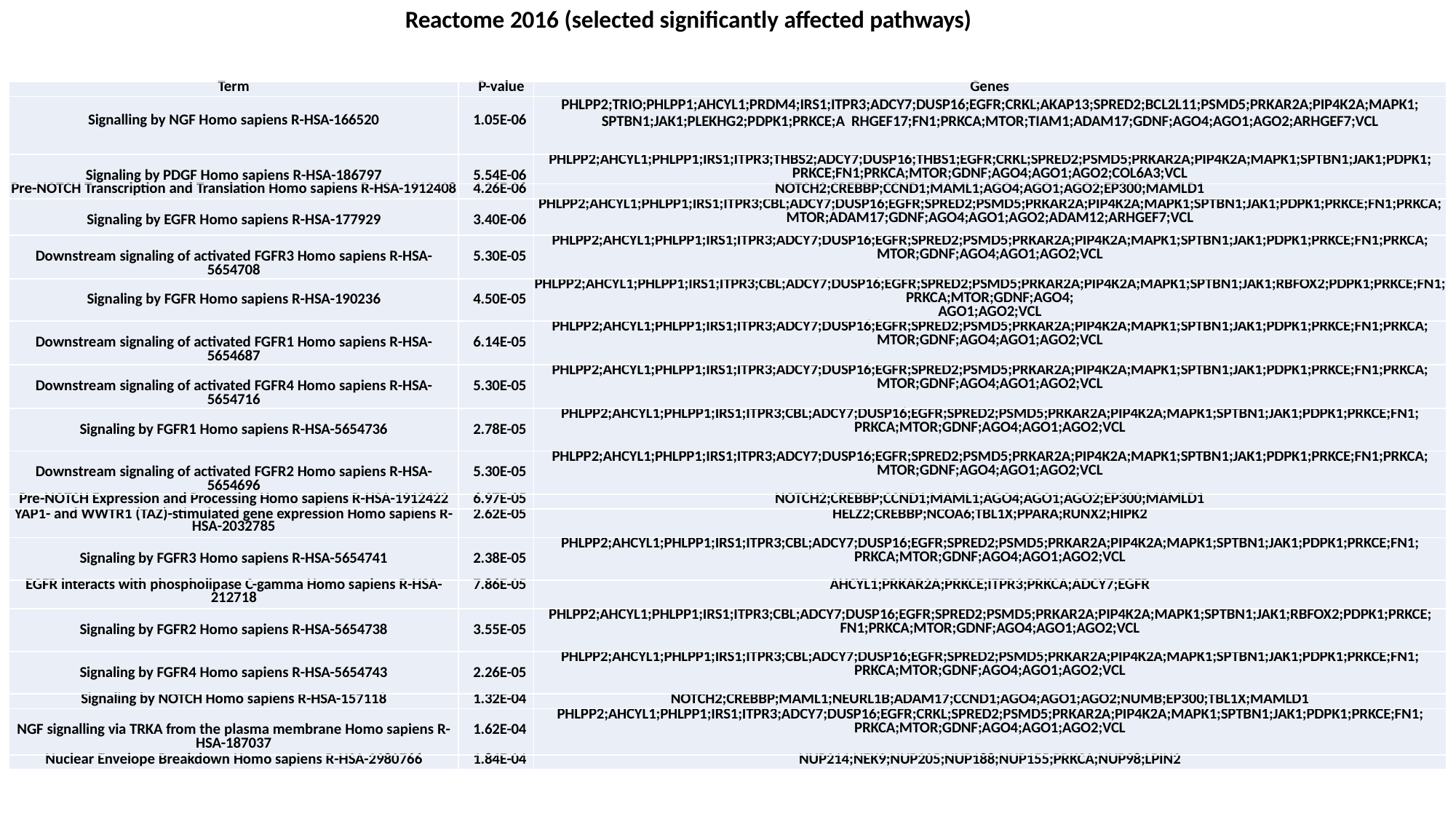

Reactome 2016 (selected significantly affected pathways)
| Term | P-value | Genes |
| --- | --- | --- |
| Signalling by NGF Homo sapiens R-HSA-166520 | 1.05E-06 | PHLPP2;TRIO;PHLPP1;AHCYL1;PRDM4;IRS1;ITPR3;ADCY7;DUSP16;EGFR;CRKL;AKAP13;SPRED2;BCL2L11;PSMD5;PRKAR2A;PIP4K2A;MAPK1; SPTBN1;JAK1;PLEKHG2;PDPK1;PRKCE;A RHGEF17;FN1;PRKCA;MTOR;TIAM1;ADAM17;GDNF;AGO4;AGO1;AGO2;ARHGEF7;VCL |
| Signaling by PDGF Homo sapiens R-HSA-186797 | 5.54E-06 | PHLPP2;AHCYL1;PHLPP1;IRS1;ITPR3;THBS2;ADCY7;DUSP16;THBS1;EGFR;CRKL;SPRED2;PSMD5;PRKAR2A;PIP4K2A;MAPK1;SPTBN1;JAK1;PDPK1; PRKCE;FN1;PRKCA;MTOR;GDNF;AGO4;AGO1;AGO2;COL6A3;VCL |
| Pre-NOTCH Transcription and Translation Homo sapiens R-HSA-1912408 | 4.26E-06 | NOTCH2;CREBBP;CCND1;MAML1;AGO4;AGO1;AGO2;EP300;MAMLD1 |
| Signaling by EGFR Homo sapiens R-HSA-177929 | 3.40E-06 | PHLPP2;AHCYL1;PHLPP1;IRS1;ITPR3;CBL;ADCY7;DUSP16;EGFR;SPRED2;PSMD5;PRKAR2A;PIP4K2A;MAPK1;SPTBN1;JAK1;PDPK1;PRKCE;FN1;PRKCA;MTOR;ADAM17;GDNF;AGO4;AGO1;AGO2;ADAM12;ARHGEF7;VCL |
| Downstream signaling of activated FGFR3 Homo sapiens R-HSA-5654708 | 5.30E-05 | PHLPP2;AHCYL1;PHLPP1;IRS1;ITPR3;ADCY7;DUSP16;EGFR;SPRED2;PSMD5;PRKAR2A;PIP4K2A;MAPK1;SPTBN1;JAK1;PDPK1;PRKCE;FN1;PRKCA; MTOR;GDNF;AGO4;AGO1;AGO2;VCL |
| Signaling by FGFR Homo sapiens R-HSA-190236 | 4.50E-05 | PHLPP2;AHCYL1;PHLPP1;IRS1;ITPR3;CBL;ADCY7;DUSP16;EGFR;SPRED2;PSMD5;PRKAR2A;PIP4K2A;MAPK1;SPTBN1;JAK1;RBFOX2;PDPK1;PRKCE;FN1;PRKCA;MTOR;GDNF;AGO4; AGO1;AGO2;VCL |
| Downstream signaling of activated FGFR1 Homo sapiens R-HSA-5654687 | 6.14E-05 | PHLPP2;AHCYL1;PHLPP1;IRS1;ITPR3;ADCY7;DUSP16;EGFR;SPRED2;PSMD5;PRKAR2A;PIP4K2A;MAPK1;SPTBN1;JAK1;PDPK1;PRKCE;FN1;PRKCA; MTOR;GDNF;AGO4;AGO1;AGO2;VCL |
| Downstream signaling of activated FGFR4 Homo sapiens R-HSA-5654716 | 5.30E-05 | PHLPP2;AHCYL1;PHLPP1;IRS1;ITPR3;ADCY7;DUSP16;EGFR;SPRED2;PSMD5;PRKAR2A;PIP4K2A;MAPK1;SPTBN1;JAK1;PDPK1;PRKCE;FN1;PRKCA; MTOR;GDNF;AGO4;AGO1;AGO2;VCL |
| Signaling by FGFR1 Homo sapiens R-HSA-5654736 | 2.78E-05 | PHLPP2;AHCYL1;PHLPP1;IRS1;ITPR3;CBL;ADCY7;DUSP16;EGFR;SPRED2;PSMD5;PRKAR2A;PIP4K2A;MAPK1;SPTBN1;JAK1;PDPK1;PRKCE;FN1; PRKCA;MTOR;GDNF;AGO4;AGO1;AGO2;VCL |
| Downstream signaling of activated FGFR2 Homo sapiens R-HSA-5654696 | 5.30E-05 | PHLPP2;AHCYL1;PHLPP1;IRS1;ITPR3;ADCY7;DUSP16;EGFR;SPRED2;PSMD5;PRKAR2A;PIP4K2A;MAPK1;SPTBN1;JAK1;PDPK1;PRKCE;FN1;PRKCA; MTOR;GDNF;AGO4;AGO1;AGO2;VCL |
| Pre-NOTCH Expression and Processing Homo sapiens R-HSA-1912422 | 6.97E-05 | NOTCH2;CREBBP;CCND1;MAML1;AGO4;AGO1;AGO2;EP300;MAMLD1 |
| YAP1- and WWTR1 (TAZ)-stimulated gene expression Homo sapiens R-HSA-2032785 | 2.62E-05 | HELZ2;CREBBP;NCOA6;TBL1X;PPARA;RUNX2;HIPK2 |
| Signaling by FGFR3 Homo sapiens R-HSA-5654741 | 2.38E-05 | PHLPP2;AHCYL1;PHLPP1;IRS1;ITPR3;CBL;ADCY7;DUSP16;EGFR;SPRED2;PSMD5;PRKAR2A;PIP4K2A;MAPK1;SPTBN1;JAK1;PDPK1;PRKCE;FN1; PRKCA;MTOR;GDNF;AGO4;AGO1;AGO2;VCL |
| EGFR interacts with phospholipase C-gamma Homo sapiens R-HSA-212718 | 7.86E-05 | AHCYL1;PRKAR2A;PRKCE;ITPR3;PRKCA;ADCY7;EGFR |
| Signaling by FGFR2 Homo sapiens R-HSA-5654738 | 3.55E-05 | PHLPP2;AHCYL1;PHLPP1;IRS1;ITPR3;CBL;ADCY7;DUSP16;EGFR;SPRED2;PSMD5;PRKAR2A;PIP4K2A;MAPK1;SPTBN1;JAK1;RBFOX2;PDPK1;PRKCE; FN1;PRKCA;MTOR;GDNF;AGO4;AGO1;AGO2;VCL |
| Signaling by FGFR4 Homo sapiens R-HSA-5654743 | 2.26E-05 | PHLPP2;AHCYL1;PHLPP1;IRS1;ITPR3;CBL;ADCY7;DUSP16;EGFR;SPRED2;PSMD5;PRKAR2A;PIP4K2A;MAPK1;SPTBN1;JAK1;PDPK1;PRKCE;FN1; PRKCA;MTOR;GDNF;AGO4;AGO1;AGO2;VCL |
| Signaling by NOTCH Homo sapiens R-HSA-157118 | 1.32E-04 | NOTCH2;CREBBP;MAML1;NEURL1B;ADAM17;CCND1;AGO4;AGO1;AGO2;NUMB;EP300;TBL1X;MAMLD1 |
| NGF signalling via TRKA from the plasma membrane Homo sapiens R-HSA-187037 | 1.62E-04 | PHLPP2;AHCYL1;PHLPP1;IRS1;ITPR3;ADCY7;DUSP16;EGFR;CRKL;SPRED2;PSMD5;PRKAR2A;PIP4K2A;MAPK1;SPTBN1;JAK1;PDPK1;PRKCE;FN1; PRKCA;MTOR;GDNF;AGO4;AGO1;AGO2;VCL |
| Nuclear Envelope Breakdown Homo sapiens R-HSA-2980766 | 1.84E-04 | NUP214;NEK9;NUP205;NUP188;NUP155;PRKCA;NUP98;LPIN2 |

## Slide 11
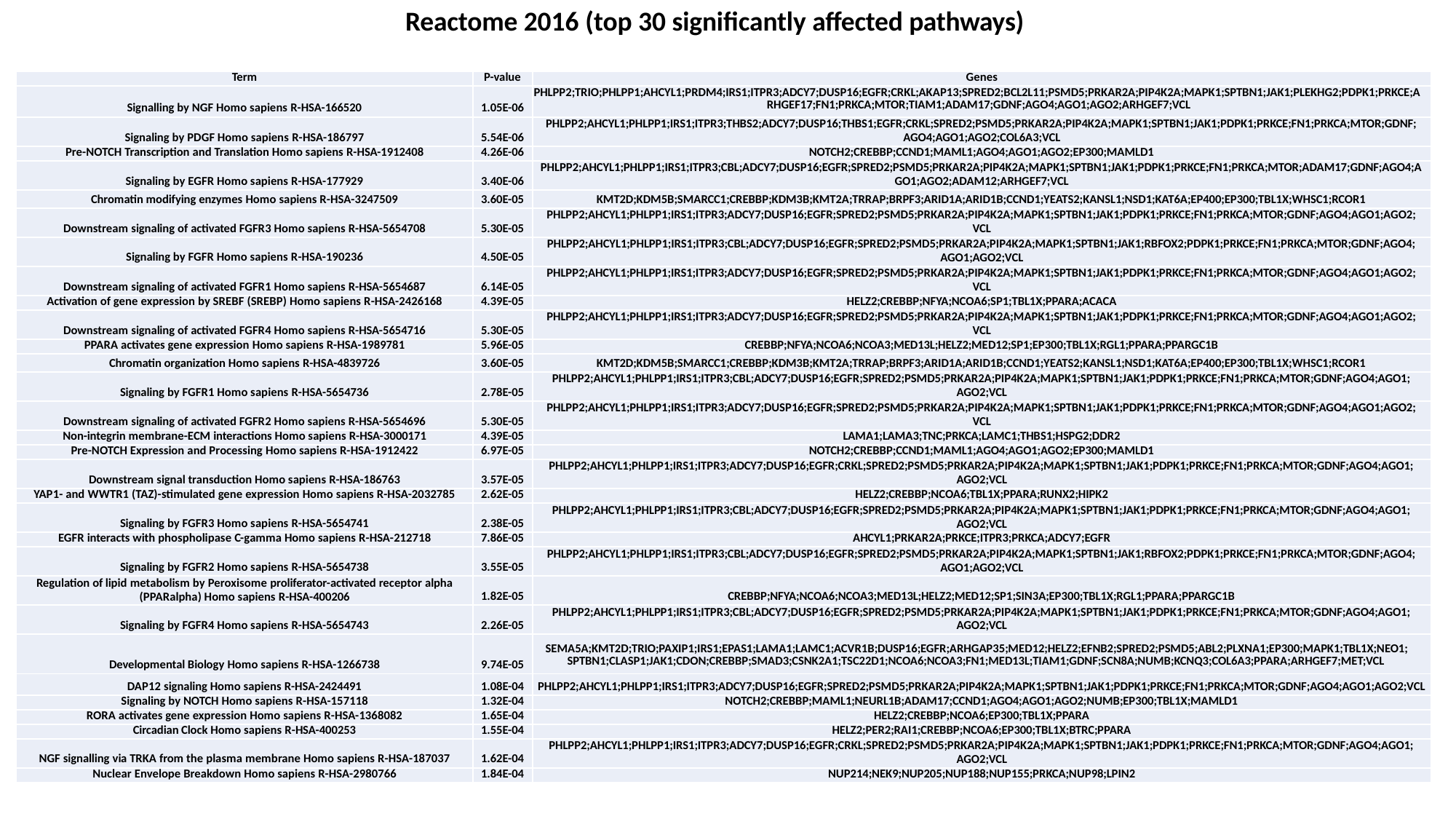

Reactome 2016 (top 30 significantly affected pathways)
| Term | P-value | Genes |
| --- | --- | --- |
| Signalling by NGF Homo sapiens R-HSA-166520 | 1.05E-06 | PHLPP2;TRIO;PHLPP1;AHCYL1;PRDM4;IRS1;ITPR3;ADCY7;DUSP16;EGFR;CRKL;AKAP13;SPRED2;BCL2L11;PSMD5;PRKAR2A;PIP4K2A;MAPK1;SPTBN1;JAK1;PLEKHG2;PDPK1;PRKCE;A RHGEF17;FN1;PRKCA;MTOR;TIAM1;ADAM17;GDNF;AGO4;AGO1;AGO2;ARHGEF7;VCL |
| Signaling by PDGF Homo sapiens R-HSA-186797 | 5.54E-06 | PHLPP2;AHCYL1;PHLPP1;IRS1;ITPR3;THBS2;ADCY7;DUSP16;THBS1;EGFR;CRKL;SPRED2;PSMD5;PRKAR2A;PIP4K2A;MAPK1;SPTBN1;JAK1;PDPK1;PRKCE;FN1;PRKCA;MTOR;GDNF; AGO4;AGO1;AGO2;COL6A3;VCL |
| Pre-NOTCH Transcription and Translation Homo sapiens R-HSA-1912408 | 4.26E-06 | NOTCH2;CREBBP;CCND1;MAML1;AGO4;AGO1;AGO2;EP300;MAMLD1 |
| Signaling by EGFR Homo sapiens R-HSA-177929 | 3.40E-06 | PHLPP2;AHCYL1;PHLPP1;IRS1;ITPR3;CBL;ADCY7;DUSP16;EGFR;SPRED2;PSMD5;PRKAR2A;PIP4K2A;MAPK1;SPTBN1;JAK1;PDPK1;PRKCE;FN1;PRKCA;MTOR;ADAM17;GDNF;AGO4;A GO1;AGO2;ADAM12;ARHGEF7;VCL |
| Chromatin modifying enzymes Homo sapiens R-HSA-3247509 | 3.60E-05 | KMT2D;KDM5B;SMARCC1;CREBBP;KDM3B;KMT2A;TRRAP;BRPF3;ARID1A;ARID1B;CCND1;YEATS2;KANSL1;NSD1;KAT6A;EP400;EP300;TBL1X;WHSC1;RCOR1 |
| Downstream signaling of activated FGFR3 Homo sapiens R-HSA-5654708 | 5.30E-05 | PHLPP2;AHCYL1;PHLPP1;IRS1;ITPR3;ADCY7;DUSP16;EGFR;SPRED2;PSMD5;PRKAR2A;PIP4K2A;MAPK1;SPTBN1;JAK1;PDPK1;PRKCE;FN1;PRKCA;MTOR;GDNF;AGO4;AGO1;AGO2; VCL |
| Signaling by FGFR Homo sapiens R-HSA-190236 | 4.50E-05 | PHLPP2;AHCYL1;PHLPP1;IRS1;ITPR3;CBL;ADCY7;DUSP16;EGFR;SPRED2;PSMD5;PRKAR2A;PIP4K2A;MAPK1;SPTBN1;JAK1;RBFOX2;PDPK1;PRKCE;FN1;PRKCA;MTOR;GDNF;AGO4; AGO1;AGO2;VCL |
| Downstream signaling of activated FGFR1 Homo sapiens R-HSA-5654687 | 6.14E-05 | PHLPP2;AHCYL1;PHLPP1;IRS1;ITPR3;ADCY7;DUSP16;EGFR;SPRED2;PSMD5;PRKAR2A;PIP4K2A;MAPK1;SPTBN1;JAK1;PDPK1;PRKCE;FN1;PRKCA;MTOR;GDNF;AGO4;AGO1;AGO2; VCL |
| Activation of gene expression by SREBF (SREBP) Homo sapiens R-HSA-2426168 | 4.39E-05 | HELZ2;CREBBP;NFYA;NCOA6;SP1;TBL1X;PPARA;ACACA |
| Downstream signaling of activated FGFR4 Homo sapiens R-HSA-5654716 | 5.30E-05 | PHLPP2;AHCYL1;PHLPP1;IRS1;ITPR3;ADCY7;DUSP16;EGFR;SPRED2;PSMD5;PRKAR2A;PIP4K2A;MAPK1;SPTBN1;JAK1;PDPK1;PRKCE;FN1;PRKCA;MTOR;GDNF;AGO4;AGO1;AGO2; VCL |
| PPARA activates gene expression Homo sapiens R-HSA-1989781 | 5.96E-05 | CREBBP;NFYA;NCOA6;NCOA3;MED13L;HELZ2;MED12;SP1;EP300;TBL1X;RGL1;PPARA;PPARGC1B |
| Chromatin organization Homo sapiens R-HSA-4839726 | 3.60E-05 | KMT2D;KDM5B;SMARCC1;CREBBP;KDM3B;KMT2A;TRRAP;BRPF3;ARID1A;ARID1B;CCND1;YEATS2;KANSL1;NSD1;KAT6A;EP400;EP300;TBL1X;WHSC1;RCOR1 |
| Signaling by FGFR1 Homo sapiens R-HSA-5654736 | 2.78E-05 | PHLPP2;AHCYL1;PHLPP1;IRS1;ITPR3;CBL;ADCY7;DUSP16;EGFR;SPRED2;PSMD5;PRKAR2A;PIP4K2A;MAPK1;SPTBN1;JAK1;PDPK1;PRKCE;FN1;PRKCA;MTOR;GDNF;AGO4;AGO1; AGO2;VCL |
| Downstream signaling of activated FGFR2 Homo sapiens R-HSA-5654696 | 5.30E-05 | PHLPP2;AHCYL1;PHLPP1;IRS1;ITPR3;ADCY7;DUSP16;EGFR;SPRED2;PSMD5;PRKAR2A;PIP4K2A;MAPK1;SPTBN1;JAK1;PDPK1;PRKCE;FN1;PRKCA;MTOR;GDNF;AGO4;AGO1;AGO2; VCL |
| Non-integrin membrane-ECM interactions Homo sapiens R-HSA-3000171 | 4.39E-05 | LAMA1;LAMA3;TNC;PRKCA;LAMC1;THBS1;HSPG2;DDR2 |
| Pre-NOTCH Expression and Processing Homo sapiens R-HSA-1912422 | 6.97E-05 | NOTCH2;CREBBP;CCND1;MAML1;AGO4;AGO1;AGO2;EP300;MAMLD1 |
| Downstream signal transduction Homo sapiens R-HSA-186763 | 3.57E-05 | PHLPP2;AHCYL1;PHLPP1;IRS1;ITPR3;ADCY7;DUSP16;EGFR;CRKL;SPRED2;PSMD5;PRKAR2A;PIP4K2A;MAPK1;SPTBN1;JAK1;PDPK1;PRKCE;FN1;PRKCA;MTOR;GDNF;AGO4;AGO1; AGO2;VCL |
| YAP1- and WWTR1 (TAZ)-stimulated gene expression Homo sapiens R-HSA-2032785 | 2.62E-05 | HELZ2;CREBBP;NCOA6;TBL1X;PPARA;RUNX2;HIPK2 |
| Signaling by FGFR3 Homo sapiens R-HSA-5654741 | 2.38E-05 | PHLPP2;AHCYL1;PHLPP1;IRS1;ITPR3;CBL;ADCY7;DUSP16;EGFR;SPRED2;PSMD5;PRKAR2A;PIP4K2A;MAPK1;SPTBN1;JAK1;PDPK1;PRKCE;FN1;PRKCA;MTOR;GDNF;AGO4;AGO1; AGO2;VCL |
| EGFR interacts with phospholipase C-gamma Homo sapiens R-HSA-212718 | 7.86E-05 | AHCYL1;PRKAR2A;PRKCE;ITPR3;PRKCA;ADCY7;EGFR |
| Signaling by FGFR2 Homo sapiens R-HSA-5654738 | 3.55E-05 | PHLPP2;AHCYL1;PHLPP1;IRS1;ITPR3;CBL;ADCY7;DUSP16;EGFR;SPRED2;PSMD5;PRKAR2A;PIP4K2A;MAPK1;SPTBN1;JAK1;RBFOX2;PDPK1;PRKCE;FN1;PRKCA;MTOR;GDNF;AGO4; AGO1;AGO2;VCL |
| Regulation of lipid metabolism by Peroxisome proliferator-activated receptor alpha (PPARalpha) Homo sapiens R-HSA-400206 | 1.82E-05 | CREBBP;NFYA;NCOA6;NCOA3;MED13L;HELZ2;MED12;SP1;SIN3A;EP300;TBL1X;RGL1;PPARA;PPARGC1B |
| Signaling by FGFR4 Homo sapiens R-HSA-5654743 | 2.26E-05 | PHLPP2;AHCYL1;PHLPP1;IRS1;ITPR3;CBL;ADCY7;DUSP16;EGFR;SPRED2;PSMD5;PRKAR2A;PIP4K2A;MAPK1;SPTBN1;JAK1;PDPK1;PRKCE;FN1;PRKCA;MTOR;GDNF;AGO4;AGO1; AGO2;VCL |
| Developmental Biology Homo sapiens R-HSA-1266738 | 9.74E-05 | SEMA5A;KMT2D;TRIO;PAXIP1;IRS1;EPAS1;LAMA1;LAMC1;ACVR1B;DUSP16;EGFR;ARHGAP35;MED12;HELZ2;EFNB2;SPRED2;PSMD5;ABL2;PLXNA1;EP300;MAPK1;TBL1X;NEO1; SPTBN1;CLASP1;JAK1;CDON;CREBBP;SMAD3;CSNK2A1;TSC22D1;NCOA6;NCOA3;FN1;MED13L;TIAM1;GDNF;SCN8A;NUMB;KCNQ3;COL6A3;PPARA;ARHGEF7;MET;VCL |
| DAP12 signaling Homo sapiens R-HSA-2424491 | 1.08E-04 | PHLPP2;AHCYL1;PHLPP1;IRS1;ITPR3;ADCY7;DUSP16;EGFR;SPRED2;PSMD5;PRKAR2A;PIP4K2A;MAPK1;SPTBN1;JAK1;PDPK1;PRKCE;FN1;PRKCA;MTOR;GDNF;AGO4;AGO1;AGO2;VCL |
| Signaling by NOTCH Homo sapiens R-HSA-157118 | 1.32E-04 | NOTCH2;CREBBP;MAML1;NEURL1B;ADAM17;CCND1;AGO4;AGO1;AGO2;NUMB;EP300;TBL1X;MAMLD1 |
| RORA activates gene expression Homo sapiens R-HSA-1368082 | 1.65E-04 | HELZ2;CREBBP;NCOA6;EP300;TBL1X;PPARA |
| Circadian Clock Homo sapiens R-HSA-400253 | 1.55E-04 | HELZ2;PER2;RAI1;CREBBP;NCOA6;EP300;TBL1X;BTRC;PPARA |
| NGF signalling via TRKA from the plasma membrane Homo sapiens R-HSA-187037 | 1.62E-04 | PHLPP2;AHCYL1;PHLPP1;IRS1;ITPR3;ADCY7;DUSP16;EGFR;CRKL;SPRED2;PSMD5;PRKAR2A;PIP4K2A;MAPK1;SPTBN1;JAK1;PDPK1;PRKCE;FN1;PRKCA;MTOR;GDNF;AGO4;AGO1; AGO2;VCL |
| Nuclear Envelope Breakdown Homo sapiens R-HSA-2980766 | 1.84E-04 | NUP214;NEK9;NUP205;NUP188;NUP155;PRKCA;NUP98;LPIN2 |

## Slide 12
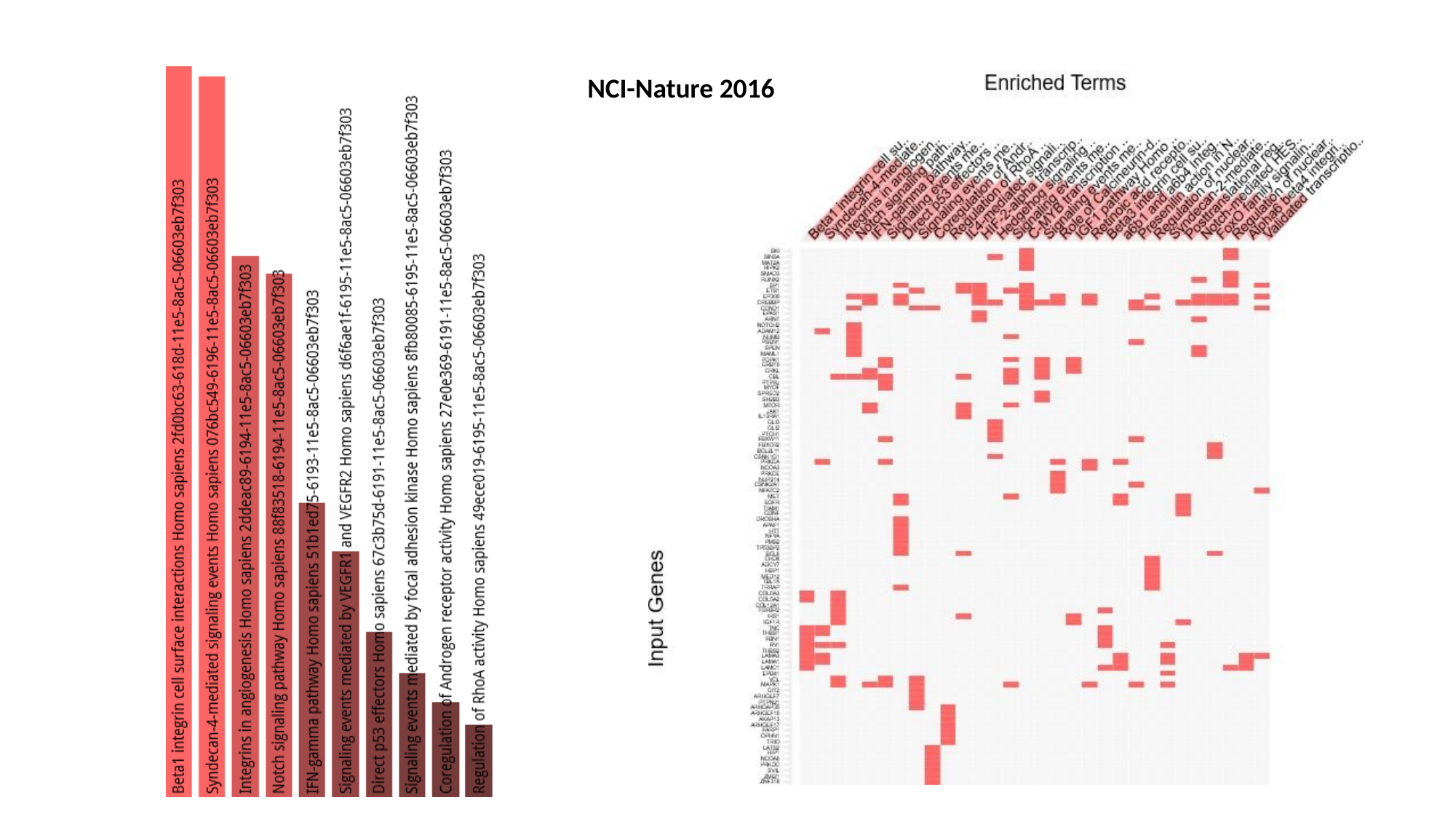

NCI-Nature 2016

## Slide 13
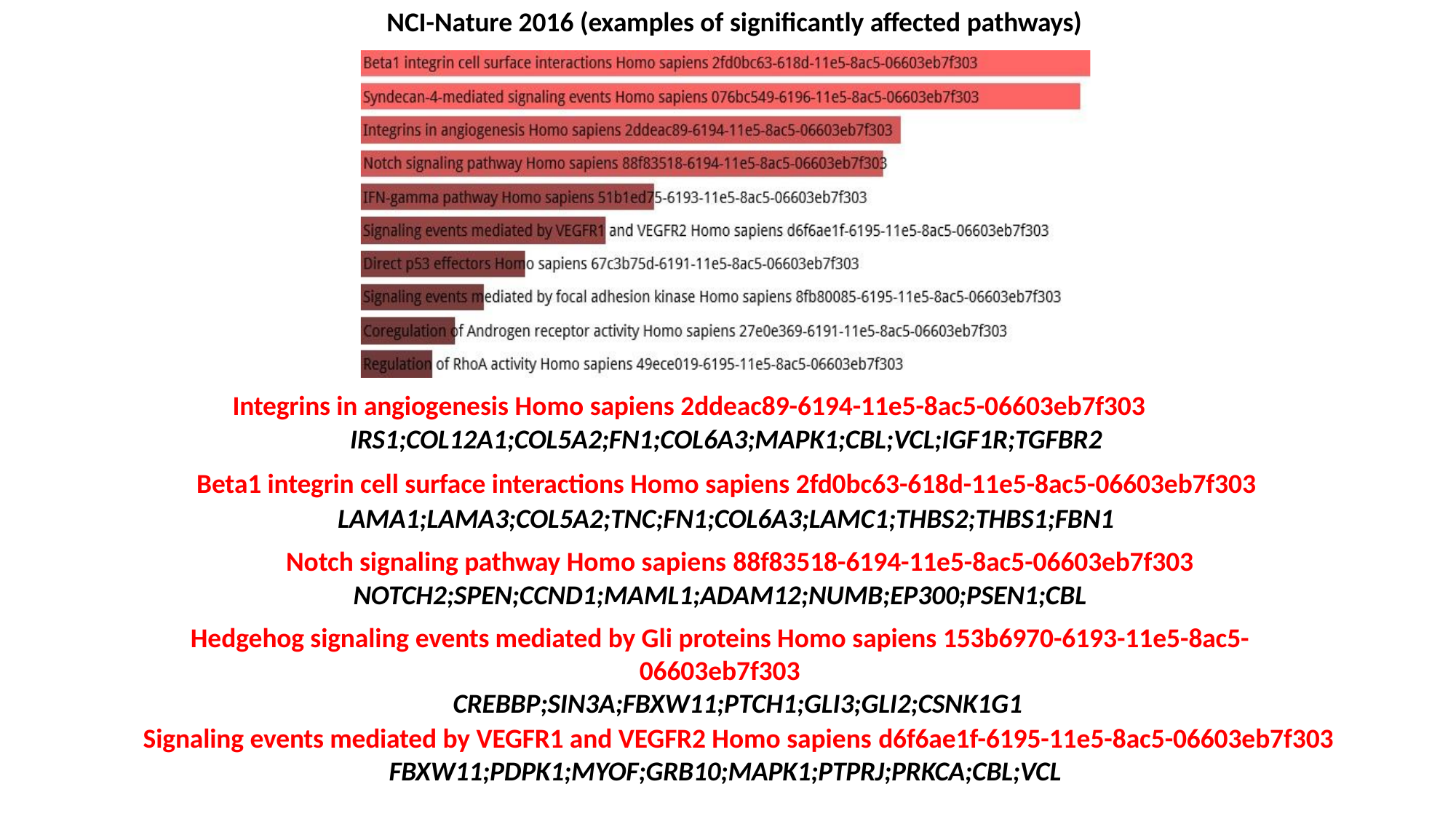

# NCI-Nature 2016 (examples of significantly affected pathways)
Integrins in angiogenesis Homo sapiens 2ddeac89-6194-11e5-8ac5-06603eb7f303
IRS1;COL12A1;COL5A2;FN1;COL6A3;MAPK1;CBL;VCL;IGF1R;TGFBR2
Beta1 integrin cell surface interactions Homo sapiens 2fd0bc63-618d-11e5-8ac5-06603eb7f303
LAMA1;LAMA3;COL5A2;TNC;FN1;COL6A3;LAMC1;THBS2;THBS1;FBN1
Notch signaling pathway Homo sapiens 88f83518-6194-11e5-8ac5-06603eb7f303
NOTCH2;SPEN;CCND1;MAML1;ADAM12;NUMB;EP300;PSEN1;CBL
Hedgehog signaling events mediated by Gli proteins Homo sapiens 153b6970-6193-11e5-8ac5-06603eb7f303
CREBBP;SIN3A;FBXW11;PTCH1;GLI3;GLI2;CSNK1G1
Signaling events mediated by VEGFR1 and VEGFR2 Homo sapiens d6f6ae1f-6195-11e5-8ac5-06603eb7f303
FBXW11;PDPK1;MYOF;GRB10;MAPK1;PTPRJ;PRKCA;CBL;VCL

## Slide 14
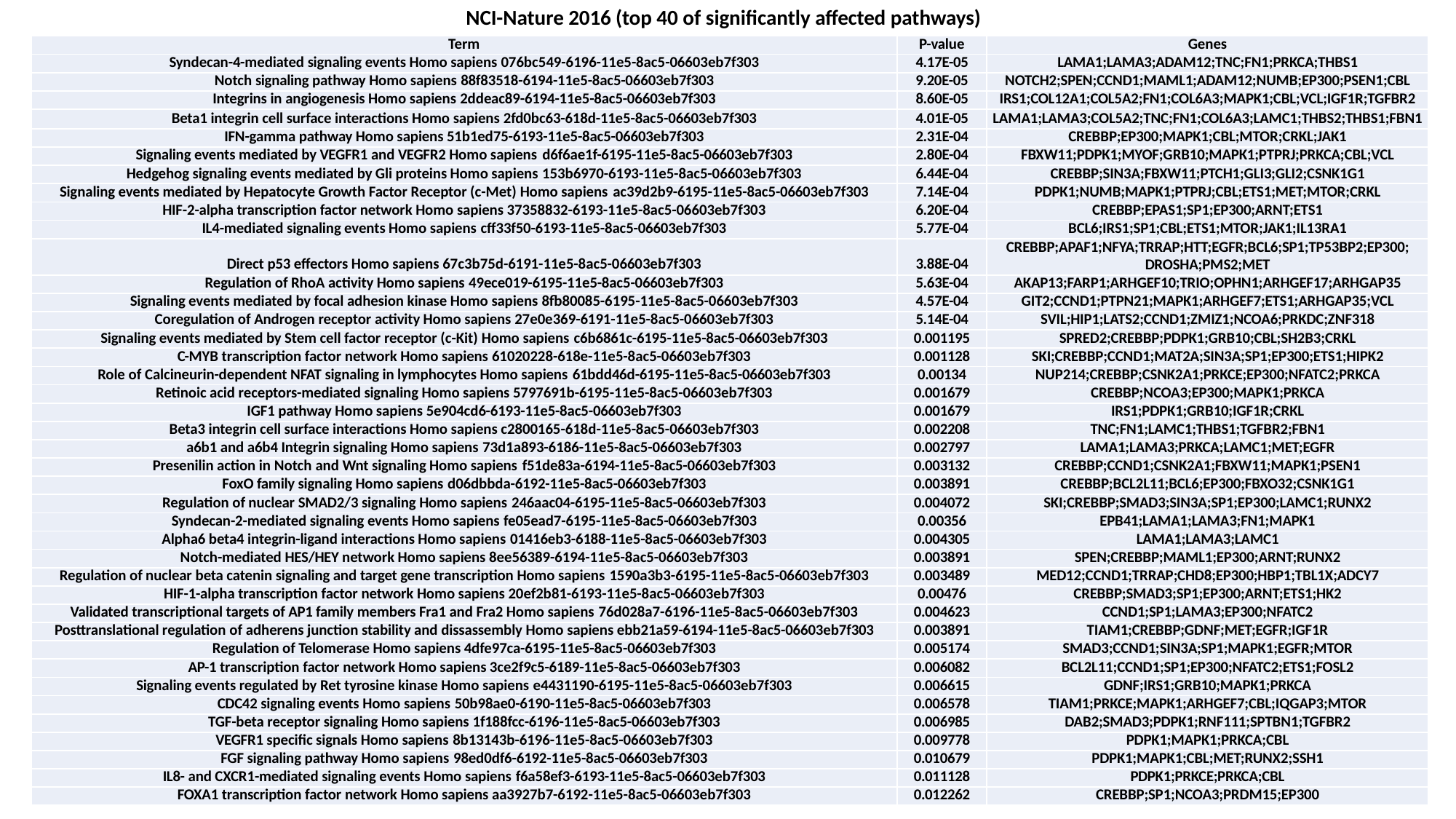

NCI-Nature 2016 (top 40 of significantly affected pathways)
| Term | P-value | Genes |
| --- | --- | --- |
| Syndecan-4-mediated signaling events Homo sapiens 076bc549-6196-11e5-8ac5-06603eb7f303 | 4.17E-05 | LAMA1;LAMA3;ADAM12;TNC;FN1;PRKCA;THBS1 |
| Notch signaling pathway Homo sapiens 88f83518-6194-11e5-8ac5-06603eb7f303 | 9.20E-05 | NOTCH2;SPEN;CCND1;MAML1;ADAM12;NUMB;EP300;PSEN1;CBL |
| Integrins in angiogenesis Homo sapiens 2ddeac89-6194-11e5-8ac5-06603eb7f303 | 8.60E-05 | IRS1;COL12A1;COL5A2;FN1;COL6A3;MAPK1;CBL;VCL;IGF1R;TGFBR2 |
| Beta1 integrin cell surface interactions Homo sapiens 2fd0bc63-618d-11e5-8ac5-06603eb7f303 | 4.01E-05 | LAMA1;LAMA3;COL5A2;TNC;FN1;COL6A3;LAMC1;THBS2;THBS1;FBN1 |
| IFN-gamma pathway Homo sapiens 51b1ed75-6193-11e5-8ac5-06603eb7f303 | 2.31E-04 | CREBBP;EP300;MAPK1;CBL;MTOR;CRKL;JAK1 |
| Signaling events mediated by VEGFR1 and VEGFR2 Homo sapiens d6f6ae1f-6195-11e5-8ac5-06603eb7f303 | 2.80E-04 | FBXW11;PDPK1;MYOF;GRB10;MAPK1;PTPRJ;PRKCA;CBL;VCL |
| Hedgehog signaling events mediated by Gli proteins Homo sapiens 153b6970-6193-11e5-8ac5-06603eb7f303 | 6.44E-04 | CREBBP;SIN3A;FBXW11;PTCH1;GLI3;GLI2;CSNK1G1 |
| Signaling events mediated by Hepatocyte Growth Factor Receptor (c-Met) Homo sapiens ac39d2b9-6195-11e5-8ac5-06603eb7f303 | 7.14E-04 | PDPK1;NUMB;MAPK1;PTPRJ;CBL;ETS1;MET;MTOR;CRKL |
| HIF-2-alpha transcription factor network Homo sapiens 37358832-6193-11e5-8ac5-06603eb7f303 | 6.20E-04 | CREBBP;EPAS1;SP1;EP300;ARNT;ETS1 |
| IL4-mediated signaling events Homo sapiens cff33f50-6193-11e5-8ac5-06603eb7f303 | 5.77E-04 | BCL6;IRS1;SP1;CBL;ETS1;MTOR;JAK1;IL13RA1 |
| Direct p53 effectors Homo sapiens 67c3b75d-6191-11e5-8ac5-06603eb7f303 | 3.88E-04 | CREBBP;APAF1;NFYA;TRRAP;HTT;EGFR;BCL6;SP1;TP53BP2;EP300; DROSHA;PMS2;MET |
| Regulation of RhoA activity Homo sapiens 49ece019-6195-11e5-8ac5-06603eb7f303 | 5.63E-04 | AKAP13;FARP1;ARHGEF10;TRIO;OPHN1;ARHGEF17;ARHGAP35 |
| Signaling events mediated by focal adhesion kinase Homo sapiens 8fb80085-6195-11e5-8ac5-06603eb7f303 | 4.57E-04 | GIT2;CCND1;PTPN21;MAPK1;ARHGEF7;ETS1;ARHGAP35;VCL |
| Coregulation of Androgen receptor activity Homo sapiens 27e0e369-6191-11e5-8ac5-06603eb7f303 | 5.14E-04 | SVIL;HIP1;LATS2;CCND1;ZMIZ1;NCOA6;PRKDC;ZNF318 |
| Signaling events mediated by Stem cell factor receptor (c-Kit) Homo sapiens c6b6861c-6195-11e5-8ac5-06603eb7f303 | 0.001195 | SPRED2;CREBBP;PDPK1;GRB10;CBL;SH2B3;CRKL |
| C-MYB transcription factor network Homo sapiens 61020228-618e-11e5-8ac5-06603eb7f303 | 0.001128 | SKI;CREBBP;CCND1;MAT2A;SIN3A;SP1;EP300;ETS1;HIPK2 |
| Role of Calcineurin-dependent NFAT signaling in lymphocytes Homo sapiens 61bdd46d-6195-11e5-8ac5-06603eb7f303 | 0.00134 | NUP214;CREBBP;CSNK2A1;PRKCE;EP300;NFATC2;PRKCA |
| Retinoic acid receptors-mediated signaling Homo sapiens 5797691b-6195-11e5-8ac5-06603eb7f303 | 0.001679 | CREBBP;NCOA3;EP300;MAPK1;PRKCA |
| IGF1 pathway Homo sapiens 5e904cd6-6193-11e5-8ac5-06603eb7f303 | 0.001679 | IRS1;PDPK1;GRB10;IGF1R;CRKL |
| Beta3 integrin cell surface interactions Homo sapiens c2800165-618d-11e5-8ac5-06603eb7f303 | 0.002208 | TNC;FN1;LAMC1;THBS1;TGFBR2;FBN1 |
| a6b1 and a6b4 Integrin signaling Homo sapiens 73d1a893-6186-11e5-8ac5-06603eb7f303 | 0.002797 | LAMA1;LAMA3;PRKCA;LAMC1;MET;EGFR |
| Presenilin action in Notch and Wnt signaling Homo sapiens f51de83a-6194-11e5-8ac5-06603eb7f303 | 0.003132 | CREBBP;CCND1;CSNK2A1;FBXW11;MAPK1;PSEN1 |
| FoxO family signaling Homo sapiens d06dbbda-6192-11e5-8ac5-06603eb7f303 | 0.003891 | CREBBP;BCL2L11;BCL6;EP300;FBXO32;CSNK1G1 |
| Regulation of nuclear SMAD2/3 signaling Homo sapiens 246aac04-6195-11e5-8ac5-06603eb7f303 | 0.004072 | SKI;CREBBP;SMAD3;SIN3A;SP1;EP300;LAMC1;RUNX2 |
| Syndecan-2-mediated signaling events Homo sapiens fe05ead7-6195-11e5-8ac5-06603eb7f303 | 0.00356 | EPB41;LAMA1;LAMA3;FN1;MAPK1 |
| Alpha6 beta4 integrin-ligand interactions Homo sapiens 01416eb3-6188-11e5-8ac5-06603eb7f303 | 0.004305 | LAMA1;LAMA3;LAMC1 |
| Notch-mediated HES/HEY network Homo sapiens 8ee56389-6194-11e5-8ac5-06603eb7f303 | 0.003891 | SPEN;CREBBP;MAML1;EP300;ARNT;RUNX2 |
| Regulation of nuclear beta catenin signaling and target gene transcription Homo sapiens 1590a3b3-6195-11e5-8ac5-06603eb7f303 | 0.003489 | MED12;CCND1;TRRAP;CHD8;EP300;HBP1;TBL1X;ADCY7 |
| HIF-1-alpha transcription factor network Homo sapiens 20ef2b81-6193-11e5-8ac5-06603eb7f303 | 0.00476 | CREBBP;SMAD3;SP1;EP300;ARNT;ETS1;HK2 |
| Validated transcriptional targets of AP1 family members Fra1 and Fra2 Homo sapiens 76d028a7-6196-11e5-8ac5-06603eb7f303 | 0.004623 | CCND1;SP1;LAMA3;EP300;NFATC2 |
| Posttranslational regulation of adherens junction stability and dissassembly Homo sapiens ebb21a59-6194-11e5-8ac5-06603eb7f303 | 0.003891 | TIAM1;CREBBP;GDNF;MET;EGFR;IGF1R |
| Regulation of Telomerase Homo sapiens 4dfe97ca-6195-11e5-8ac5-06603eb7f303 | 0.005174 | SMAD3;CCND1;SIN3A;SP1;MAPK1;EGFR;MTOR |
| AP-1 transcription factor network Homo sapiens 3ce2f9c5-6189-11e5-8ac5-06603eb7f303 | 0.006082 | BCL2L11;CCND1;SP1;EP300;NFATC2;ETS1;FOSL2 |
| Signaling events regulated by Ret tyrosine kinase Homo sapiens e4431190-6195-11e5-8ac5-06603eb7f303 | 0.006615 | GDNF;IRS1;GRB10;MAPK1;PRKCA |
| CDC42 signaling events Homo sapiens 50b98ae0-6190-11e5-8ac5-06603eb7f303 | 0.006578 | TIAM1;PRKCE;MAPK1;ARHGEF7;CBL;IQGAP3;MTOR |
| TGF-beta receptor signaling Homo sapiens 1f188fcc-6196-11e5-8ac5-06603eb7f303 | 0.006985 | DAB2;SMAD3;PDPK1;RNF111;SPTBN1;TGFBR2 |
| VEGFR1 specific signals Homo sapiens 8b13143b-6196-11e5-8ac5-06603eb7f303 | 0.009778 | PDPK1;MAPK1;PRKCA;CBL |
| FGF signaling pathway Homo sapiens 98ed0df6-6192-11e5-8ac5-06603eb7f303 | 0.010679 | PDPK1;MAPK1;CBL;MET;RUNX2;SSH1 |
| IL8- and CXCR1-mediated signaling events Homo sapiens f6a58ef3-6193-11e5-8ac5-06603eb7f303 | 0.011128 | PDPK1;PRKCE;PRKCA;CBL |
| FOXA1 transcription factor network Homo sapiens aa3927b7-6192-11e5-8ac5-06603eb7f303 | 0.012262 | CREBBP;SP1;NCOA3;PRDM15;EP300 |

## Slide 15
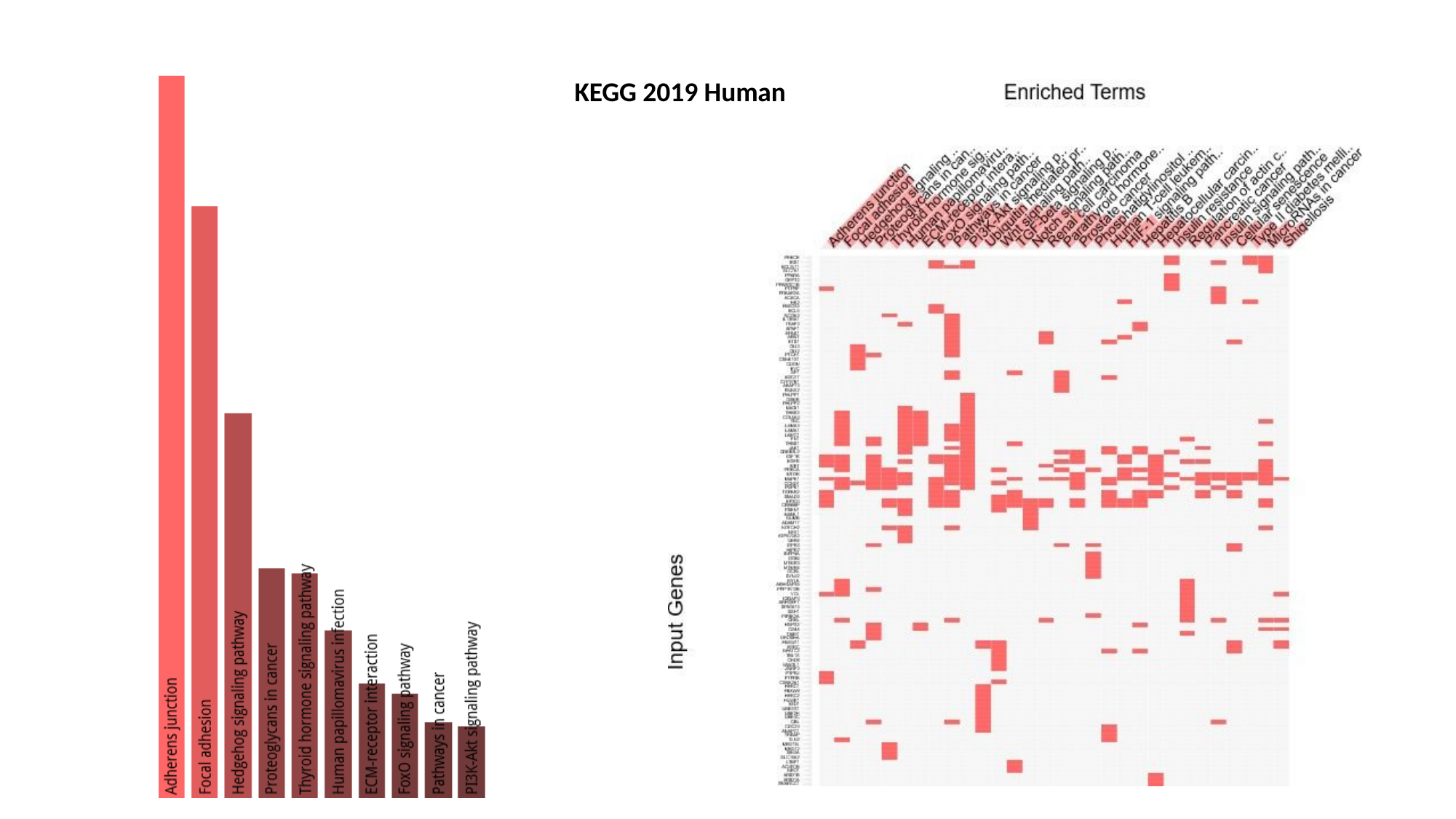

KEGG 2019 Human

## Slide 16
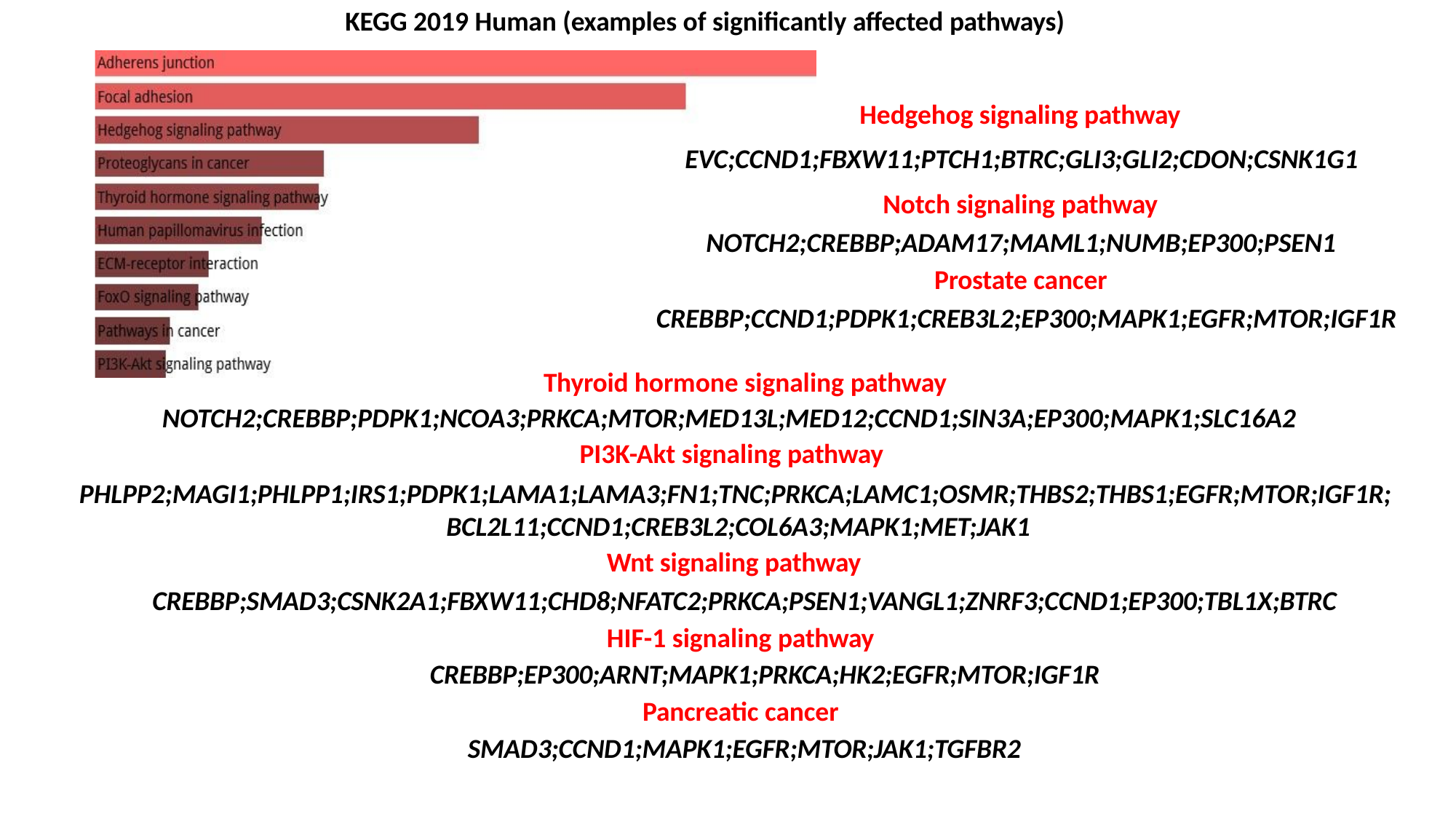

# KEGG 2019 Human (examples of significantly affected pathways)
Hedgehog signaling pathway
EVC;CCND1;FBXW11;PTCH1;BTRC;GLI3;GLI2;CDON;CSNK1G1
Notch signaling pathway
NOTCH2;CREBBP;ADAM17;MAML1;NUMB;EP300;PSEN1
Prostate cancer
CREBBP;CCND1;PDPK1;CREB3L2;EP300;MAPK1;EGFR;MTOR;IGF1R
Thyroid hormone signaling pathway
NOTCH2;CREBBP;PDPK1;NCOA3;PRKCA;MTOR;MED13L;MED12;CCND1;SIN3A;EP300;MAPK1;SLC16A2
PI3K-Akt signaling pathway
PHLPP2;MAGI1;PHLPP1;IRS1;PDPK1;LAMA1;LAMA3;FN1;TNC;PRKCA;LAMC1;OSMR;THBS2;THBS1;EGFR;MTOR;IGF1R; BCL2L11;CCND1;CREB3L2;COL6A3;MAPK1;MET;JAK1
Wnt signaling pathway
CREBBP;SMAD3;CSNK2A1;FBXW11;CHD8;NFATC2;PRKCA;PSEN1;VANGL1;ZNRF3;CCND1;EP300;TBL1X;BTRC
HIF-1 signaling pathway
CREBBP;EP300;ARNT;MAPK1;PRKCA;HK2;EGFR;MTOR;IGF1R
Pancreatic cancer
SMAD3;CCND1;MAPK1;EGFR;MTOR;JAK1;TGFBR2

## Slide 17
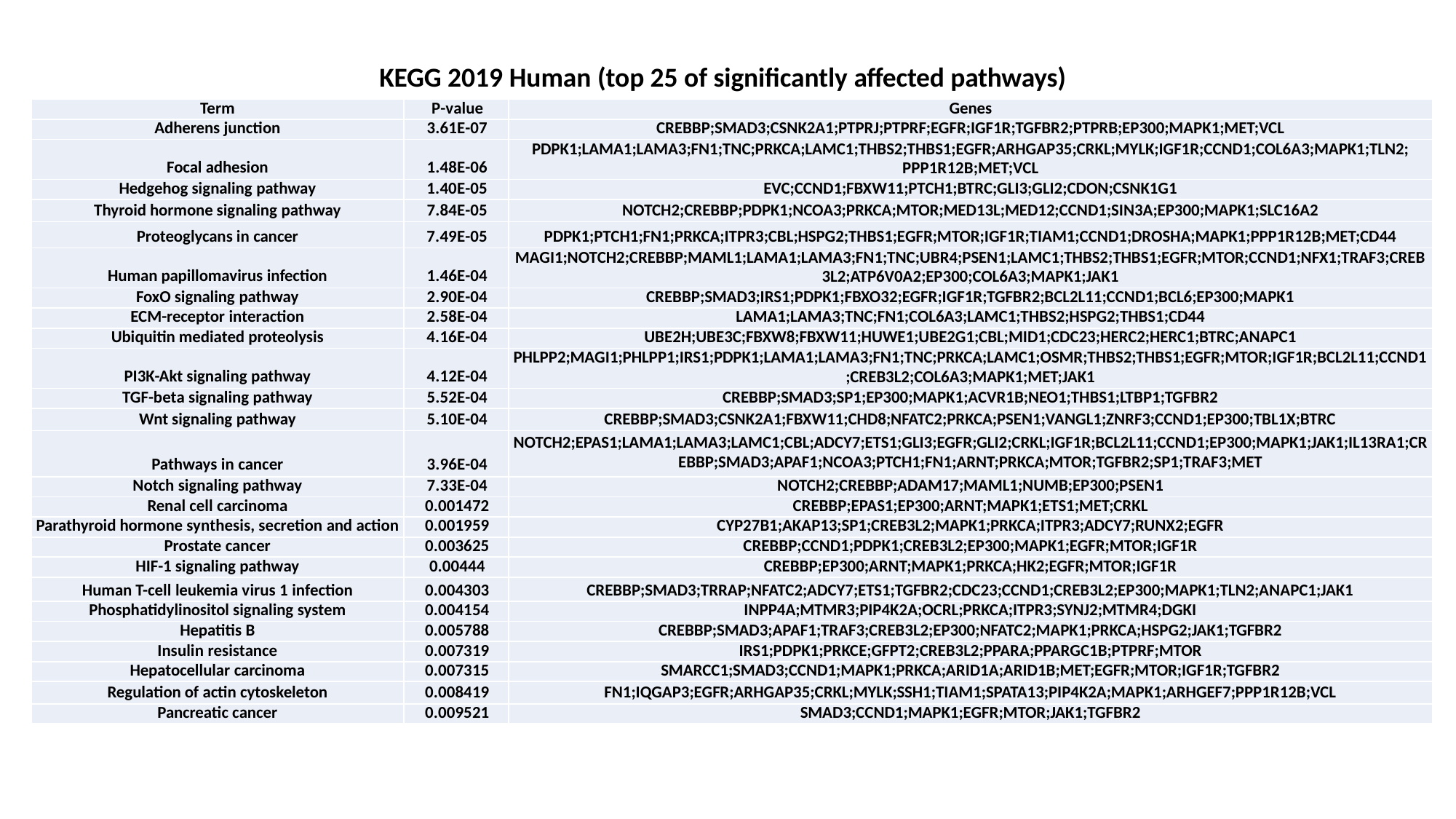

# KEGG 2019 Human (top 25 of significantly affected pathways)
| Term | P-value | Genes |
| --- | --- | --- |
| Adherens junction | 3.61E-07 | CREBBP;SMAD3;CSNK2A1;PTPRJ;PTPRF;EGFR;IGF1R;TGFBR2;PTPRB;EP300;MAPK1;MET;VCL |
| Focal adhesion | 1.48E-06 | PDPK1;LAMA1;LAMA3;FN1;TNC;PRKCA;LAMC1;THBS2;THBS1;EGFR;ARHGAP35;CRKL;MYLK;IGF1R;CCND1;COL6A3;MAPK1;TLN2; PPP1R12B;MET;VCL |
| Hedgehog signaling pathway | 1.40E-05 | EVC;CCND1;FBXW11;PTCH1;BTRC;GLI3;GLI2;CDON;CSNK1G1 |
| Thyroid hormone signaling pathway | 7.84E-05 | NOTCH2;CREBBP;PDPK1;NCOA3;PRKCA;MTOR;MED13L;MED12;CCND1;SIN3A;EP300;MAPK1;SLC16A2 |
| Proteoglycans in cancer | 7.49E-05 | PDPK1;PTCH1;FN1;PRKCA;ITPR3;CBL;HSPG2;THBS1;EGFR;MTOR;IGF1R;TIAM1;CCND1;DROSHA;MAPK1;PPP1R12B;MET;CD44 |
| Human papillomavirus infection | 1.46E-04 | MAGI1;NOTCH2;CREBBP;MAML1;LAMA1;LAMA3;FN1;TNC;UBR4;PSEN1;LAMC1;THBS2;THBS1;EGFR;MTOR;CCND1;NFX1;TRAF3;CREB 3L2;ATP6V0A2;EP300;COL6A3;MAPK1;JAK1 |
| FoxO signaling pathway | 2.90E-04 | CREBBP;SMAD3;IRS1;PDPK1;FBXO32;EGFR;IGF1R;TGFBR2;BCL2L11;CCND1;BCL6;EP300;MAPK1 |
| ECM-receptor interaction | 2.58E-04 | LAMA1;LAMA3;TNC;FN1;COL6A3;LAMC1;THBS2;HSPG2;THBS1;CD44 |
| Ubiquitin mediated proteolysis | 4.16E-04 | UBE2H;UBE3C;FBXW8;FBXW11;HUWE1;UBE2G1;CBL;MID1;CDC23;HERC2;HERC1;BTRC;ANAPC1 |
| PI3K-Akt signaling pathway | 4.12E-04 | PHLPP2;MAGI1;PHLPP1;IRS1;PDPK1;LAMA1;LAMA3;FN1;TNC;PRKCA;LAMC1;OSMR;THBS2;THBS1;EGFR;MTOR;IGF1R;BCL2L11;CCND1 ;CREB3L2;COL6A3;MAPK1;MET;JAK1 |
| TGF-beta signaling pathway | 5.52E-04 | CREBBP;SMAD3;SP1;EP300;MAPK1;ACVR1B;NEO1;THBS1;LTBP1;TGFBR2 |
| Wnt signaling pathway | 5.10E-04 | CREBBP;SMAD3;CSNK2A1;FBXW11;CHD8;NFATC2;PRKCA;PSEN1;VANGL1;ZNRF3;CCND1;EP300;TBL1X;BTRC |
| Pathways in cancer | 3.96E-04 | NOTCH2;EPAS1;LAMA1;LAMA3;LAMC1;CBL;ADCY7;ETS1;GLI3;EGFR;GLI2;CRKL;IGF1R;BCL2L11;CCND1;EP300;MAPK1;JAK1;IL13RA1;CR EBBP;SMAD3;APAF1;NCOA3;PTCH1;FN1;ARNT;PRKCA;MTOR;TGFBR2;SP1;TRAF3;MET |
| Notch signaling pathway | 7.33E-04 | NOTCH2;CREBBP;ADAM17;MAML1;NUMB;EP300;PSEN1 |
| Renal cell carcinoma | 0.001472 | CREBBP;EPAS1;EP300;ARNT;MAPK1;ETS1;MET;CRKL |
| Parathyroid hormone synthesis, secretion and action | 0.001959 | CYP27B1;AKAP13;SP1;CREB3L2;MAPK1;PRKCA;ITPR3;ADCY7;RUNX2;EGFR |
| Prostate cancer | 0.003625 | CREBBP;CCND1;PDPK1;CREB3L2;EP300;MAPK1;EGFR;MTOR;IGF1R |
| HIF-1 signaling pathway | 0.00444 | CREBBP;EP300;ARNT;MAPK1;PRKCA;HK2;EGFR;MTOR;IGF1R |
| Human T-cell leukemia virus 1 infection | 0.004303 | CREBBP;SMAD3;TRRAP;NFATC2;ADCY7;ETS1;TGFBR2;CDC23;CCND1;CREB3L2;EP300;MAPK1;TLN2;ANAPC1;JAK1 |
| Phosphatidylinositol signaling system | 0.004154 | INPP4A;MTMR3;PIP4K2A;OCRL;PRKCA;ITPR3;SYNJ2;MTMR4;DGKI |
| Hepatitis B | 0.005788 | CREBBP;SMAD3;APAF1;TRAF3;CREB3L2;EP300;NFATC2;MAPK1;PRKCA;HSPG2;JAK1;TGFBR2 |
| Insulin resistance | 0.007319 | IRS1;PDPK1;PRKCE;GFPT2;CREB3L2;PPARA;PPARGC1B;PTPRF;MTOR |
| Hepatocellular carcinoma | 0.007315 | SMARCC1;SMAD3;CCND1;MAPK1;PRKCA;ARID1A;ARID1B;MET;EGFR;MTOR;IGF1R;TGFBR2 |
| Regulation of actin cytoskeleton | 0.008419 | FN1;IQGAP3;EGFR;ARHGAP35;CRKL;MYLK;SSH1;TIAM1;SPATA13;PIP4K2A;MAPK1;ARHGEF7;PPP1R12B;VCL |
| Pancreatic cancer | 0.009521 | SMAD3;CCND1;MAPK1;EGFR;MTOR;JAK1;TGFBR2 |

## Slide 18
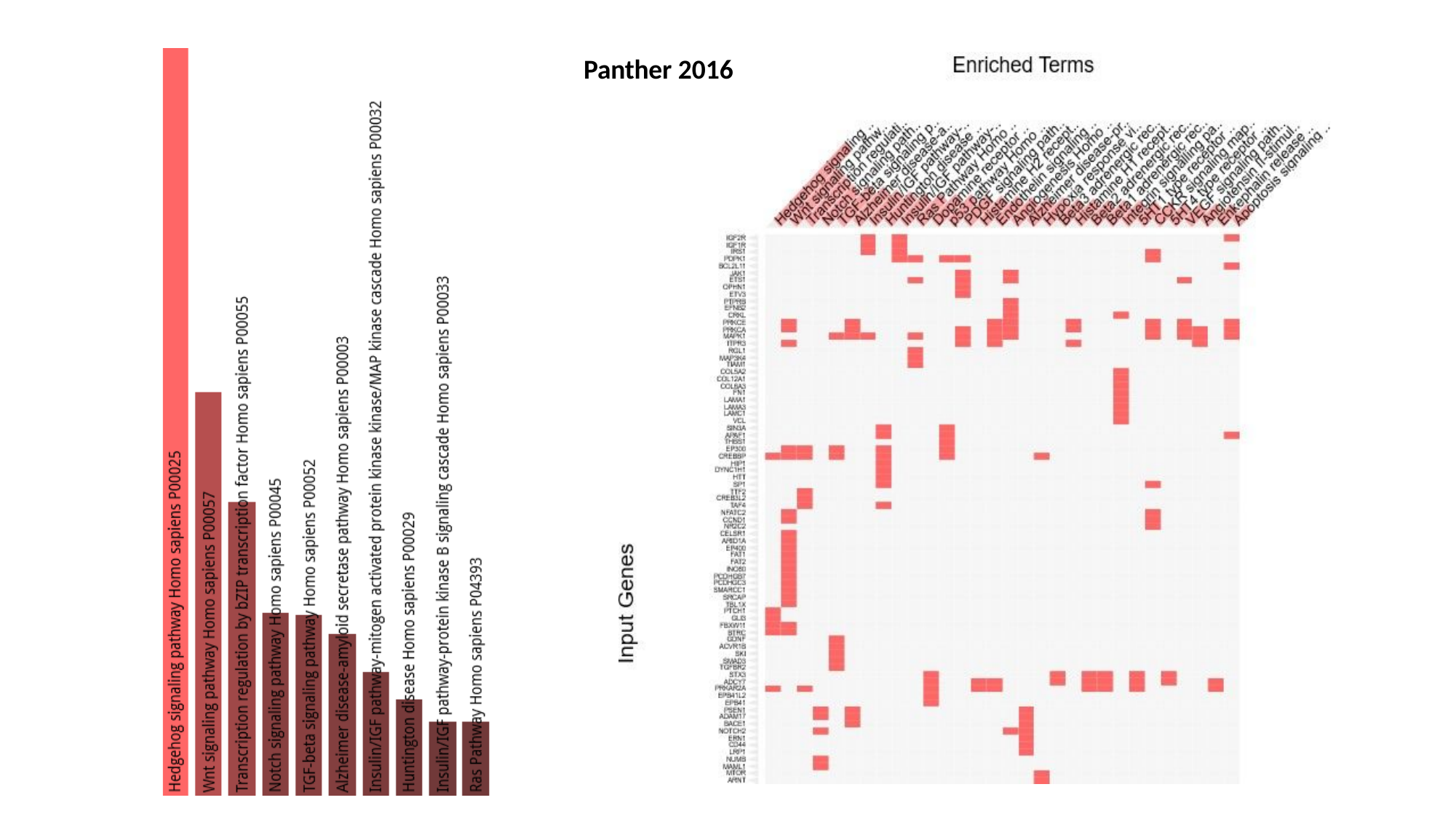

Panther 2016

## Slide 19
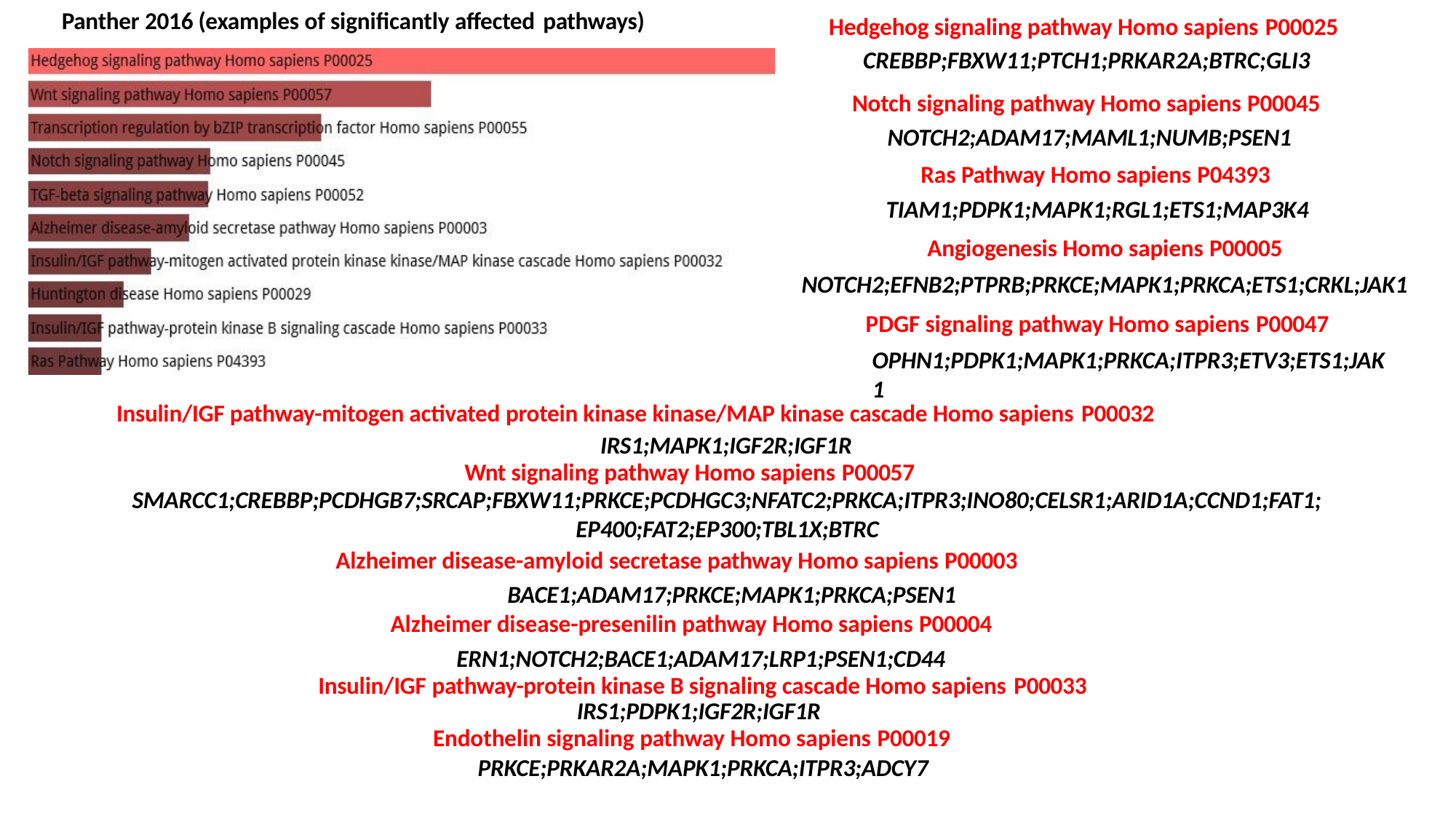

Panther 2016 (examples of significantly affected pathways)
Hedgehog signaling pathway Homo sapiens P00025
CREBBP;FBXW11;PTCH1;PRKAR2A;BTRC;GLI3
Notch signaling pathway Homo sapiens P00045
NOTCH2;ADAM17;MAML1;NUMB;PSEN1
Ras Pathway Homo sapiens P04393
TIAM1;PDPK1;MAPK1;RGL1;ETS1;MAP3K4
Angiogenesis Homo sapiens P00005
NOTCH2;EFNB2;PTPRB;PRKCE;MAPK1;PRKCA;ETS1;CRKL;JAK1
PDGF signaling pathway Homo sapiens P00047
OPHN1;PDPK1;MAPK1;PRKCA;ITPR3;ETV3;ETS1;JAK1
Insulin/IGF pathway-mitogen activated protein kinase kinase/MAP kinase cascade Homo sapiens P00032
IRS1;MAPK1;IGF2R;IGF1R
Wnt signaling pathway Homo sapiens P00057
SMARCC1;CREBBP;PCDHGB7;SRCAP;FBXW11;PRKCE;PCDHGC3;NFATC2;PRKCA;ITPR3;INO80;CELSR1;ARID1A;CCND1;FAT1;
EP400;FAT2;EP300;TBL1X;BTRC
Alzheimer disease-amyloid secretase pathway Homo sapiens P00003
BACE1;ADAM17;PRKCE;MAPK1;PRKCA;PSEN1
Alzheimer disease-presenilin pathway Homo sapiens P00004
ERN1;NOTCH2;BACE1;ADAM17;LRP1;PSEN1;CD44
Insulin/IGF pathway-protein kinase B signaling cascade Homo sapiens P00033
IRS1;PDPK1;IGF2R;IGF1R
Endothelin signaling pathway Homo sapiens P00019
PRKCE;PRKAR2A;MAPK1;PRKCA;ITPR3;ADCY7

## Slide 20
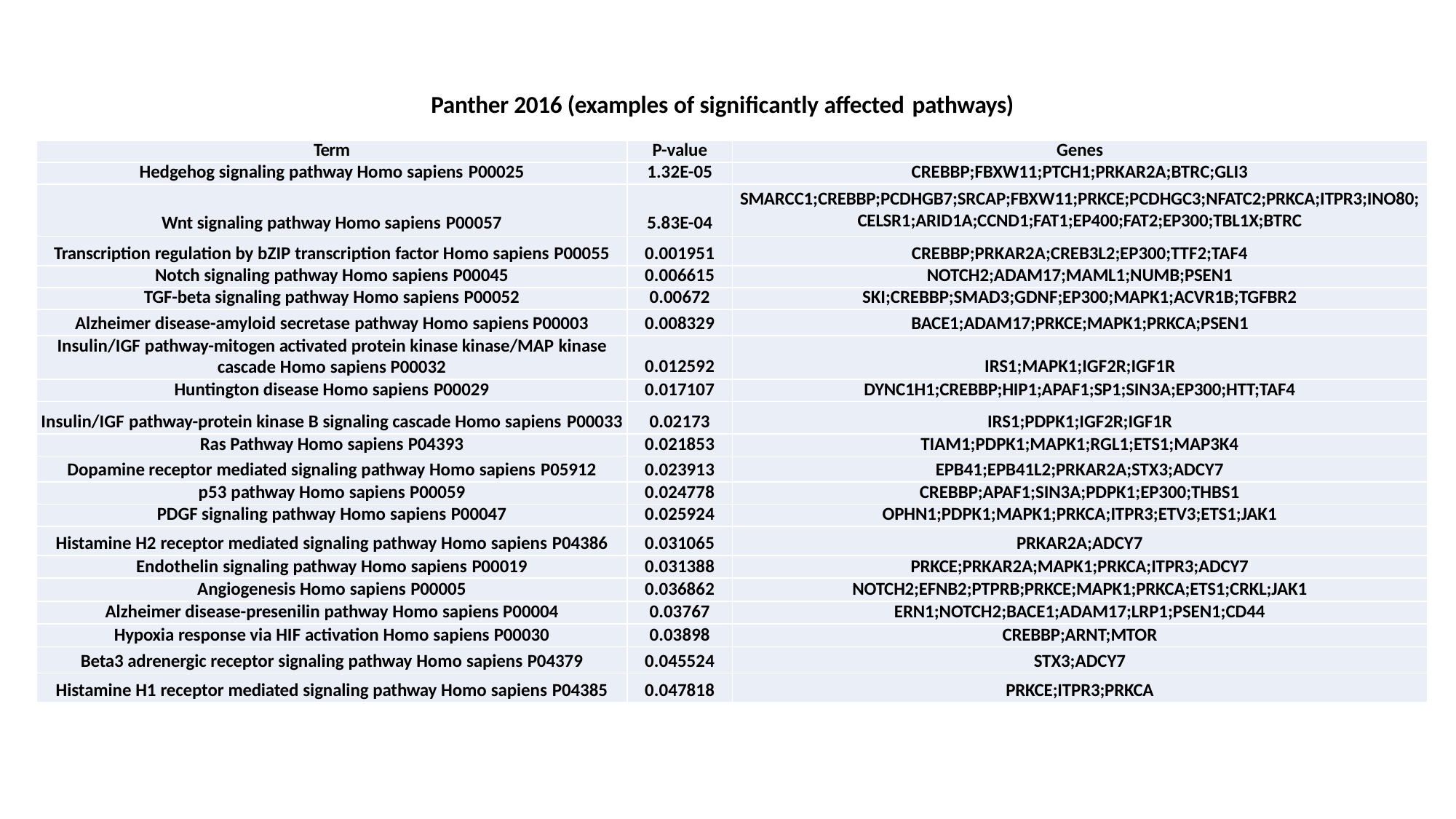

Panther 2016 (examples of significantly affected pathways)
| Term | P-value | Genes |
| --- | --- | --- |
| Hedgehog signaling pathway Homo sapiens P00025 | 1.32E-05 | CREBBP;FBXW11;PTCH1;PRKAR2A;BTRC;GLI3 |
| Wnt signaling pathway Homo sapiens P00057 | 5.83E-04 | SMARCC1;CREBBP;PCDHGB7;SRCAP;FBXW11;PRKCE;PCDHGC3;NFATC2;PRKCA;ITPR3;INO80; CELSR1;ARID1A;CCND1;FAT1;EP400;FAT2;EP300;TBL1X;BTRC |
| Transcription regulation by bZIP transcription factor Homo sapiens P00055 | 0.001951 | CREBBP;PRKAR2A;CREB3L2;EP300;TTF2;TAF4 |
| Notch signaling pathway Homo sapiens P00045 | 0.006615 | NOTCH2;ADAM17;MAML1;NUMB;PSEN1 |
| TGF-beta signaling pathway Homo sapiens P00052 | 0.00672 | SKI;CREBBP;SMAD3;GDNF;EP300;MAPK1;ACVR1B;TGFBR2 |
| Alzheimer disease-amyloid secretase pathway Homo sapiens P00003 | 0.008329 | BACE1;ADAM17;PRKCE;MAPK1;PRKCA;PSEN1 |
| Insulin/IGF pathway-mitogen activated protein kinase kinase/MAP kinase cascade Homo sapiens P00032 | 0.012592 | IRS1;MAPK1;IGF2R;IGF1R |
| Huntington disease Homo sapiens P00029 | 0.017107 | DYNC1H1;CREBBP;HIP1;APAF1;SP1;SIN3A;EP300;HTT;TAF4 |
| Insulin/IGF pathway-protein kinase B signaling cascade Homo sapiens P00033 | 0.02173 | IRS1;PDPK1;IGF2R;IGF1R |
| Ras Pathway Homo sapiens P04393 | 0.021853 | TIAM1;PDPK1;MAPK1;RGL1;ETS1;MAP3K4 |
| Dopamine receptor mediated signaling pathway Homo sapiens P05912 | 0.023913 | EPB41;EPB41L2;PRKAR2A;STX3;ADCY7 |
| p53 pathway Homo sapiens P00059 | 0.024778 | CREBBP;APAF1;SIN3A;PDPK1;EP300;THBS1 |
| PDGF signaling pathway Homo sapiens P00047 | 0.025924 | OPHN1;PDPK1;MAPK1;PRKCA;ITPR3;ETV3;ETS1;JAK1 |
| Histamine H2 receptor mediated signaling pathway Homo sapiens P04386 | 0.031065 | PRKAR2A;ADCY7 |
| Endothelin signaling pathway Homo sapiens P00019 | 0.031388 | PRKCE;PRKAR2A;MAPK1;PRKCA;ITPR3;ADCY7 |
| Angiogenesis Homo sapiens P00005 | 0.036862 | NOTCH2;EFNB2;PTPRB;PRKCE;MAPK1;PRKCA;ETS1;CRKL;JAK1 |
| Alzheimer disease-presenilin pathway Homo sapiens P00004 | 0.03767 | ERN1;NOTCH2;BACE1;ADAM17;LRP1;PSEN1;CD44 |
| Hypoxia response via HIF activation Homo sapiens P00030 | 0.03898 | CREBBP;ARNT;MTOR |
| Beta3 adrenergic receptor signaling pathway Homo sapiens P04379 | 0.045524 | STX3;ADCY7 |
| Histamine H1 receptor mediated signaling pathway Homo sapiens P04385 | 0.047818 | PRKCE;ITPR3;PRKCA |

## Slide 21
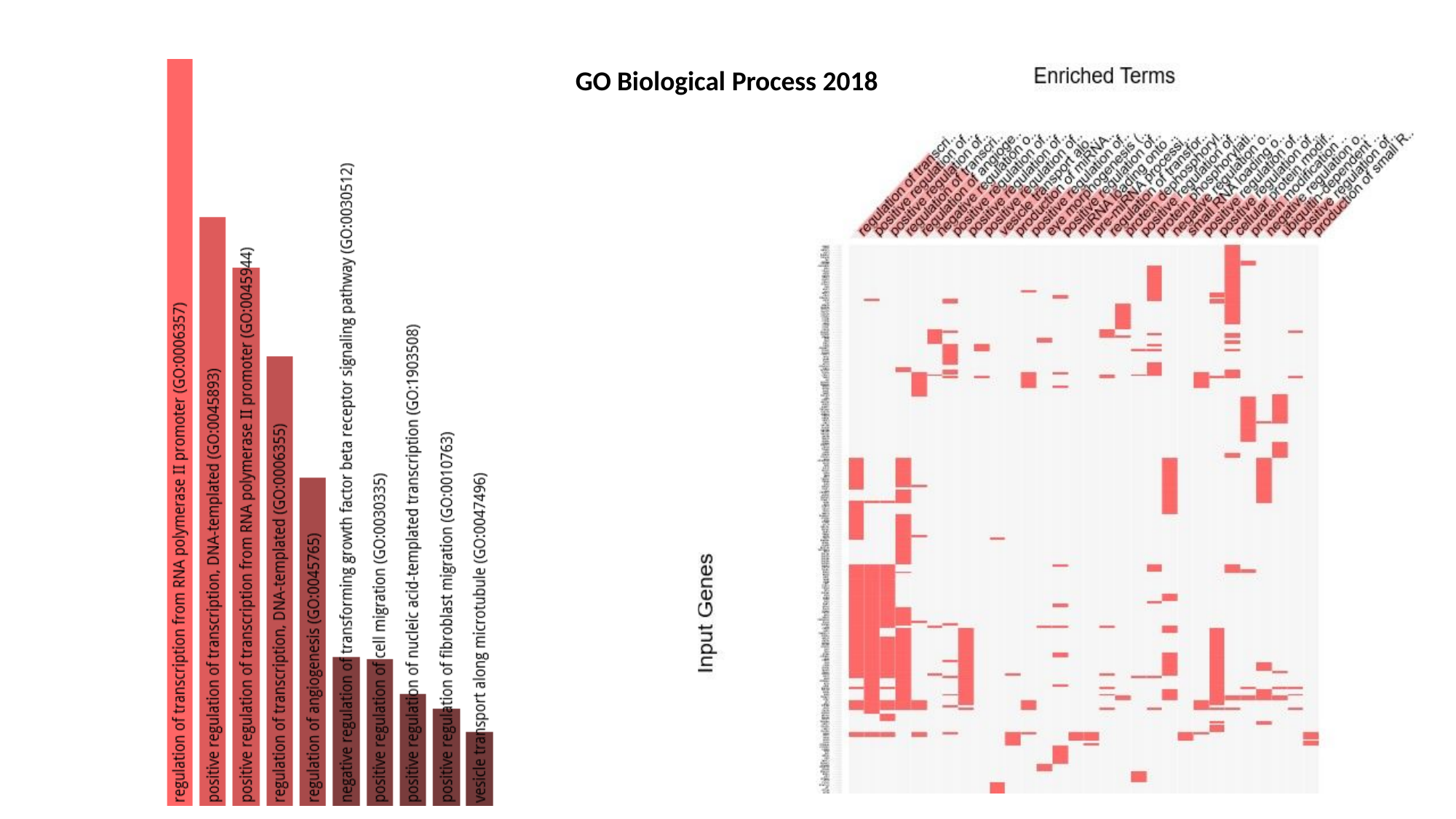

GO Biological Process 2018

## Slide 22
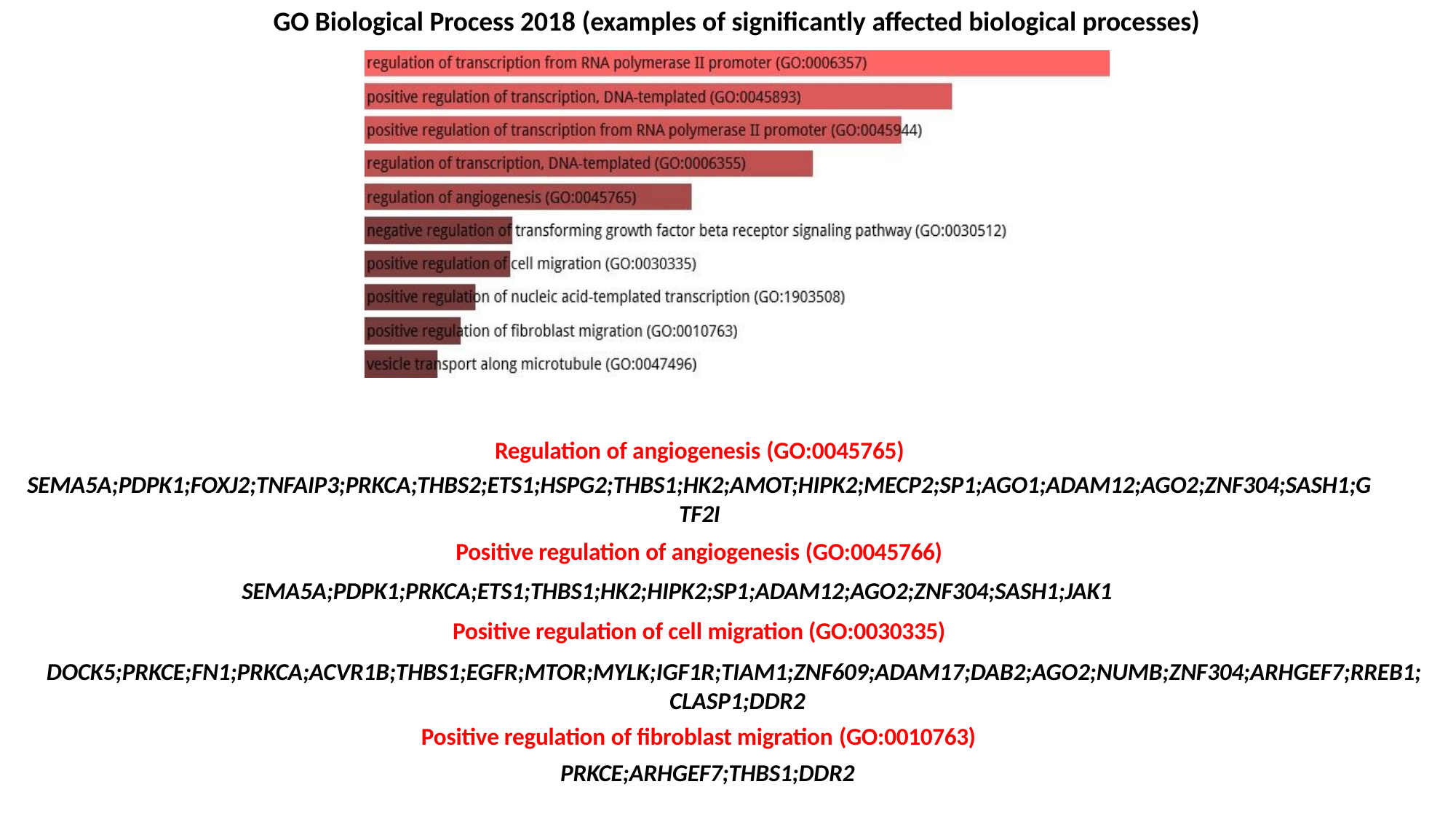

# GO Biological Process 2018 (examples of significantly affected biological processes)
Regulation of angiogenesis (GO:0045765)
SEMA5A;PDPK1;FOXJ2;TNFAIP3;PRKCA;THBS2;ETS1;HSPG2;THBS1;HK2;AMOT;HIPK2;MECP2;SP1;AGO1;ADAM12;AGO2;ZNF304;SASH1;GTF2I
Positive regulation of angiogenesis (GO:0045766)
SEMA5A;PDPK1;PRKCA;ETS1;THBS1;HK2;HIPK2;SP1;ADAM12;AGO2;ZNF304;SASH1;JAK1
Positive regulation of cell migration (GO:0030335)
DOCK5;PRKCE;FN1;PRKCA;ACVR1B;THBS1;EGFR;MTOR;MYLK;IGF1R;TIAM1;ZNF609;ADAM17;DAB2;AGO2;NUMB;ZNF304;ARHGEF7;RREB1; CLASP1;DDR2
Positive regulation of fibroblast migration (GO:0010763)
PRKCE;ARHGEF7;THBS1;DDR2

## Slide 23
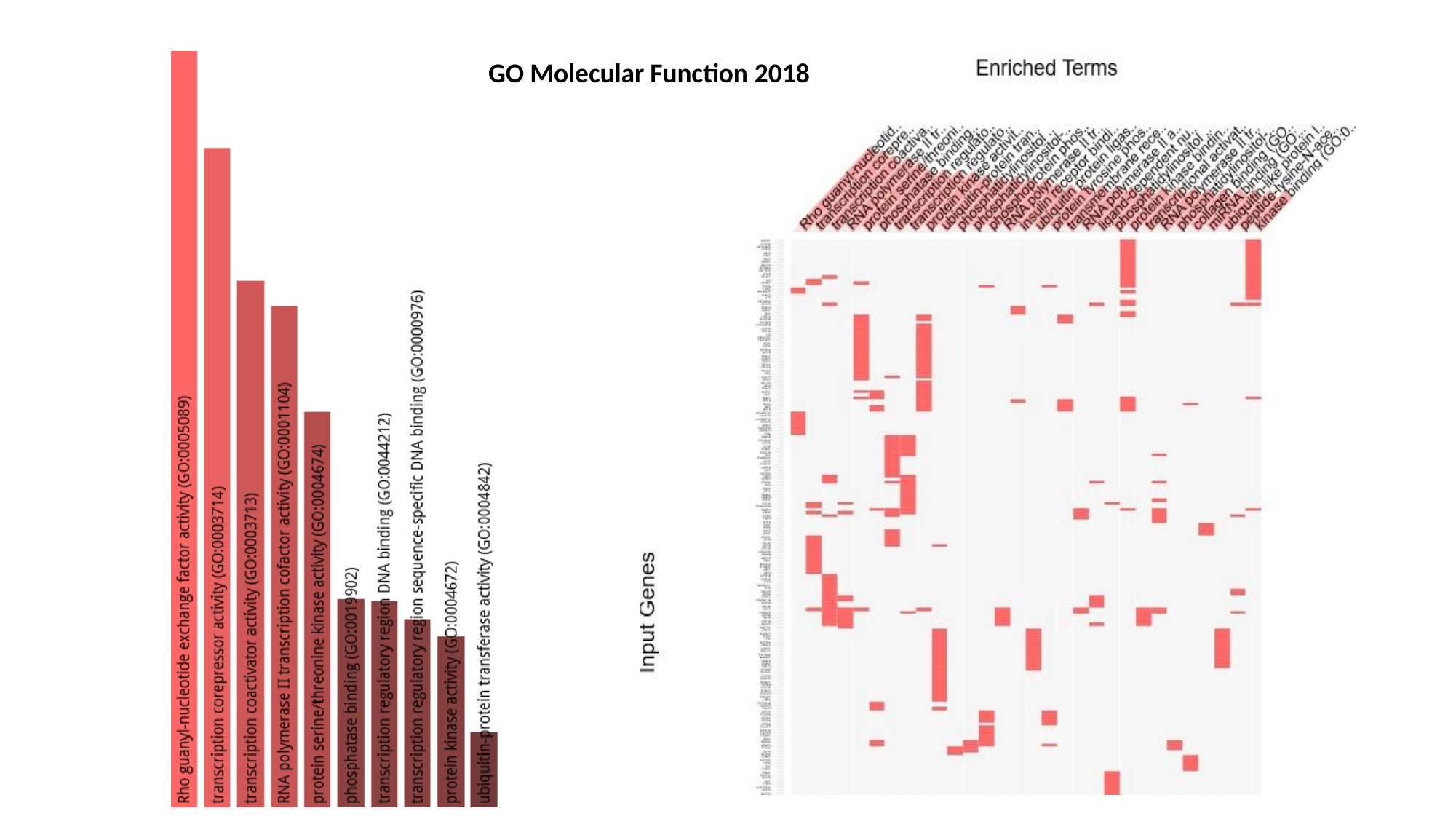

GO Molecular Function 2018

## Slide 24
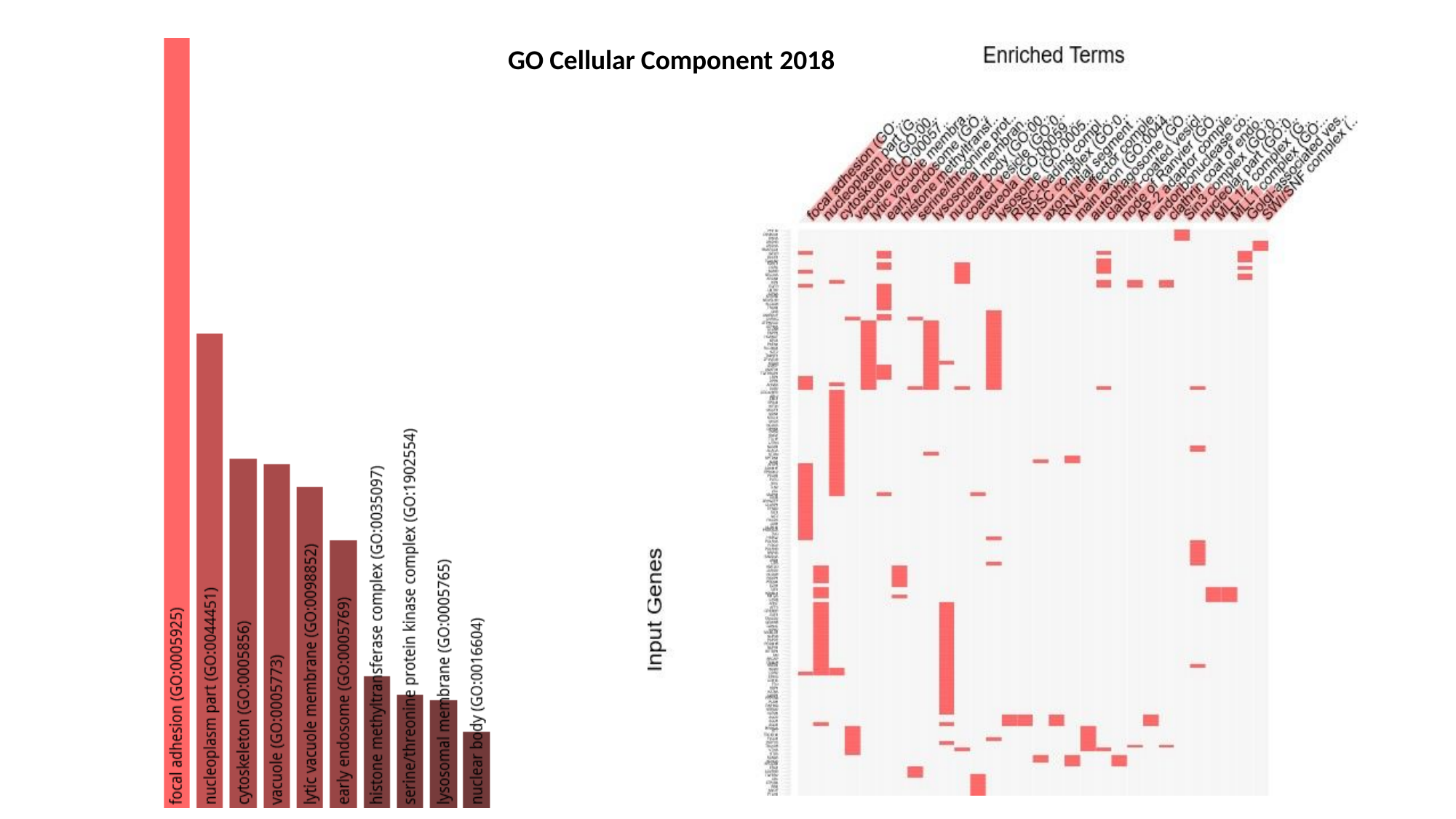

GO Cellular Component 2018

## Slide 25
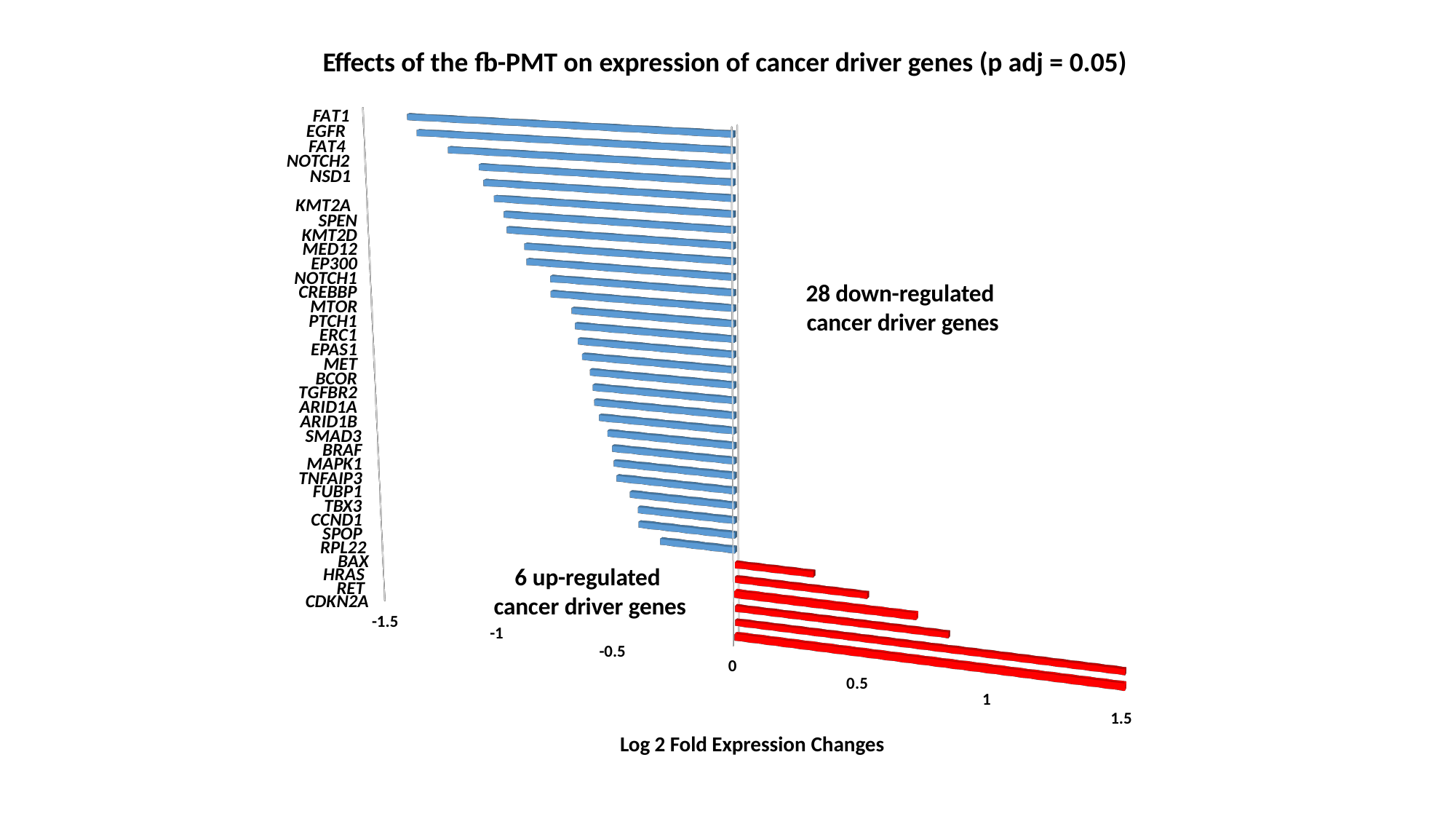

# Effects of the fb-PMT on expression of cancer driver genes (p adj = 0.05)
FAT1 EGFR FAT4 NOTCH2
NSD1 KMT2A
SPEN KMT2D MED12 EP300 NOTCH1 CREBBP MTOR PTCH1 ERC1 EPAS1 MET BCOR TGFBR2 ARID1A ARID1B SMAD3
BRAF MAPK1 TNFAIP3 FUBP1 TBX3 CCND1 SPOP RPL22
BAX HRAS RET CDKN2A
28 down-regulated cancer driver genes
6 up-regulated
cancer driver genes
-1
-0.5
-1.5
0
0.5
1
1.5
Log 2 Fold Expression Changes

## Slide 26
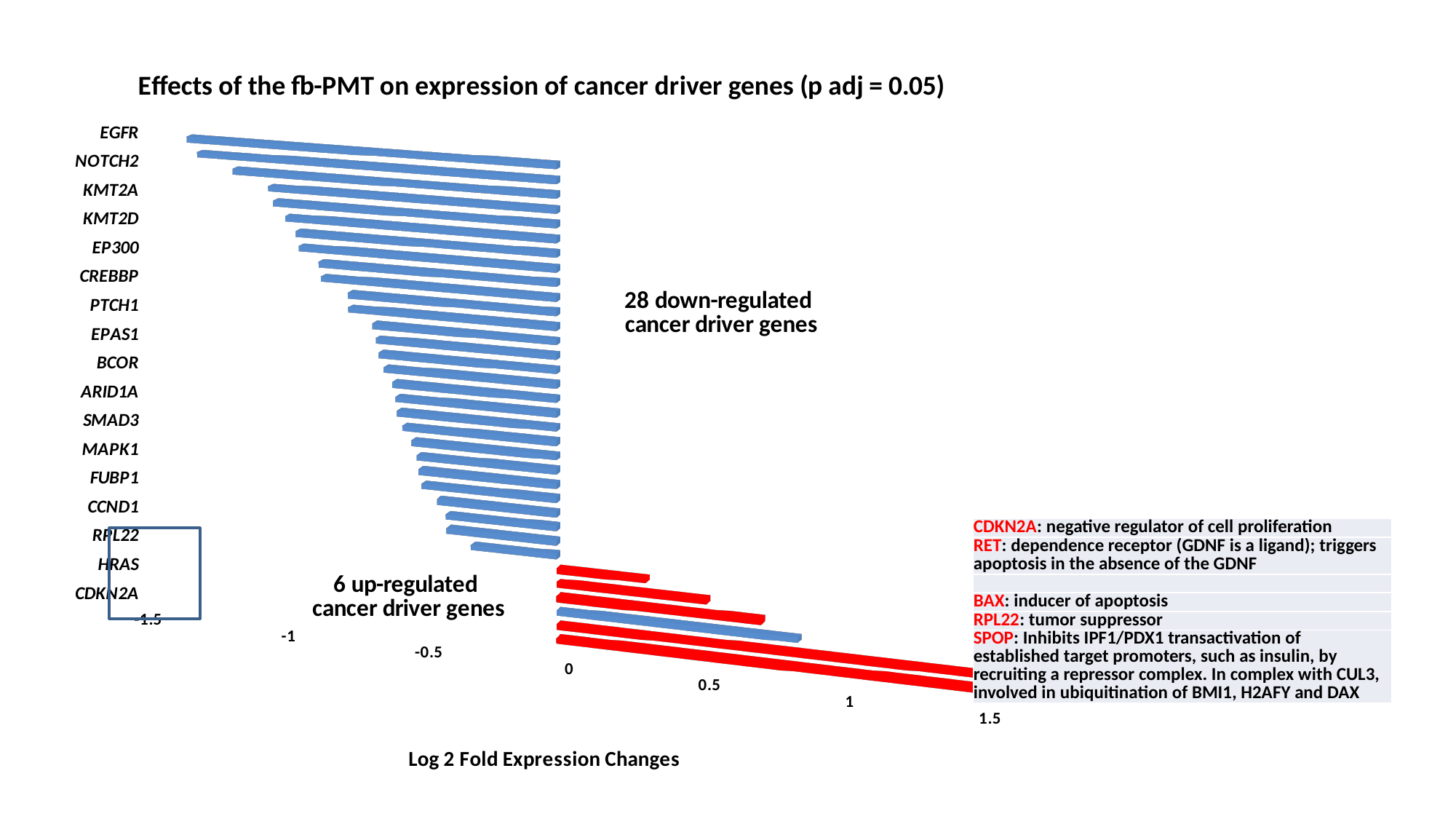

[unsupported chart]
| CDKN2A: negative regulator of cell proliferation |
| --- |
| RET: dependence receptor (GDNF is a ligand); triggers apoptosis in the absence of the GDNF |
| |
| BAX: inducer of apoptosis |
| RPL22: tumor suppressor |
| SPOP: Inhibits IPF1/PDX1 transactivation of established target promoters, such as insulin, by recruiting a repressor complex. In complex with CUL3, involved in ubiquitination of BMI1, H2AFY and DAX |

## Slide 27
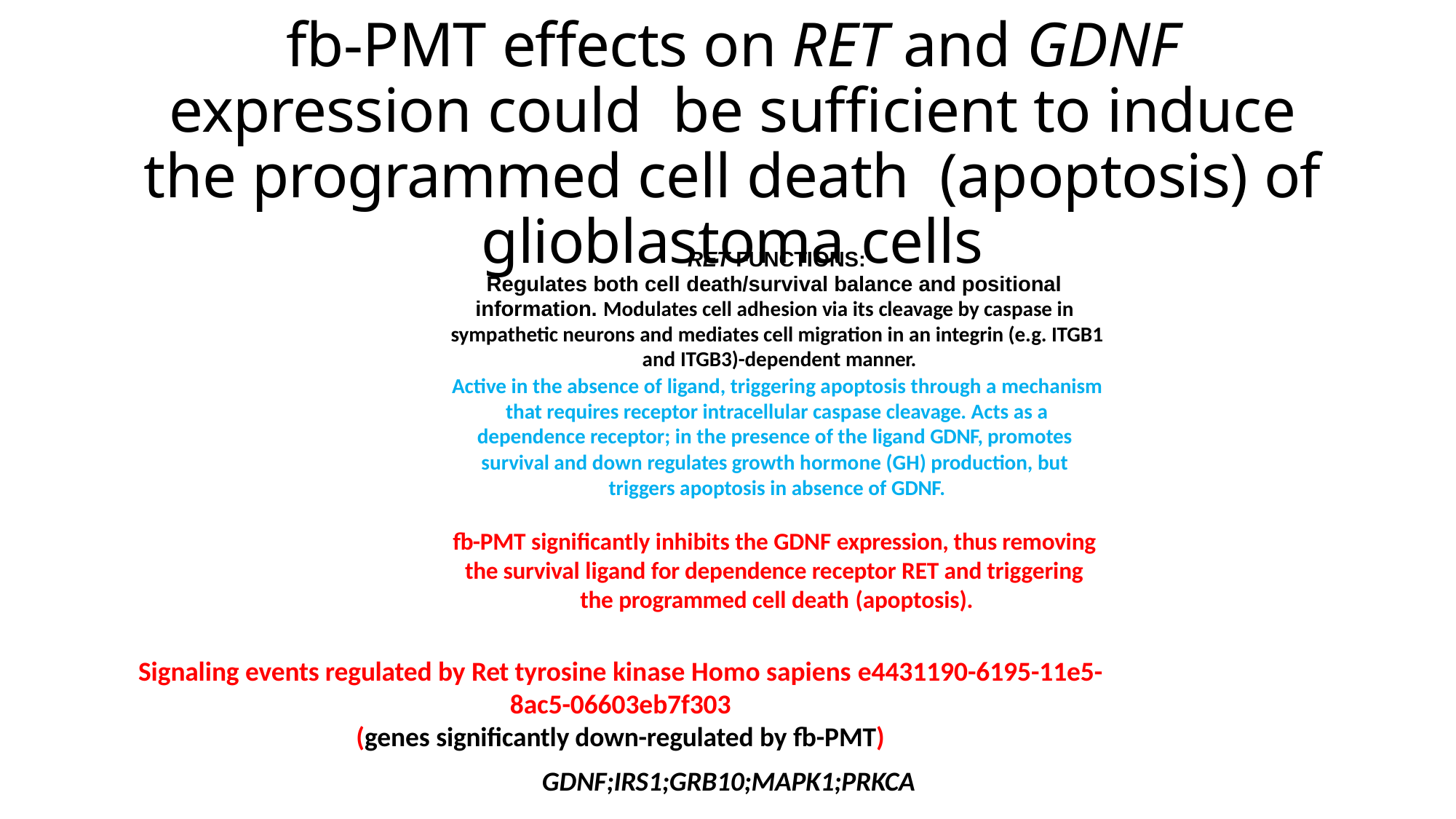

# fb-PMT effects on RET and GDNF expression could be sufficient to induce the programmed cell death (apoptosis) of glioblastoma cells
RET FUNCTIONS:
Regulates both cell death/survival balance and positional information. Modulates cell adhesion via its cleavage by caspase in sympathetic neurons and mediates cell migration in an integrin (e.g. ITGB1 and ITGB3)-dependent manner.
Active in the absence of ligand, triggering apoptosis through a mechanism that requires receptor intracellular caspase cleavage. Acts as a dependence receptor; in the presence of the ligand GDNF, promotes survival and down regulates growth hormone (GH) production, but triggers apoptosis in absence of GDNF.
fb-PMT significantly inhibits the GDNF expression, thus removing the survival ligand for dependence receptor RET and triggering the programmed cell death (apoptosis).
Signaling events regulated by Ret tyrosine kinase Homo sapiens e4431190-6195-11e5-8ac5-06603eb7f303
(genes significantly down-regulated by fb-PMT)
GDNF;IRS1;GRB10;MAPK1;PRKCA

## Slide 28
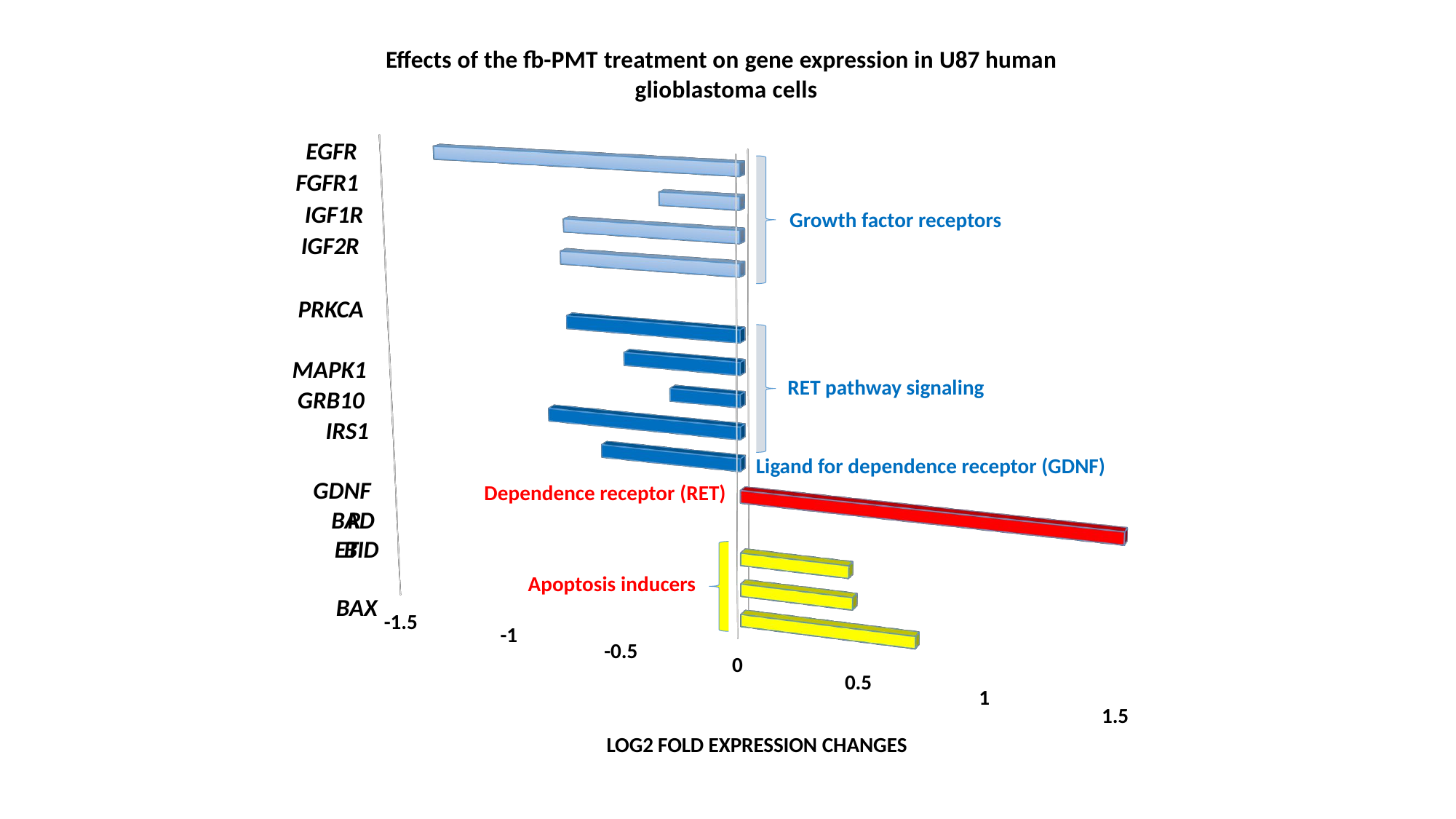

Effects of the fb-PMT treatment on gene expression in U87 human glioblastoma cells
EGFR FGFR1 IGF1R IGF2R
Growth factor receptors
PRKCA MAPK1 GRB10
IRS1 GDNF
RET
RET pathway signaling
Ligand for dependence receptor (GDNF)
Dependence receptor (RET)
BAD BID BAX
Apoptosis inducers
-1.5
-1
-0.5
0
0.5
1
1.5
LOG2 FOLD EXPRESSION CHANGES

## Slide 29
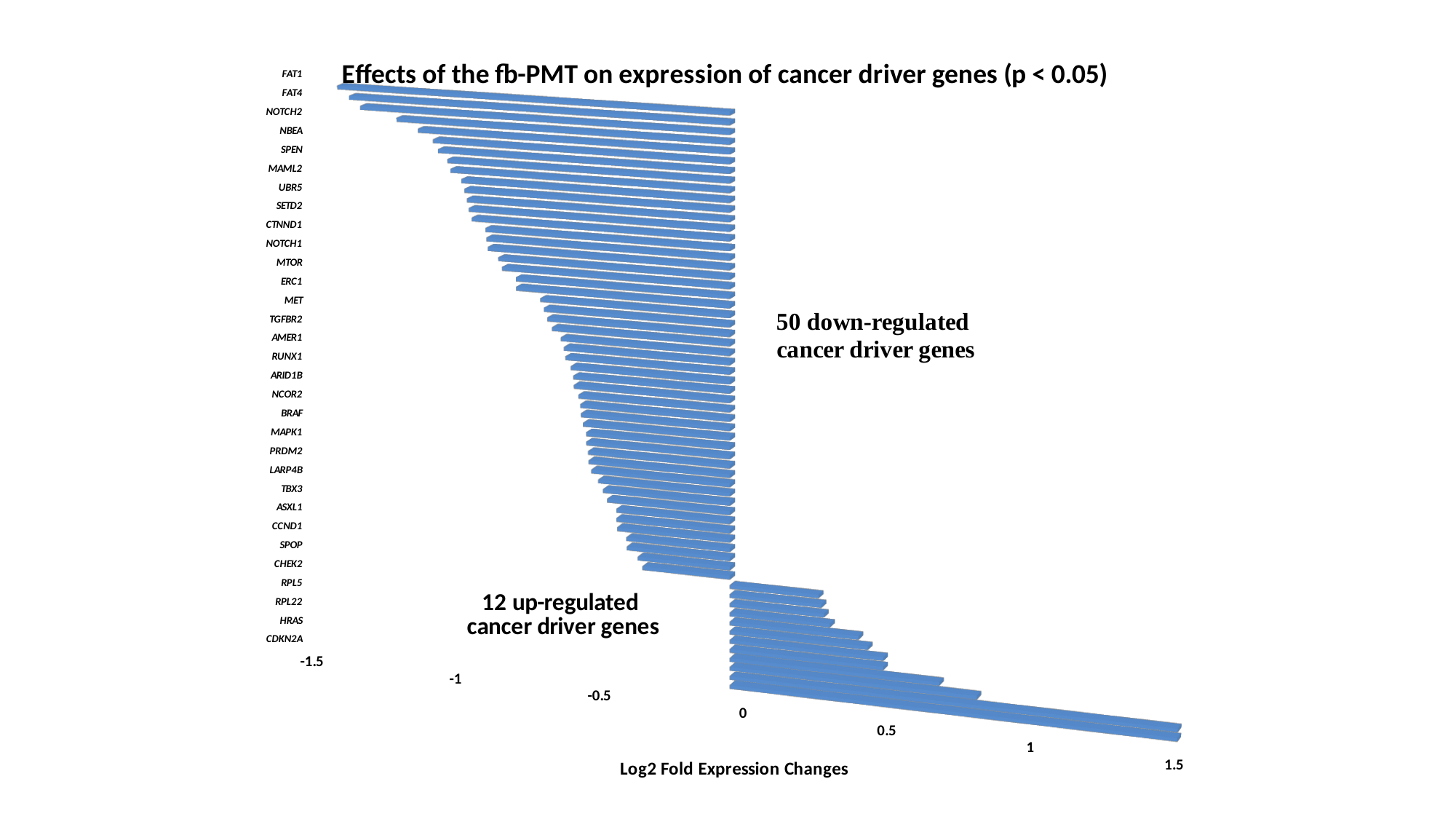

[unsupported chart]
