# Supplementary material for: Discovery of novel thyrointegrin αvβ3 antagonist fb-PMT (NP751) in the management of human glioblastoma multiforme
Source: Neurooncol Adv. 2022 Dec 8;5(1):vdac180. doi: 10.1093/noajnl/vdac180 (PMC9985163; doi:10.1093/noajnl/vdac180)

## Slide 1
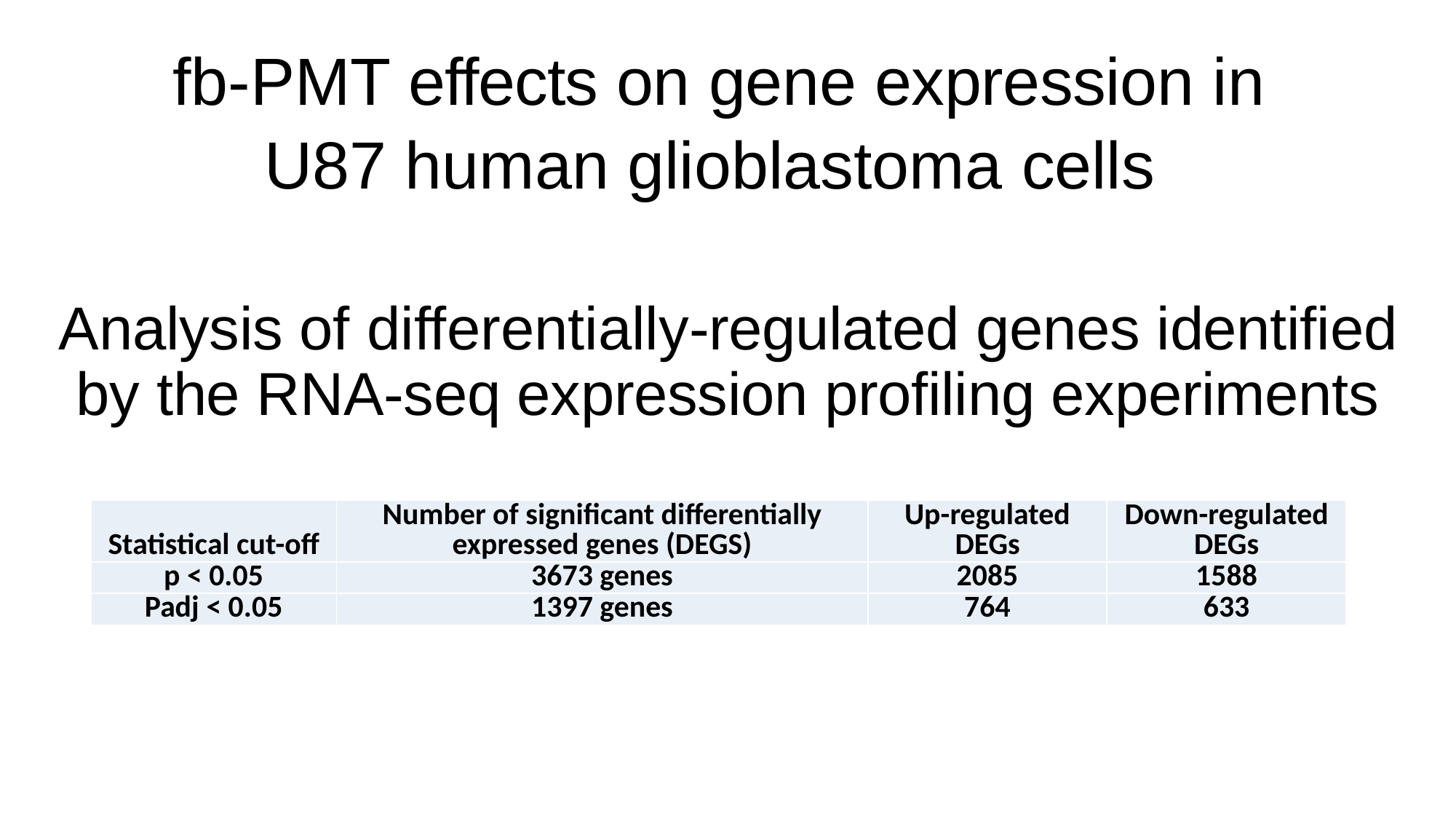

fb-PMT effects on gene expression in
U87 human glioblastoma cells
Analysis of differentially-regulated genes identified by the RNA-seq expression profiling experiments
| Statistical cut-off | Number of significant differentially expressed genes (DEGS) | Up-regulated DEGs | Down-regulated DEGs |
| --- | --- | --- | --- |
| p < 0.05 | 3673 genes | 2085 | 1588 |
| Padj < 0.05 | 1397 genes | 764 | 633 |

## Slide 2
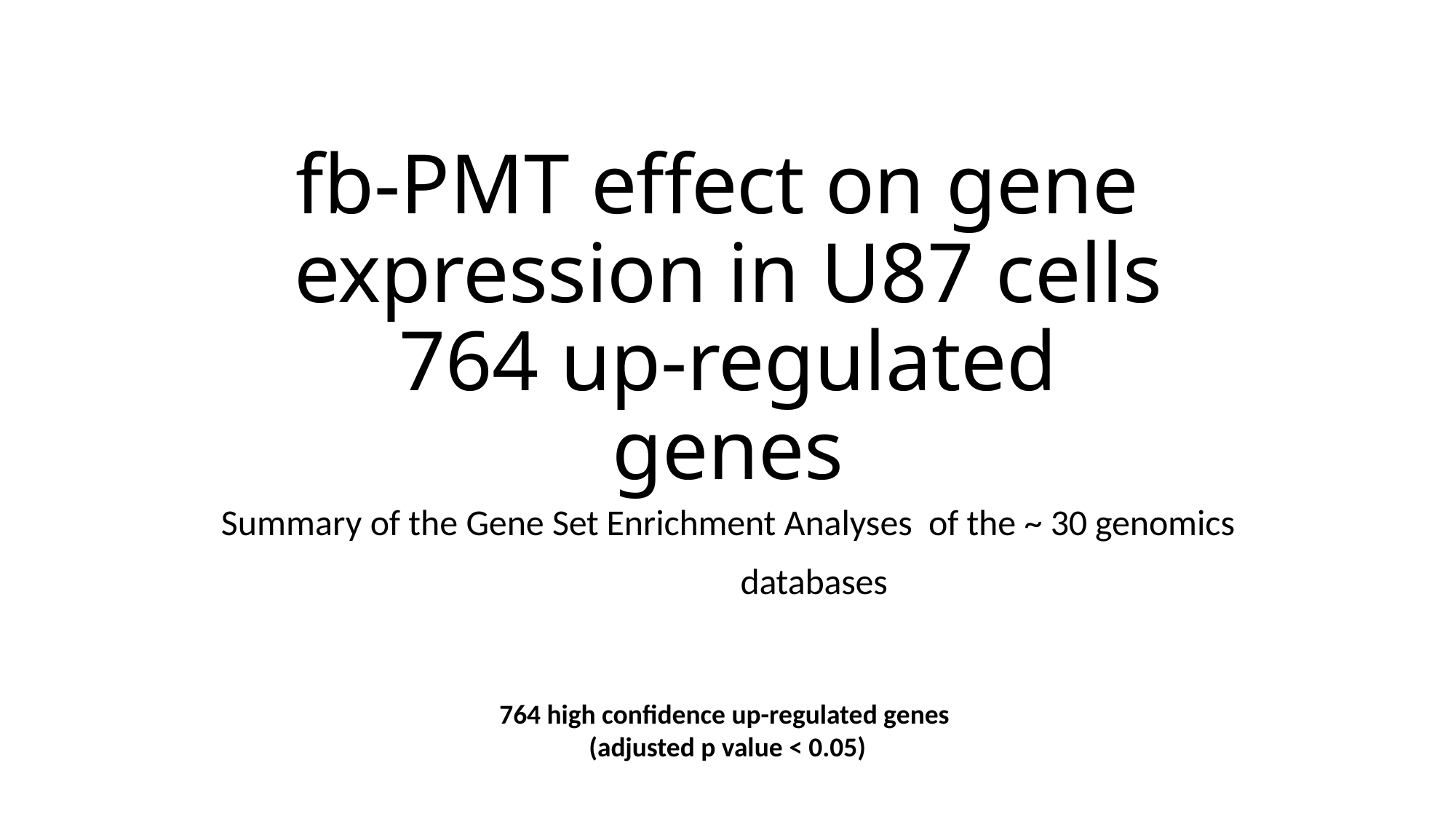

fb-PMT effect on gene expression in U87 cells 764 up-regulated genes
Summary of the Gene Set Enrichment Analyses of the ~ 30 genomics databases
764 high confidence up-regulated genes
(adjusted p value < 0.05)

## Slide 3
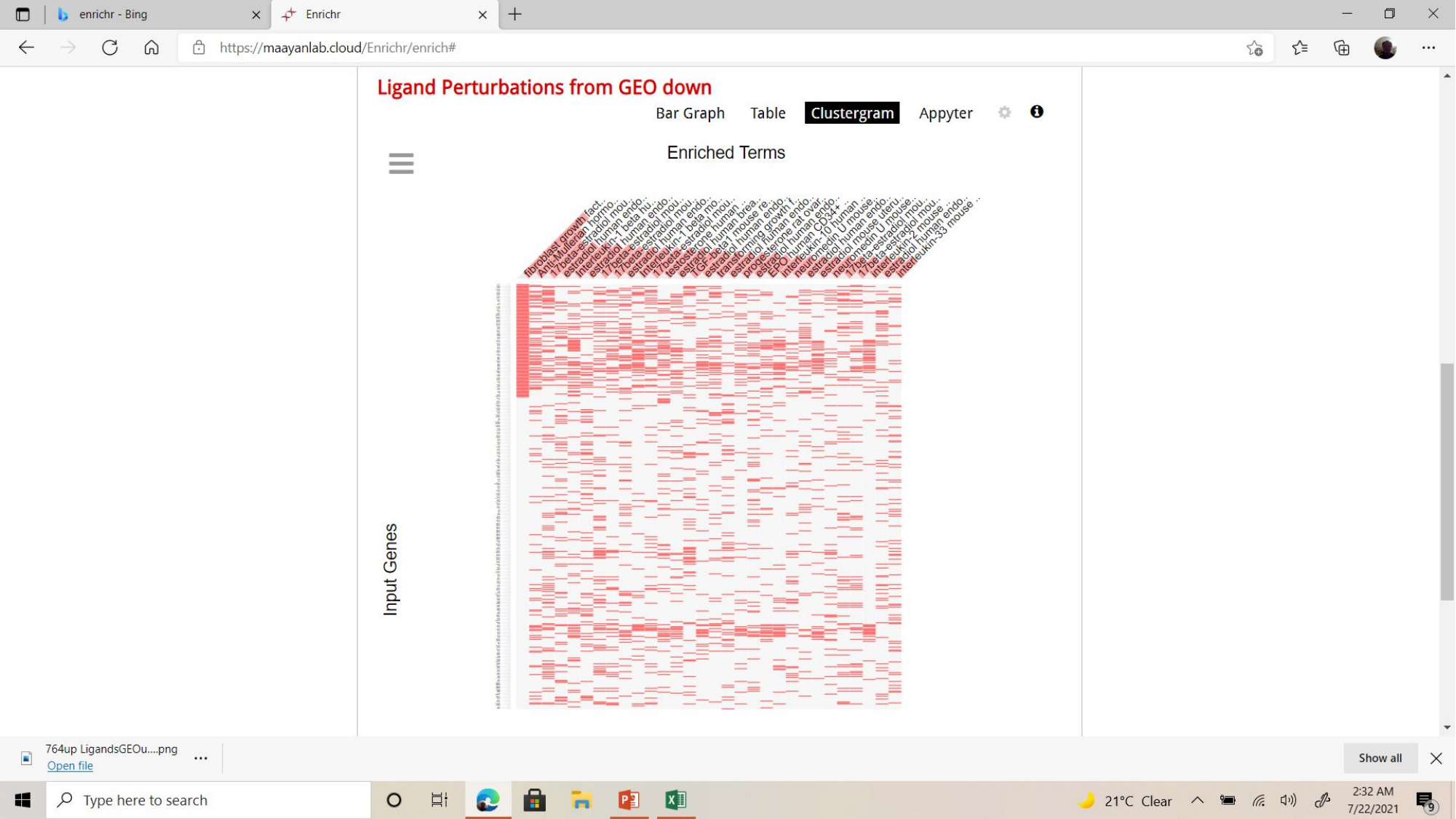

## Slide 4
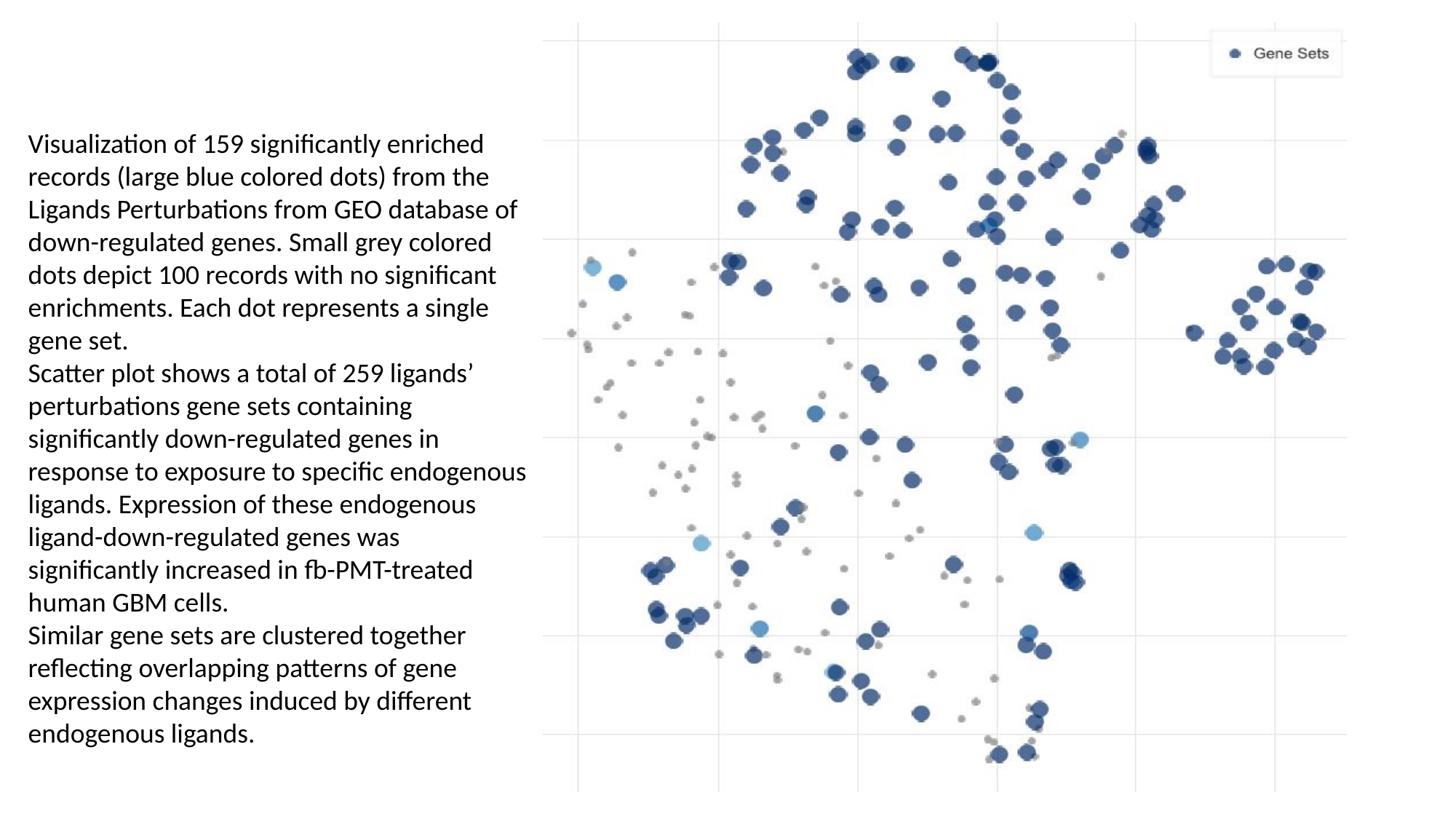

Visualization of 159 significantly enriched records (large blue colored dots) from the Ligands Perturbations from GEO database of down-regulated genes. Small grey colored dots depict 100 records with no significant enrichments. Each dot represents a single gene set.
Scatter plot shows a total of 259 ligands’ perturbations gene sets containing significantly down-regulated genes in response to exposure to specific endogenous ligands. Expression of these endogenous ligand-down-regulated genes was significantly increased in fb-PMT-treated human GBM cells.
Similar gene sets are clustered together reflecting overlapping patterns of gene expression changes induced by different endogenous ligands.

## Slide 5
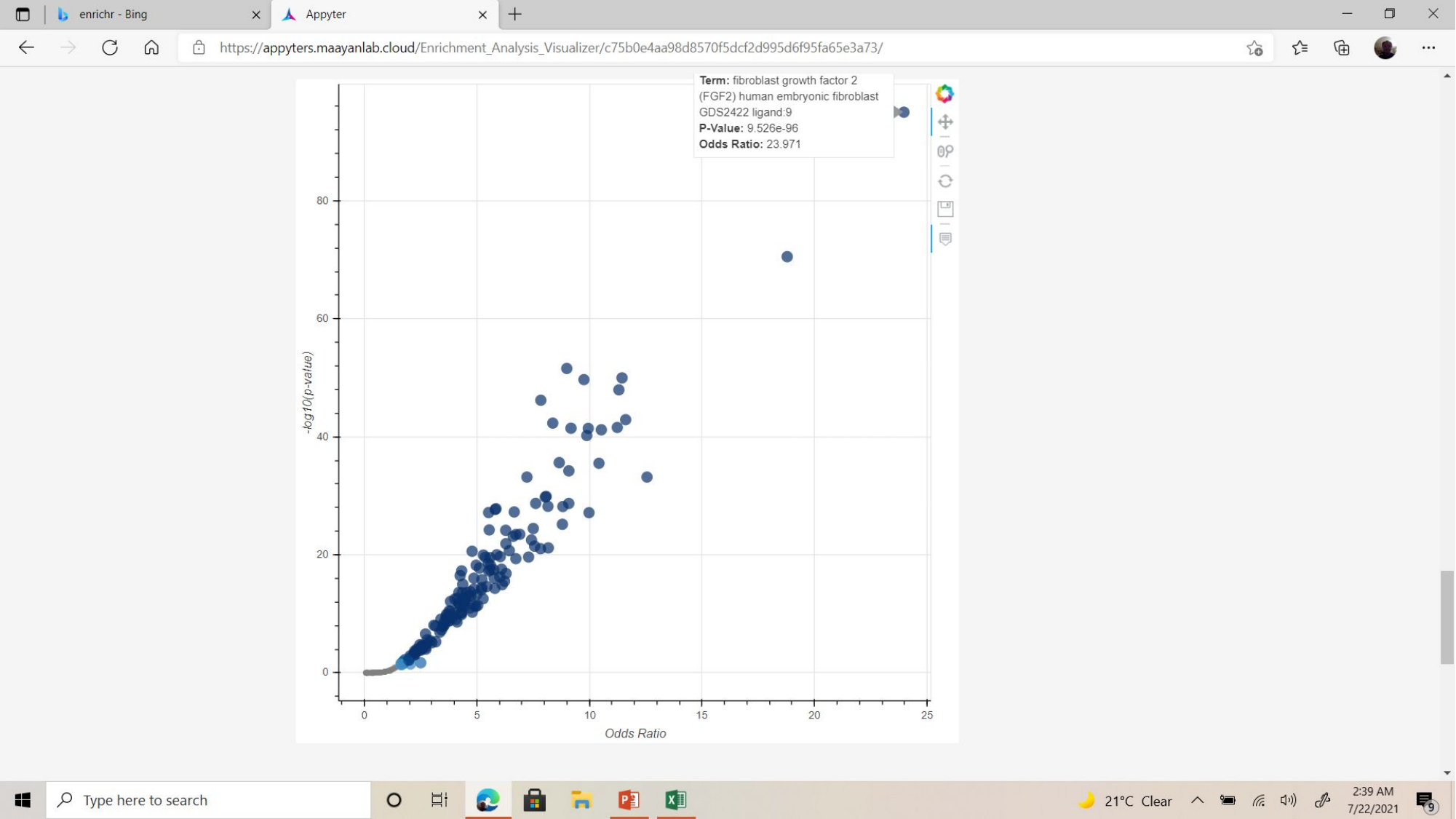

## Slide 6
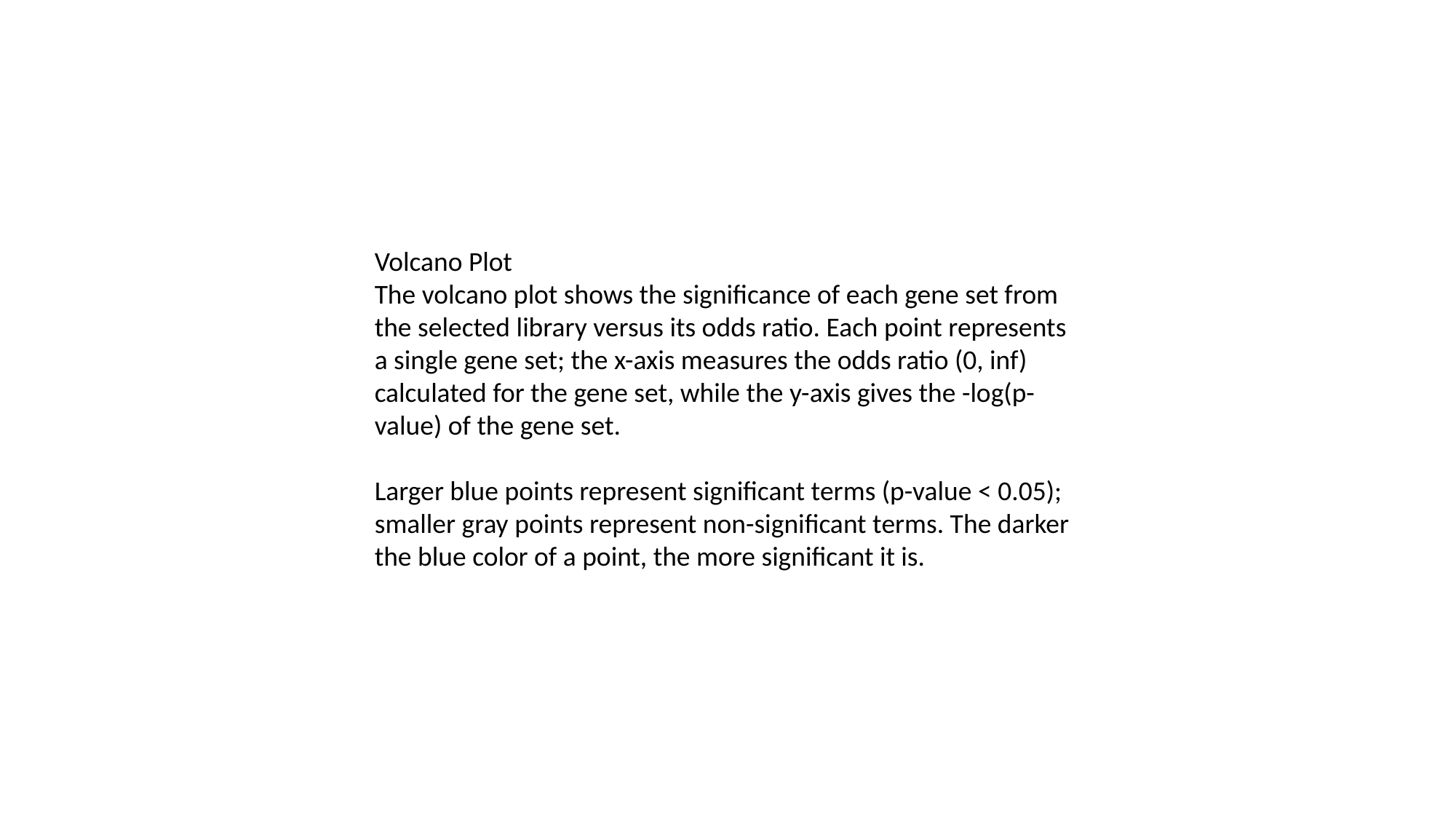

Volcano Plot
The volcano plot shows the significance of each gene set from the selected library versus its odds ratio. Each point represents a single gene set; the x-axis measures the odds ratio (0, inf) calculated for the gene set, while the y-axis gives the -log(p-value) of the gene set.
Larger blue points represent significant terms (p-value < 0.05); smaller gray points represent non-significant terms. The darker the blue color of a point, the more significant it is.

## Slide 7
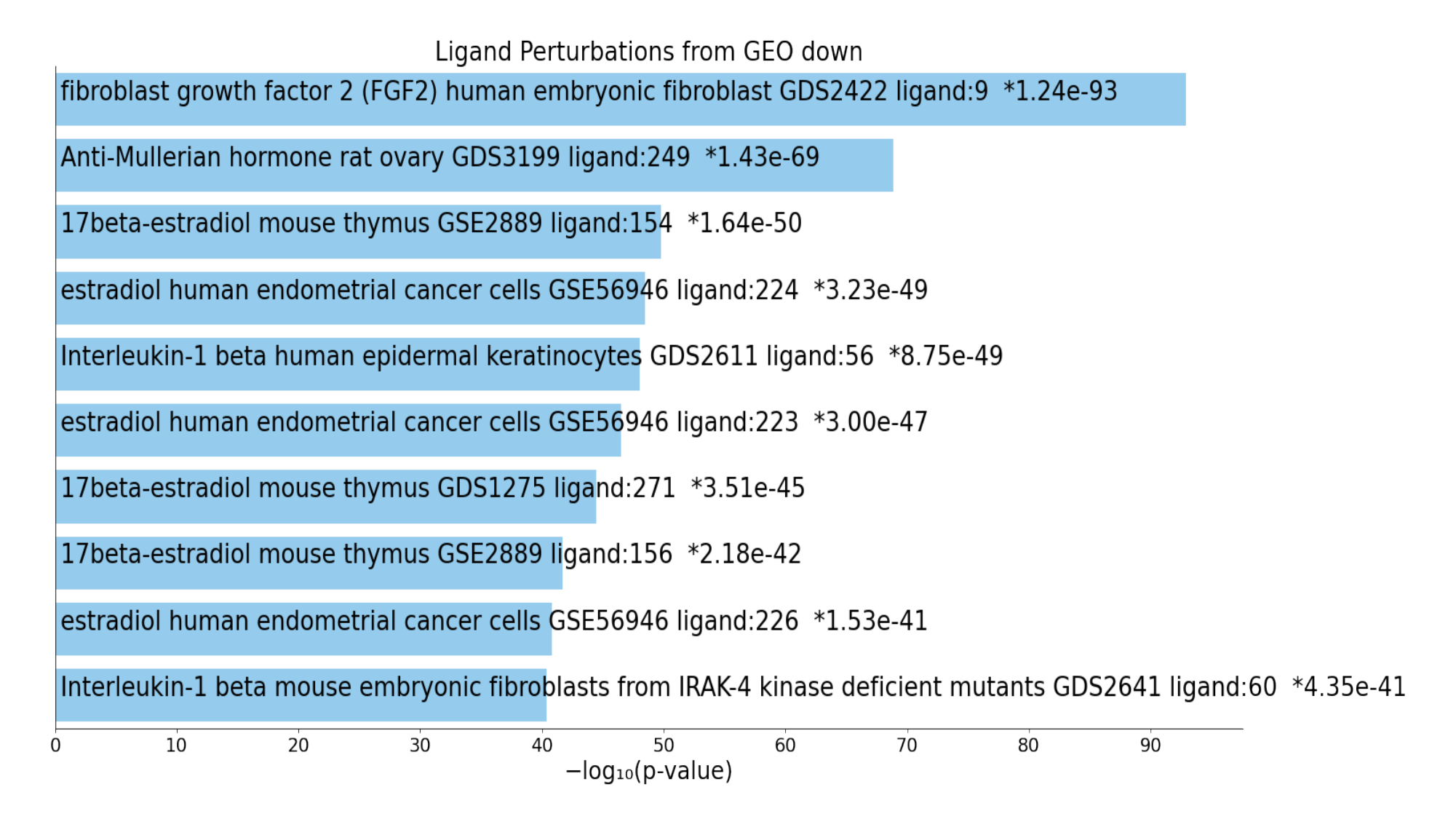

## Slide 8
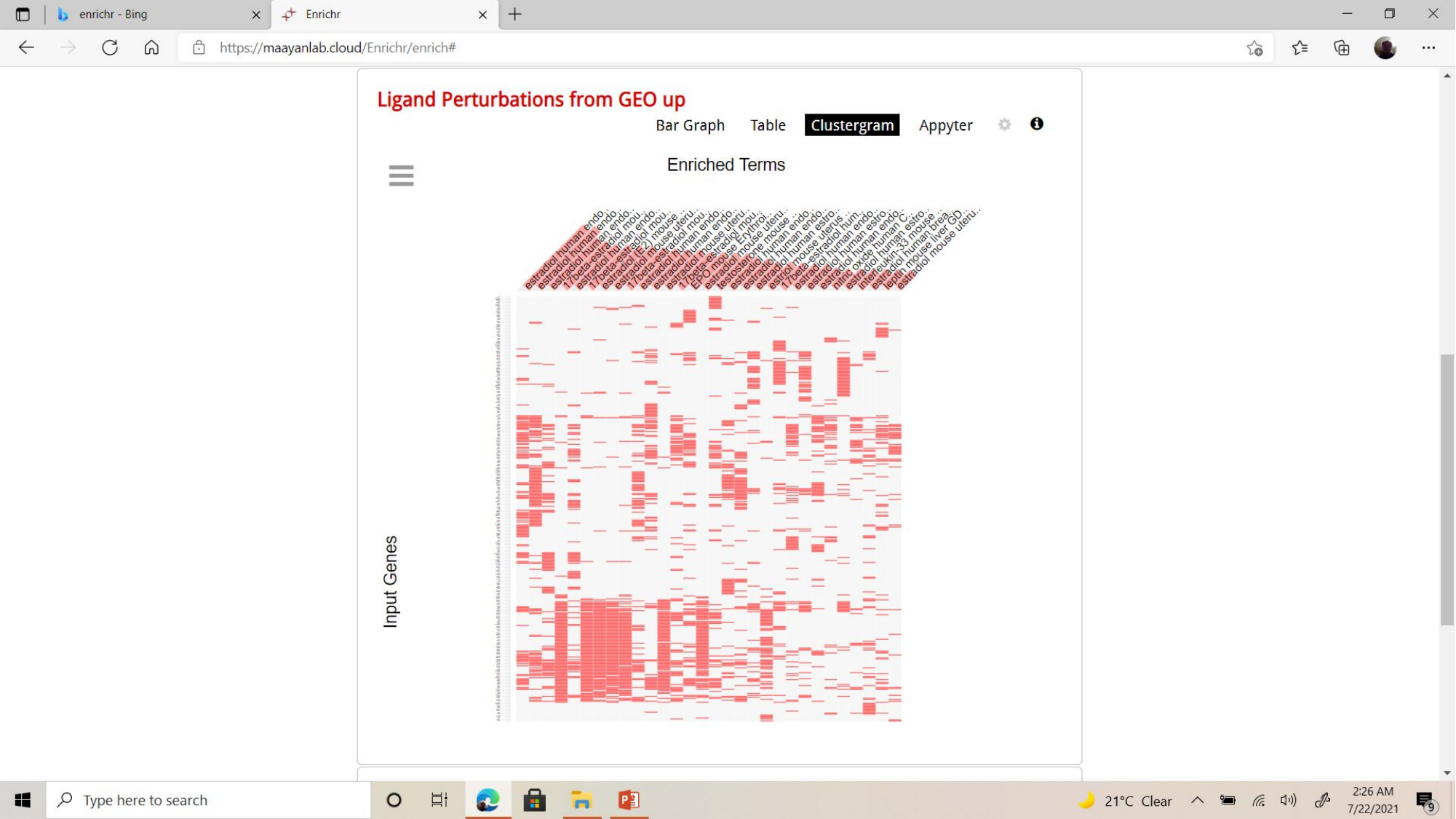

## Slide 9
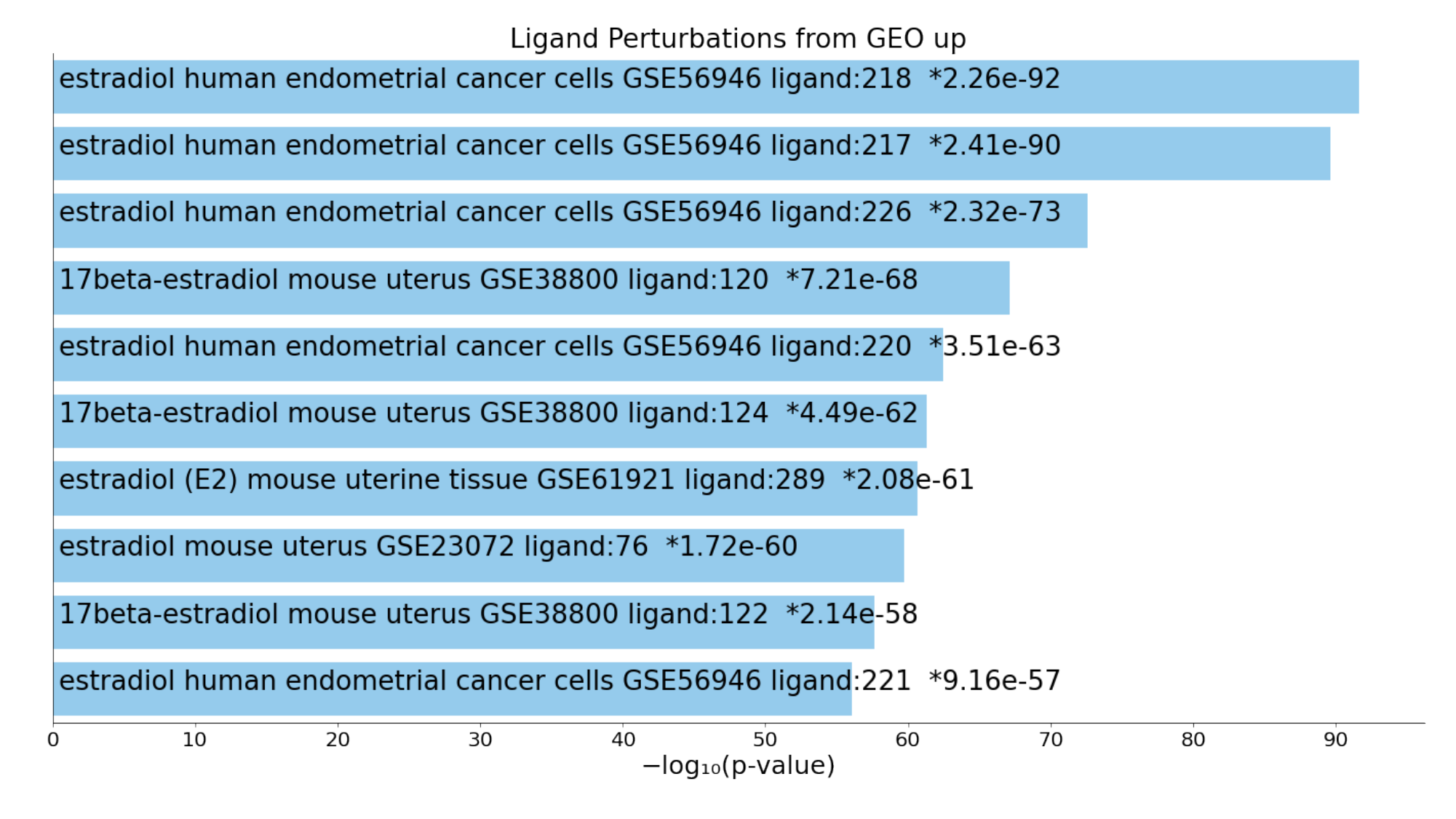

## Slide 10
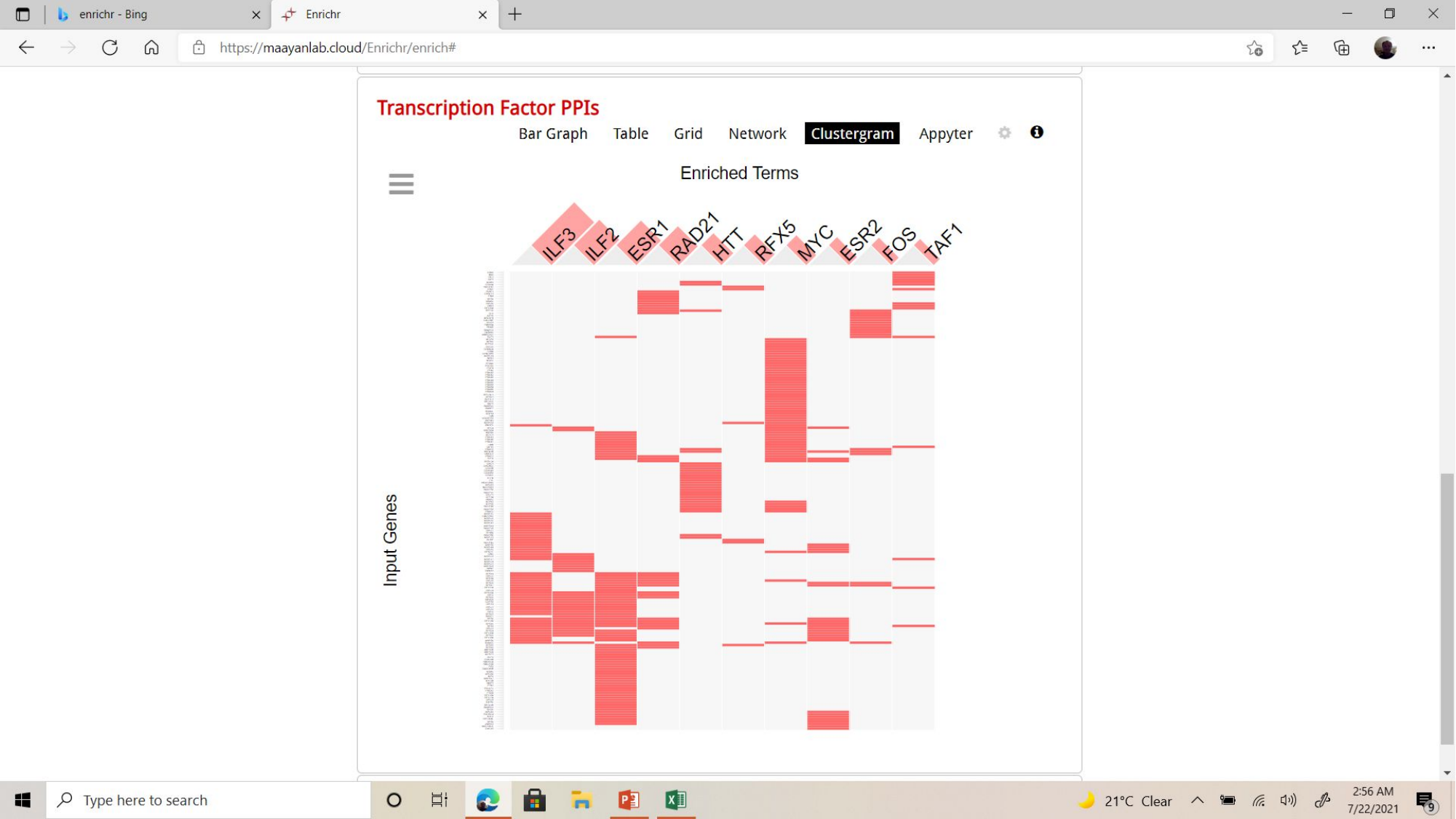

## Slide 11
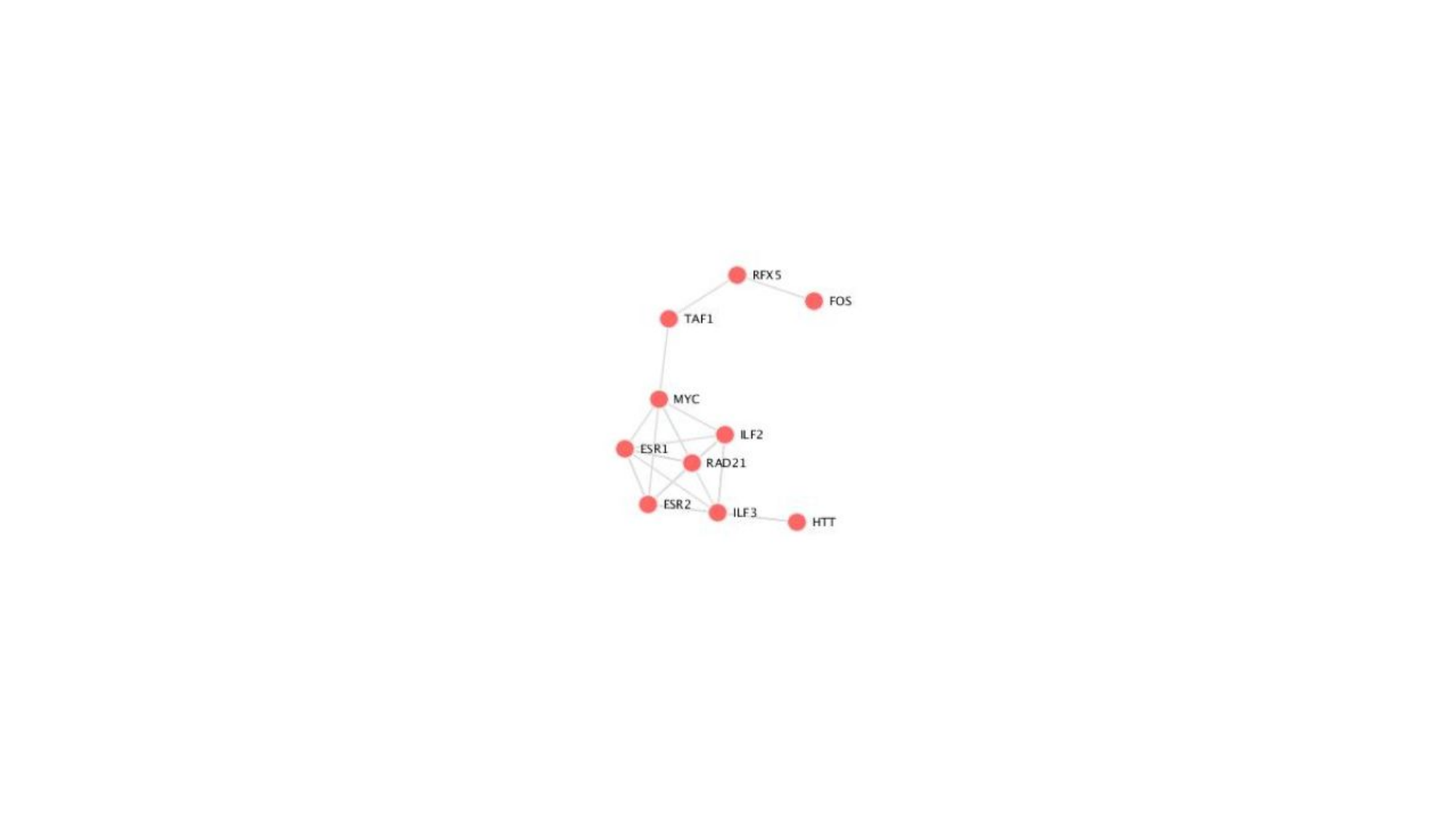

## Slide 12
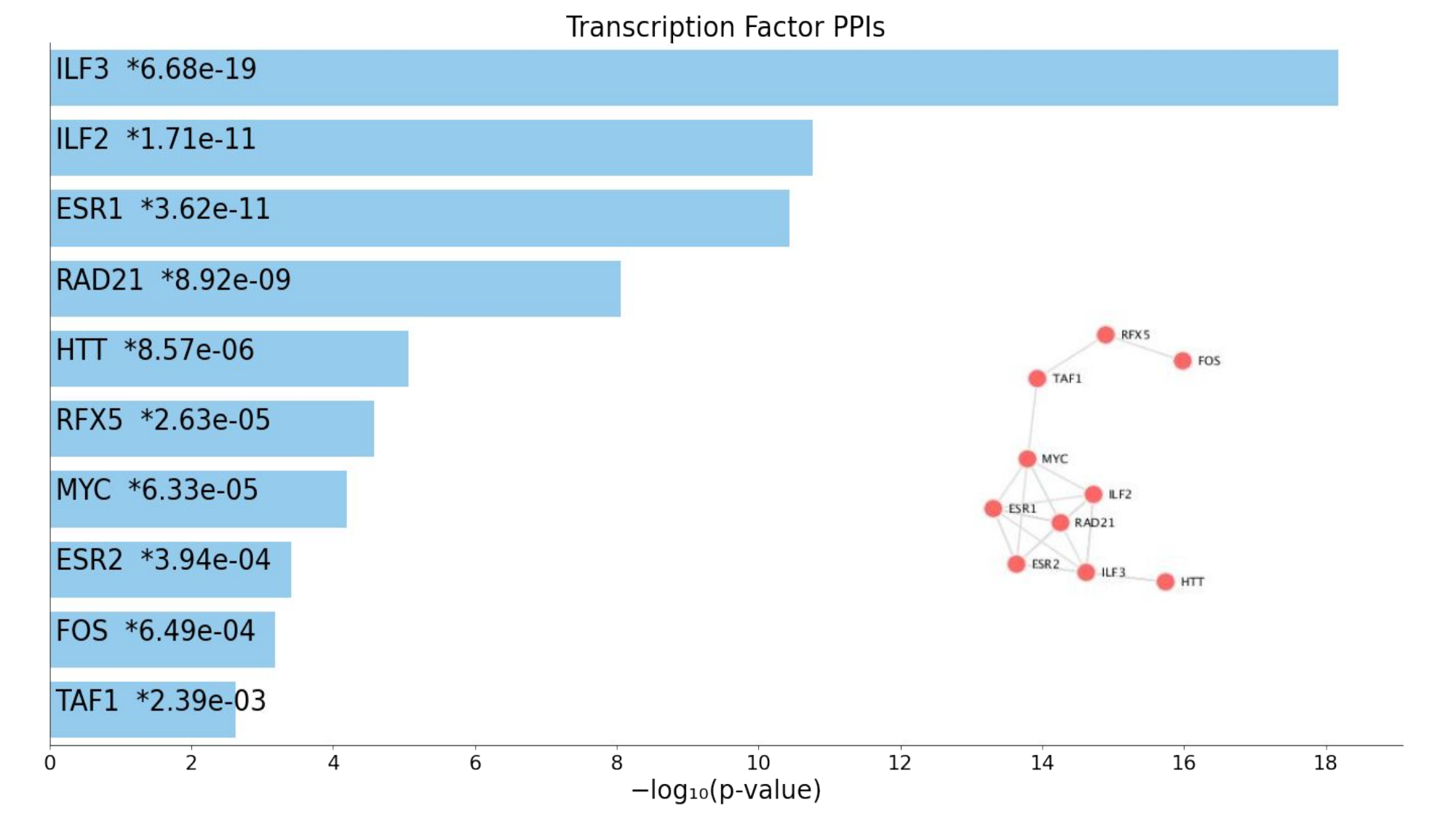

## Slide 13
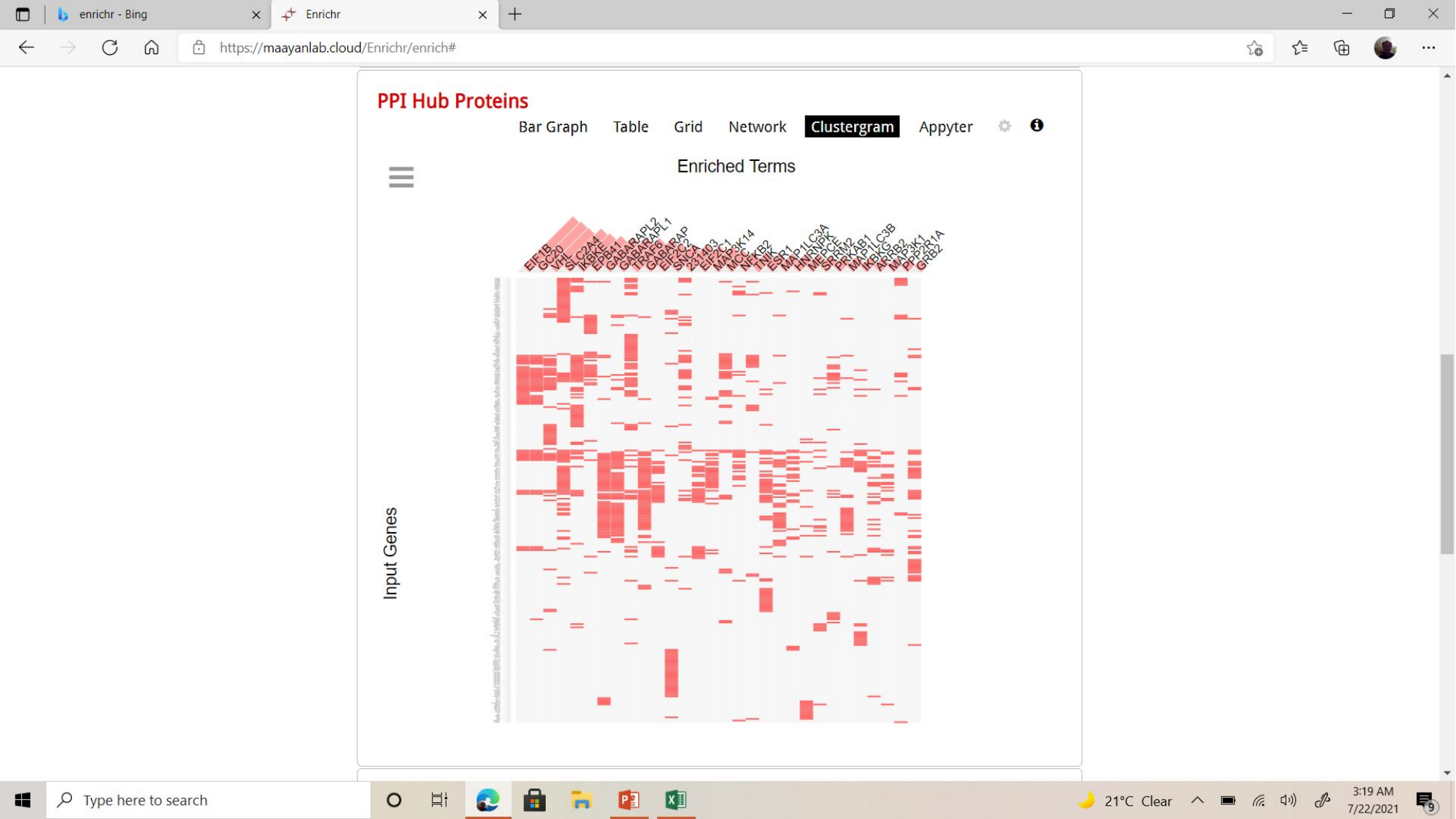

## Slide 14
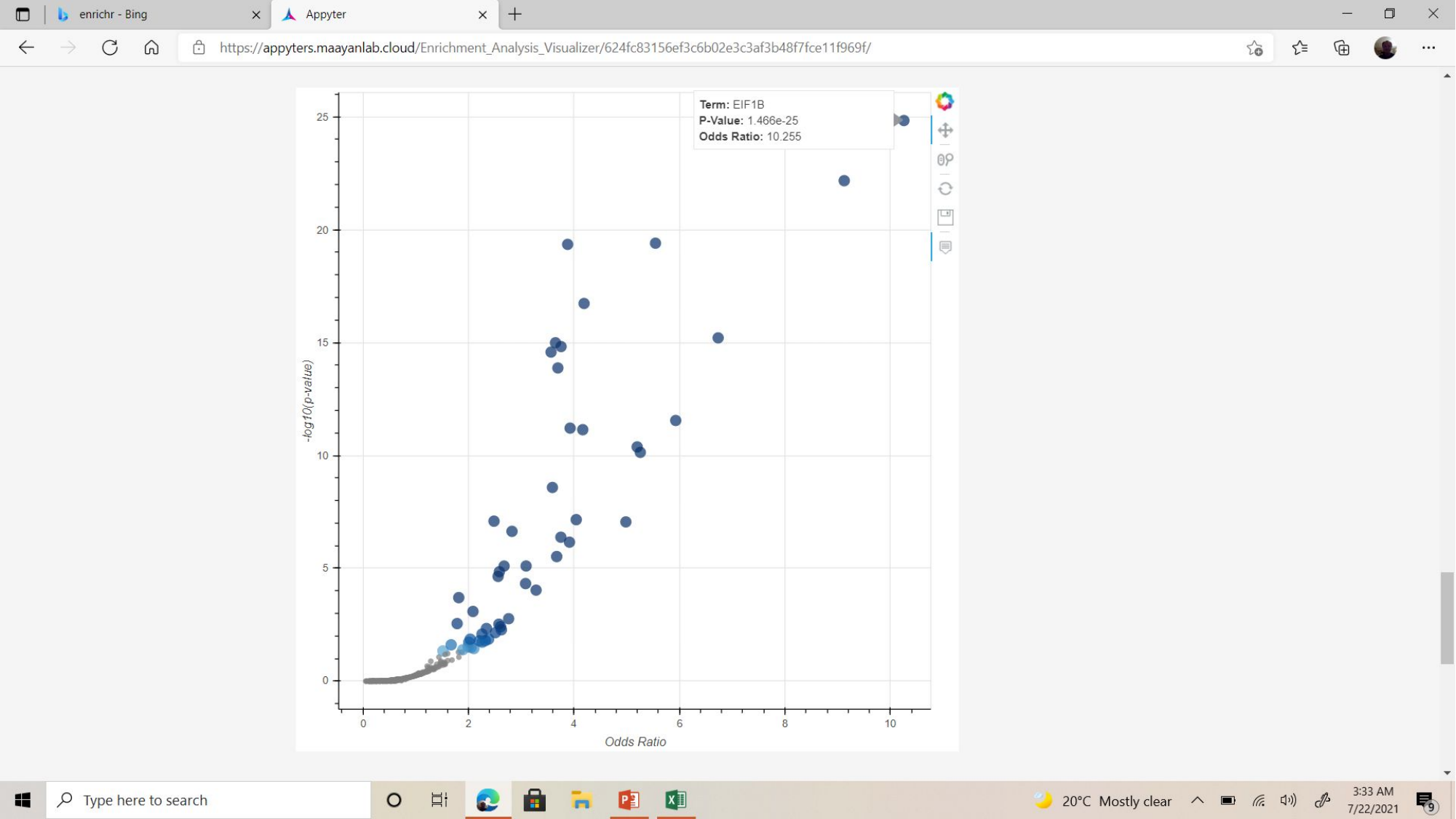

## Slide 15
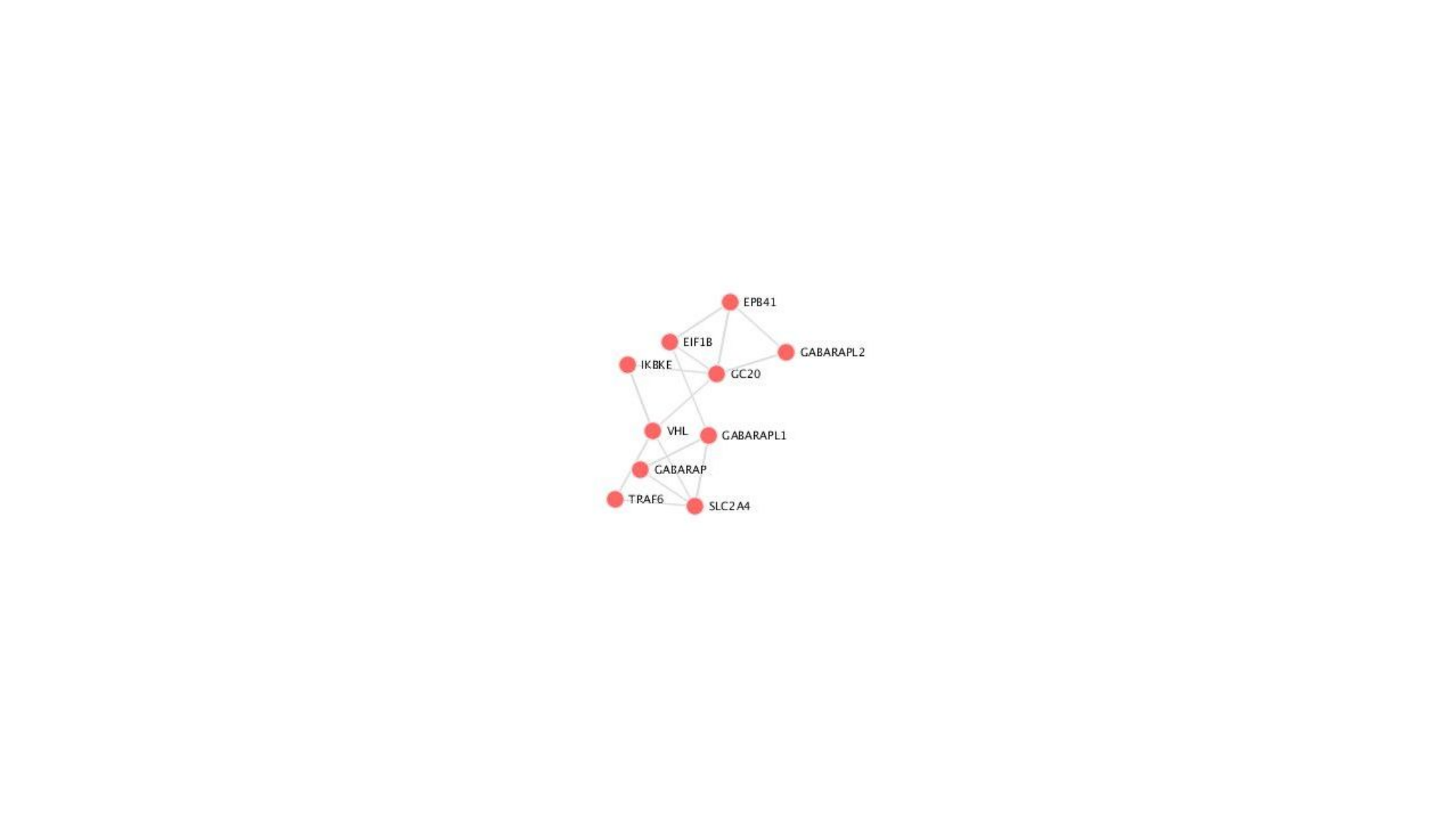

## Slide 16
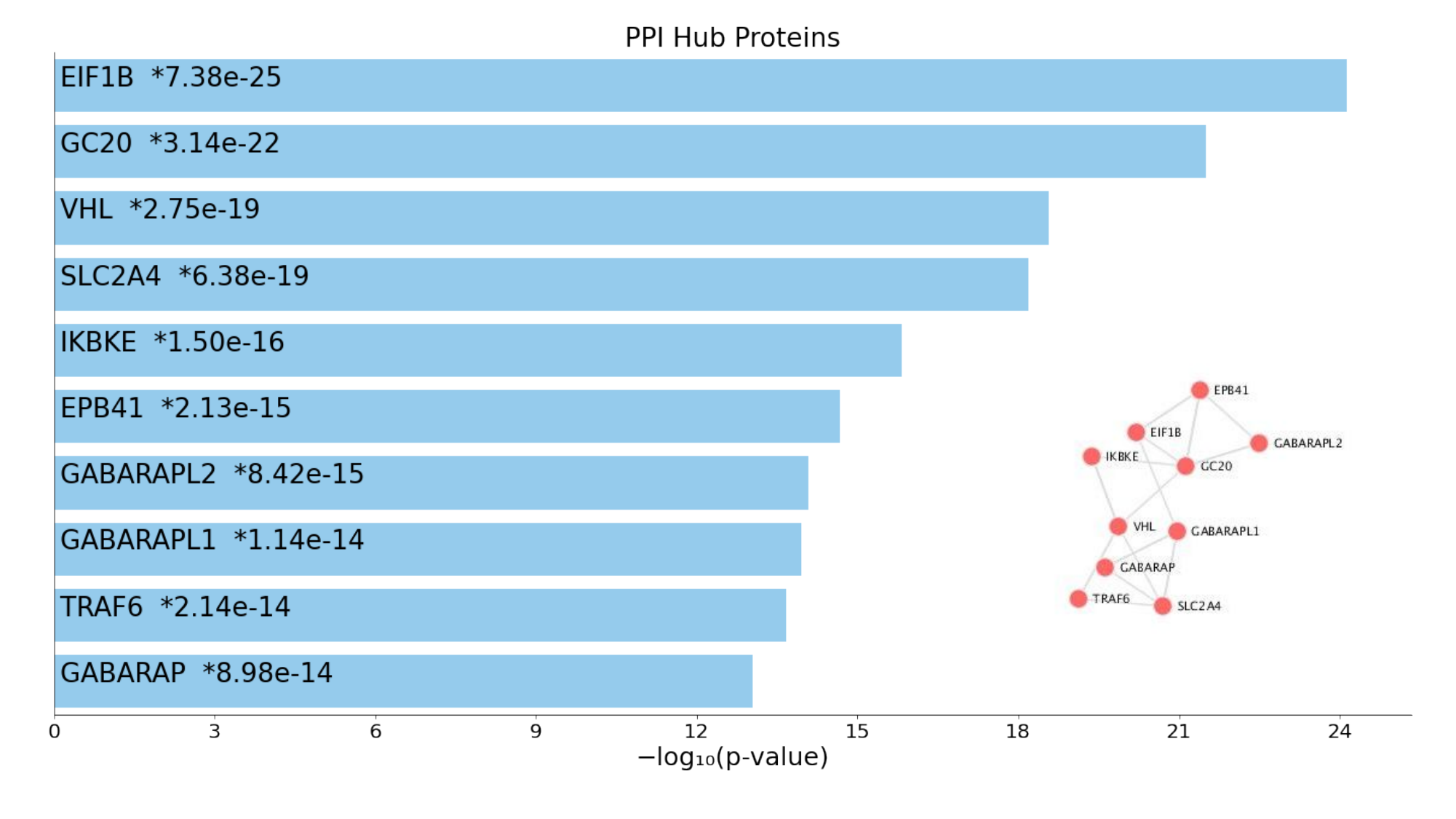

## Slide 17
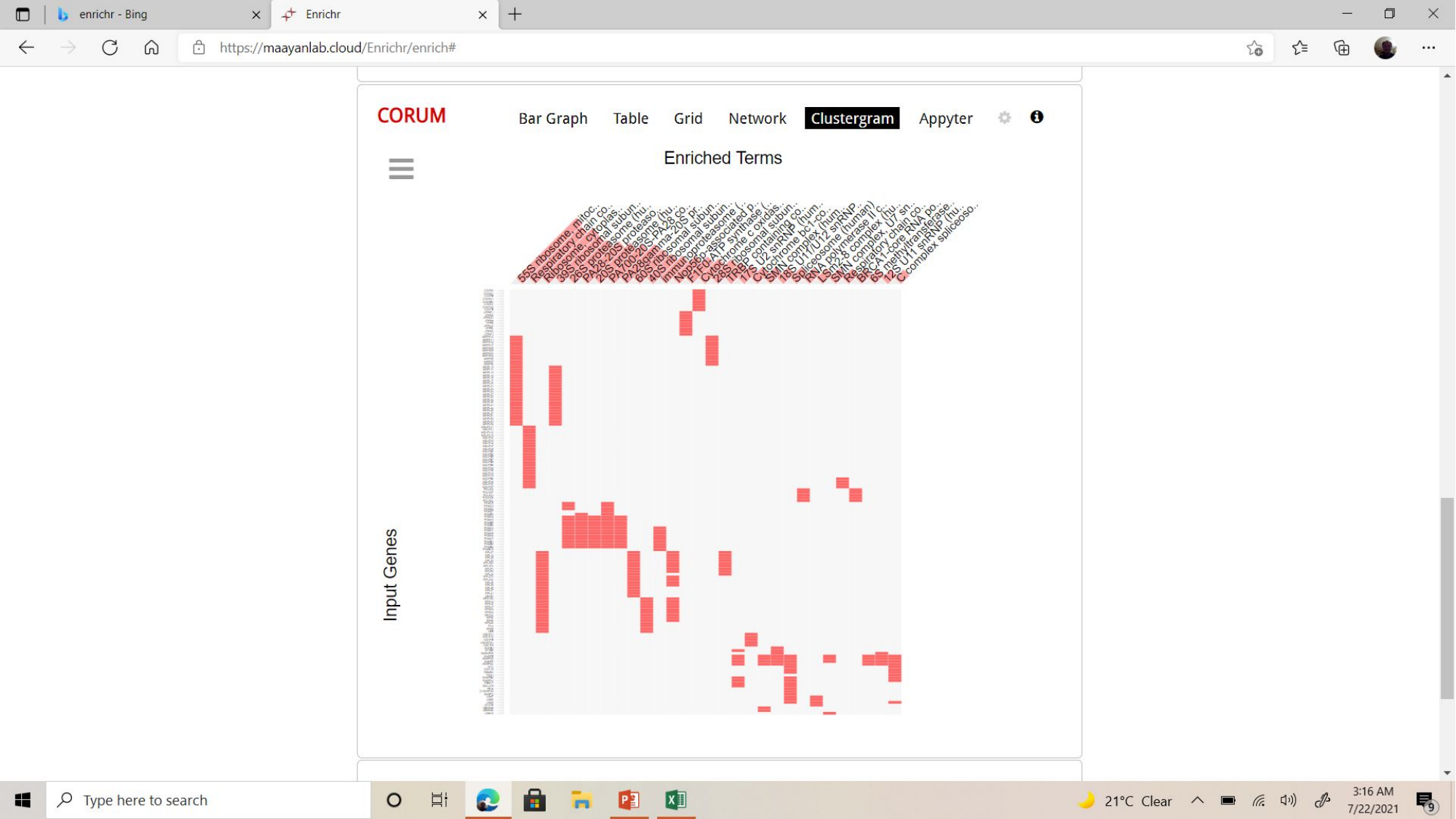

## Slide 18
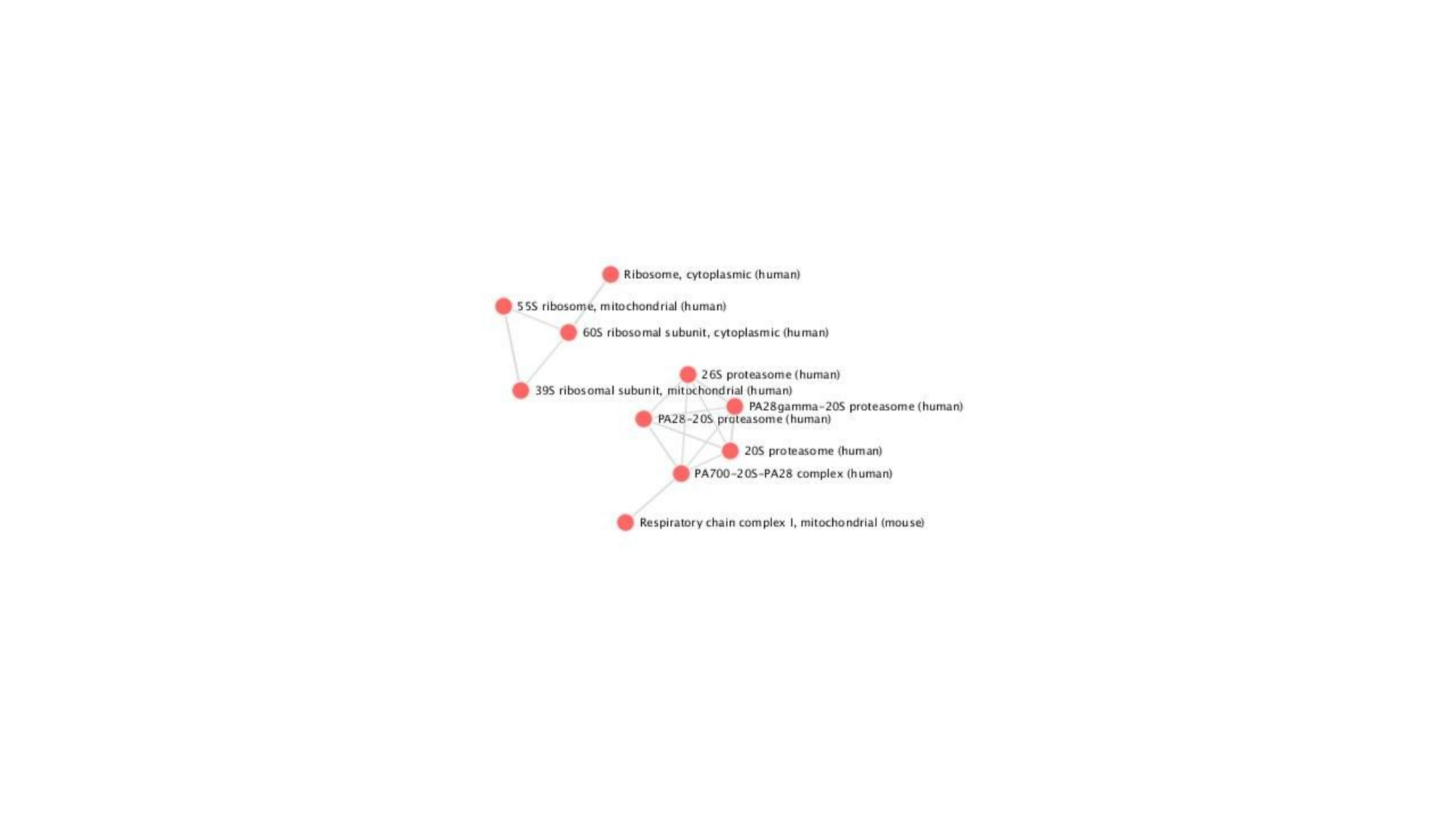

## Slide 19
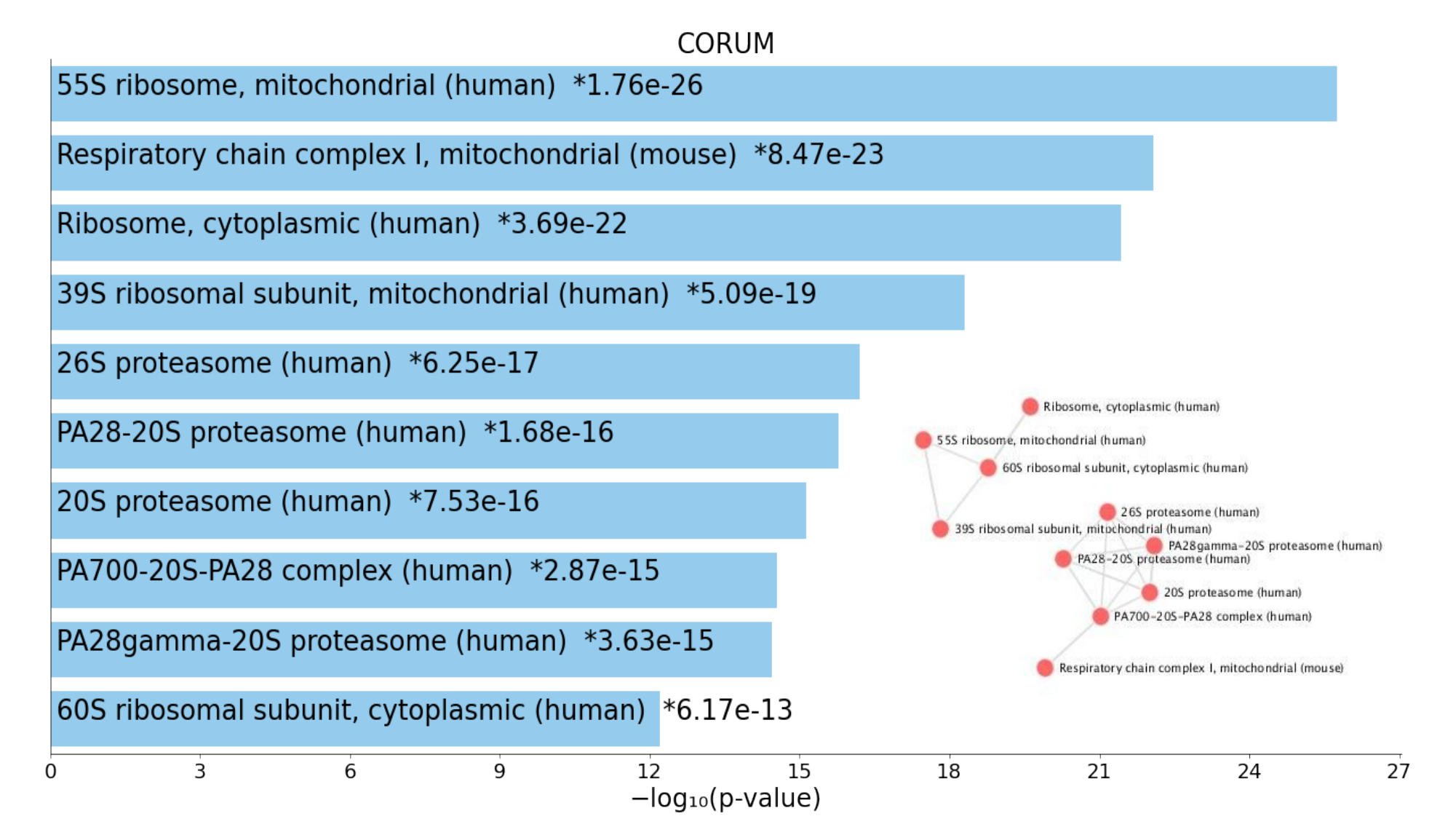

Supplement: vdac180_suppl_Supplementary_Materials [file vdac180_suppl_supplementary_materials.zip › Supplemental Summary SS2. GSEA of 764 fb-PMT up-regulated DEGs.pptx]
