# Supplementary material for: Discovery of novel thyrointegrin αvβ3 antagonist fb-PMT (NP751) in the management of human glioblastoma multiforme
Source: Neurooncol Adv. 2022 Dec 8;5(1):vdac180. doi: 10.1093/noajnl/vdac180 (PMC9985163; doi:10.1093/noajnl/vdac180)
Supplement: vdac180_suppl_Supplementary_Materials [file vdac180_suppl_supplementary_materials.zip › Supplemental Text. GBM_fbPMT Gene expression analysis Methods Results References.docx]

# **Anti-cancer and Anti-angiogenesis activities of fb-PMT in experimental models of growth-factor-induced angiogenesis and human Glioblastoma Multiforme**

Kavitha Godugu^1^, Bruce A Hay^1^, Gennadi V. Glinsky^2^, Shaker A Mousa^1^*

^1^The Pharmaceutical Research Institute, Albany College of Pharmacy and Health Sciences, Rensselaer, NY, USA

^2^Institute of Engineering in Medicine, University of California, San Diego, CA 92093, USA

**Genome-wide gene expression profiling experiments**

**Methods**

**Cell cultures, RNA isolation, and processing for gene expression profiling experiments**

Sub confluent U87-luc human GBM cells were treated with 30 µM of fb-PMT or vehicle (PBS) and incubated for 48 h (n=3). Cells were lysed in TRI reagent solution and gene expression profiling studies were performed at the Center for Functional Genomics at the University at Albany. RNeasy Plus Mini Kit (Qiagen Inc. Valencia, CA) was used to extract RNA from samples. cDNA was prepared using the SMART-Seq v4 Ultra Low Input RNA kit (Takara, Mountain View, CA). Cancer cells were cultured in 50 cm² cell culture flasks with 10 ml phenol red free IMDM supplemented with 10% FBS, 1% penicillin, and 1% streptomycin. During the passaging, cells were cultured at 37°C to sub-confluence and treated with 0.25% (w/v) trypsin/EDTA to induce cell release from culture flasks. Cells were treated (at 50% confluence) with 30µM of the fb-PMT for 48 hours. Cells were lysed in TRI reagent and total RNA were extracted from lysates. Isolated total RNA was checked for quality before being utilized for microarray analysis. The quality and the concentrations of the extracted RNA was analyzed using the NanoDrop (Thermo Fisher Scientific, Waltham, MA) and Agilent Bioanalyzer (Agilent Technologies, Santa Clara, CA). RNA samples (100ng) deemed to be of sufficient quality (RIN greater than 8) were processed according to the standard Affymetrix RNA labeling protocol. Three independent biological replicates of control and treated samples were concurrently interrogated in gene expression profiling analyses. In preliminary experiments, the treatment dose and duration were carefully selected not to significantly affect growth and survival of target cells during the duration of experiments.

**Genome-wide gene expression profiling analysis, data retrieval, processing, and analytical protocols for identification of differentially expressed genes**

Gene expression profiling experiments were performed using 2 analytical platforms: the RNAseq methods and the Affymetrix microarray analytical platform. Samples were processed according to the standard Affymetrix RNA labeling protocol. Labeled RNA samples were processed for hybridization employing the Clariom™ S human array platform (Affymetrix, Santa Clara, CA) at the Center for Functional Genomics, University at Albany, Rensselaer, NY. Briefly, 100 ng of total RNA was processed using the WT Plus Reagent kit (Affymetrix). Sense target cDNAs were generated using the standard Affymetrix WT protocol and hybridized to Affymetrix Human Clariom S arrays. Arrays were washed, stained, and scanned on a GeneChip 3000 7G scanner using Affymetrix GeneChip Command Console Software (AGCC). Transcriptome Analysis Console Software (TAC v3.0.1.5) was used to identify differentially expressed genes (DEGs). Briefly, the CEL files were summarized using the SST-RMA algorithm in TAC and the normalized data were subjected to one-way ANOVA with a Benjamin Hochberg False Discovery Rate correction included (p<0.05). A 1.5-fold expression change cut-off was used to select entities that were statistically differentially expressed between the conditions being compared (treated and untreated groups). In the standard workflow protocol, the fragmented biotin-labeled cDNAs were hybridized for 16 h to Affymetrix Arrays, scanned on an Affymetrix Scanner 3000 7G using AGCC software, and processed as described above. Alternatively, CEL files after QC screening using Affymetrix Expression Console software were imported into GeneSpring GX11.5 (Agilent Technologies). The data was then quantile normalized using PLIER and baseline transformed to the median of the control samples. The probe sets were further filtered to exclude the bottom 20th percentile across all samples. The resulting entity lists were subjected to an unpaired T-test with the Benjamini-Hochberg False Discovery Rate (FDR) correction and a 1.5-fold expression changes filter to identify differentially expressed transcripts between the control and test conditions at a p-value <0.05. During the selection of differentially-expressed genes, both nominal and FDR adjusted p-values were considered. All analyzed and reported data are MIAME compliant and the raw data have been deposited in Gene Expression Omnibus (GEO) as detailed on the Microarray Gene Expression Data Society (MGED) society website (<http://www.mged.org/Workgroups/MIAME/miame.html> ). Overall, the workflow of the microarray analyses was modeled based on previously published contributions [1-3].

Gene set enrichment analyses (GSEA) of differentially expressed genes (DEGs) were carried-out using the Enrichr bioinformatics platform, which enables the interrogation of nearly 200,000 gene sets from more than 100 gene set libraries. The Enrichr API (January 2018 through June 2021 releases) [4-6] was used to test genes of interest for significant enrichment in numerous functional categories. When technically and analytically feasible, different sets of DEGs defined at multiple significance levels of statistical metrics and comprising from dozens to several thousand individual genetic loci were analyzed using differential Gene set enrichment analysis (GSEA) to gain insights into biological effects of DEGs and infer potential mechanisms of anticancer activities. This approach was successfully implemented for identification and characterization of human-specific regulatory networks governed by human-specific transcription factor-binding sites [7-11] and functional enhancer element [12-14], 13,824 genes associated with 59,732 human-specific regulatory sequences [15], 8,405 genes associated with 35,074 human-specific neuroregulatory single-nucleotide changes [16], and 8,384 genes regulated by stem cell-associated regulatory sequences [17]. Initial GSEA entail interrogations of each specific set of DEGs (all statistically significant DEGs; up-regulated DEGs; down-regulated DEGs) using ~30 distinct genomic databases, including comprehensive pathway enrichment Gene Ontology (GO) analyses followed by in-depth analyses of the selected genomic databases deemed most statistically informative. In all reported tables and plots, in addition to the nominal p values and adjusted p values (corrected for multiple hypothesis testing), the statistical metrics designated “combined scores” were calculated by Enrichr software, which represent a product of the significance estimate and the magnitude of enrichment (combined score c = log(p) * z, where p is the Fisher’s exact test p-value and z is the z-score deviation from the expected rank).

**Results**

**Overview of mechanisms of anticancer activities of the fb-PMT using genome-wide expression profiling of human GBM cells**

We carried out genome-wide expression profiling analyses of U87-luc human GBM cells treated with non-toxic doses of the fb-PMT. RNAseq experiments identified 1397 genes expression of which was significantly altered by the fb-PMT (**Supplemental Tables S1 & S2**). There were 633 down-regulated and 764 up-regulated differentially expressed genes (DEGs) identified at the p value < 0.05 adjusted for multiple hypothesis testing (**Supplemental Tables S1 & S2).** At the threshold of nominal p value < 0.05, 3673 DEGs were identified, 2085 and 1588 of which were up- and down-regulated, respectively. To understand potential mechanisms of the fb-PMT anti-cancer activities and gain insights into biological and molecular functions of genes expression of which was significantly affected in GBM cells by the fb-PMT treatment, Gene Set Enrichment Analyses (GSEA) of 764 up-regulated and 633 down-regulated DEGs were carried-out using the Enrichr bioinformatics platform (Methods). These analyses applied to ~ 30 genomics and proteomics databases identified hundreds of significantly enriched records (adjusted p value < 0.05) and associated sub-sets of DEGs significantly affected by fb-PMT treatment of human GBM cells (**Table 1; Supplemental Tables S1 and S2; Supplemental Summaries SS1 and SS2**). Notable examples of significantly affected signal transduction pathways of direct relevance to pathogenesis of GBM include Glioma Stem Cell Program Activation and Secondary Glioblastoma pathways (Elsevier Pathway Collection database) as well as Glioblastoma Signaling Pathway (WikiPathways 2021 Human database). Several signal transduction pathways with established or suspected roles in GBM pathogenesis have been identified by independent GSEA of multiple databases: Notch Signaling Pathway (NCI-Nature 2016; WikiPathways 2021 Human; KEGG 2021 Human database; BioPlanet 2019 database; Reactome 2016 database); Hedgehog Signaling Pathway (NCI-Nature 2016; WikiPathways 2021 Human; KEGG 2021 Human database; BioPlanet 2019 database; Panther 2016 database); NGF Signaling Pathway (Reactome 2016 database; BioPlanet 2019 database); TGF-beta Signaling Pathway (WikiPathways 2021 Human; BioPlanet 2019). Other examples of significantly affected signal transduction pathways of clearly-defined potential biological relevance to regulation of angiogenesis as well as tumor cells’ growth and survival include VEGFA-VEGFR2 Signaling Pathway; EGF/EGFR Signaling Pathway; PI3K-AKT Signaling Pathways; PDGF Signaling Pathway; FGFR1/2/3 Signaling Pathway; Integrin’s Signaling in Angiogenesis; HIF2A & HIF1A Signaling Pathway; VEGFR1 & VEGFR2 Signaling Pathway; Pathways Regulating Hippo Signaling; Nuclear Receptors Meta Pathway and Androgen Signaling Pathway; Thyroid Hormone Signaling Pathways and WNT Signaling Pathway; YAP1- and WWTR1 (TAZ) Pathway. A graphical summary of these observations is presented in the **Figure S1** and extended comprehensive documentations of these findings, including corresponding lists of fb-PMT down-regulated DEGs, are reported in the **Supplemental Tables S1 and S2; Supplemental Summaries SS1 and SS2.**

We observed apparently distinct quantitative patterns of significantly enriched records for signal transduction pathways associated with up-regulated and down-regulated DEGs (**Table 1; Supplemental Tables S1 and S2; Supplemental Summaries SS1 and SS2**). Compared with up-regulated genes, genes down-regulated following the fb-PMT treatment manifest larger numbers of significant associations among genes whose protein products are engaged in protein-protein interactions (PPI) with transcription factors (reported in the TFs PPI database) and PPI Hub Proteins (proteins known to interact with at least 50 other proteins to form biologically-active multi-protein complexes reported in the PPI Hub Proteins database). Genes expression of which is up-regulated following the fb-PMT treatment manifest markedly larger number of significant enrichment records among genes whose expression is altered following exposure of cells to various endogenous ligands such as growth factors, cytokines, interleukins, hormones, etc. (reported in the Ligand Perturbations from GEO databases). Noteworthy significantly enriched records of fb-PMT treatment-affected signal transduction pathways triggered by exposures to endogenous ligands include signaling pathways induced by FGF2; Estradiol; TGF-beta; Neuromedin U; Growth Hormone; Thyroid Hormone; IGF-1; and multiple interleukins, including IL-1-beta; IL-2; IL-10; IL-15; IL-33; IL-6; IL-12 (**Supplemental Table S1; Supplemental Summary SS2**). Notably, GSEA of the DisGeNET database reporting genes implicated in pathogenesis of several thousand human disorders revealed that genes expression of which is down-regulated after fb-PMT treatment manifest significant enrichment among genes identified in 502 various disease records, while fb-PMT up-regulated genes are significantly enriched among genes implicated in 59 disease records (**Tables 1;** **Supplemental Tables S1 and S2**).

**Integrative and case-specific analyses of putative molecular mechanisms of anticancer activity of the fb-PMT inferred from gene expression profiling experiments**

We carried out additional analyses to explore the potential molecular mechanisms of the apparently global effects of the fb-PMT on signal transduction pathways in GBM cells. To this end, we focused our analytical effort on two aspects of the fb-PMT actions: 1) integrative analysis of effects on expression of genes whose protein products are engaged in protein-protein interactions (PPI) with multiple transcription factors (TFs PPI) and/or with intracellular proteins known to form the biologically-active multiprotein complexes via PPI with at least 50 other proteins (**Figure 10**); and 2) case-specific analysis of effects on expression of cancer driver genes (**Figure 11**).

Integrative analyses of fb-PMT down-regulated DEGs whose protein products are engaged in PPI with multiple transcription factors (TFs PPI) and/or with PPI Hub Proteins reveal the auto-regulatory PPI networks of TFs PPI and PPI Hub proteins that a) regulate expression of DEGs down-regulated by fb-PMT treatment in human GBM cells; and b) fb-PMT down-regulate expression of genes encoding the TFs PPI and PPI Hub Proteins constituting members of these PPI networks (**Figure 10; Supplemental Tables S3 and S4**). For example, a set of 62 TFs PPI was identified from 99 TFs interacting via PPI with 633 fb-PMT down-regulated targets (**Table 1; Supplemental Table S3**) that regulate expression of fb-PMT target genes (these 62 TFs PPI were designated up-stream regulatory TFs PPI). GSEA of 62 up-stream regulatory TFs PPI revealed their interactions in human cells with 196 TFs PPI and 121 PPI Hub Proteins (**Figure 10A**). fb-PMT treatment down-regulated expression of 13/99 TFs PPI (**Table 1**; last column), as well as 7/62 up-stream regulatory TFs PPI; 25/196 TFs PPI; 22/121 PPI Hub Proteins. Similarly, a set of 14 PPI Hub Proteins was selected from 84 PPI Hubs interacting via PPI with 633 fb-PMT down-regulated targets (**Table 1; Supplemental Table S4**) that regulate expression of fb-PMT target genes (these 14 PPI Hub Proteins are designated up-stream regulatory PPI Hub Proteins). They interact in human cells via PPI with 184 TFs PPI and 146 PPI Hub Proteins (**Figure 10B; Supplemental Table S4**). fb-PMT treatment down-regulated expression of 21/84 PPI Hub Proteins, as well as 2/14 up-stream regulatory PPI Hubs; 23/184 TFs PPI; 29/146 PPI Hub Proteins.

Cancer driver genes, when mutated, promote cancer initiation, development, and progression toward metastatic disease. We observed that expression of 26 cancer driver genes is downregulated by fb-PMT (**Figure 11**), representative examples of which include EGFR, NOTCH1/2, MAPK1, CCND1, and BRAF. Among six up-regulated cancer driver genes, four genes encode proteins known to function as negative regulator of cell proliferation (CDKN2A); inducer of apoptosis (BAX); tumor suppressor (RPL22); inhibitor of transactivation of insulin promoter by recruiting a repressor complex (SPOP). One of the fb-PMT up-regulated cancer driver genes (RET) encodes a dependence receptor, which promotes cell’s survival following interactions with its ligand, GDNF. However, when then GDNF level is diminished, RET triggers apoptosis in target cells. Notably, fb-PMT treatment significantly down-regulate the GDNF expression (**Figure 11**), suggesting that fb-PMT treatment may activate RET dependence receptor-triggered programmed cell death of GBM cells. Consistent with this hypothesis, we observed that multiple genes engaged in signaling events regulated by RET tyrosine kinase in human cells (ID: e4431190-6195-11e5-8ac5-06603eb7f303) are significantly down-regulated by fb-PMT (*IRS1; GRB10; MAPK1; PRKCA*). We experimentally validated this mode of actions of the fb-PMT by demonstrating that anti-cancer effects of sub-optimal 50% inhibitory doses of fb-PMT on human GBM cells could be rescued by GDNF in a dose-dependent manner (**Figure 12B** and **12E**). Conversely, growth-promoting and pro-survival effects of GDNF on human GBM cells could be markedly inhibited by the fb-PMT in a dose-dependent manner (**Figures 12C** and **12F**).

Collectively, these observations support the idea that b-PMT effects on *RET* and *GDNF* expression could be sufficient to induce the RET-dependent programmed cell death of glioblastoma cells. fb-PMT markedly increases *RET* expression and significantly inhibits *GDNF* expression, thus removing the survival ligand for dependence receptor RET and triggering the apoptosis of GBM cells. Known RET functions and mechanisms of actions further support the hypothesis that fb-PMT anti-cancer activity may be responsible, in part, by effects on RET signaling pathway. RET regulates both cell death/survival balance and positional information and modulates cell adhesion via its cleavage by caspase in sympathetic neurons as well as mediates cell migration in an integrin (e.g. ITGB1 and ITGB3)-dependent manner. RET is biologically active in the absence of ligand, triggering apoptosis. Thus, RET acts as a classical dependence receptor. In the presence of the ligand GDNF, RET promotes survival and down regulates growth hormone production. In the absence of GDNF, RET triggers cell death via apoptosis.

**Conclusion**

Overall, gene expression profiling experiments indicate that there are two principal biologically meaningful mechanisms explaining how drugs may cause the potent anticancer effects: 1) molecular interference with multiple signaling pathways specified in the reports; 2) molecular mimicry of the effects of specific genes, in particular, genes with known functions as tumor suppressors, defined by gene silencing and/or gene overexpression experiments. It is important to note that fb-PMT exhibited significant inhibitory effects on the Hedgehog pathway, which are very important to overcome the TMZ resistance in GBM patients.

RNA-seq analyses of the fb-PMT effects on gene expression in GBM cells demonstrated consistent evidence of molecular interferences with gene expression pathways implicated in tumor progression, cancer cells’ growth and survival, and tumor neovascularization. Among the genes whose expression is downregulated in tumor cells following fb-PMT treatment, are MAPK1, AKT, Hedgehog, NOTCH1 and Wnt, all of which encode products involved in intracellular signaling. The respective gene products relate to cell division and cell migration. Examples of upregulated by fb-PMT genes include BAD, BID and BAX, the protein products of which are pro-apoptotic. The examples of genomic actions of fb-PMT of potential relevance for inhibition of neovascularization include decreased expression of FGFR1, EGFR and components of the downstream signaling pathways for VEGFA, VEGFR2 and PDGF.

**Table 1.** Differential GSEA of distinct expression signatures of the fb-PMT target genes in human GBM cells.

| Database | 764 fb-PMT genes | 633 fb-PMT genes | 62 TF PPI genes | 15 PPI Hub Proteins | 13 TF PPI genes |
| --- | --- | --- | --- | --- | --- |
| Transcription Factor PPIs | 10 | **99** | 196 | 179 | 169 |
| ARCHS4 TFs Co-expression in Human Tissues | 148 | **354** | 53 | 17 | 124 |
| Enrichr Submissions TF-Gene Co-occurrence | 135 | **1197** | 1520 | 1321 | 1082 |
| TF Perturbations Followed by Expression | **951** | 302 | 72 | 27 | 36 |
| KEGG 2021 Human | 21 | 16 | 84 | 106 | 41 |
| PPI Hub Proteins | 33 | **84** | 121 | 157 | 71 |
| BioPlanet 2019 | 89 | 104 | 291 | 358 | 183 |
| DisGeNET | 59 | **502** | 1961 | 2460 | 925 |
| Jensen Disease database | 3 | 17 | 34 | 97 | 56 |
| Jensen Cell’s Compartments | 163 | 94 | 160 | 166 | 99 |
| WikiPathways 2021 Human | 13 | 36 | 213 | 262 | 109 |
| WikiPathways 2019 Mouse | 5 | 15 | 55 | 59 | 32 |
| Panther 2016 | 1 | 2 | 19 | 22 | 10 |
| NCI-Nature 2016 | 0 | 37 | 79 | 100 | 51 |
| MSigDB Hallmark 2020 | 6 | 3 | 19 | 12 | 5 |
| Reactome 2016 | 353 | 121 | 109 | 150 | 147 |
| GO Biological Process 2021 | 157 | 76 | 371 | 453 | 379 |
| GO Molecular Function 2021 | 16 | 5 | 65 | 55 | 44 |
| GO Cellular Component 2021 | 50 | 12 | 3 | 5 | 4 |
| MSigDB Oncogenic Signatures | 1 | 0 | 11 | 2 | 0 |
| BioCarta 2016 | 0 | 12 | 73 | 93 | 42 |
| Elsevier Pathway Collection | 0 | 177 | 683 | 840 | 338 |
| Ligand Perturbations from GEO (down) | **159** | 6 | 7 | 6 | 5 |
| Ligand Perturbations from GEO (up) | **149** | 13 | 37 | 19 | 3 |
| Gene Perturbations from GEO (down) | **1131** | 35 | 29 | 4 | 5 |
| Gene Perturbations from GEO (up) | **1168** | 64 | 30 | 5 | 2 |
| MGI Mammalian Phenotype Level 4 2021 | 0 | **281** | 516 | 585 | 222 |
| Human Phenotype Ontology | 46 | **206** | 53 | 178 | 90 |
| LINCS L1000 Ligand Perturbations (up) | 0 | 2 | 41 | 38 | 22 |
| LINCS L1000 Ligand Perturbations (down) | 1 | 0 | 4 | 3 | 3 |

Legend: Reported values represent numbers of significantly enriched records (adjusted p-value < 0.05) identified by GSEA in corresponding classification categories; PPIs, protein-protein interactions; TFs, transcription factors. Detailed reports of the GSEA can be found in the Supplemental Tables S1-S4.

**Gene expression profiling analysis section references**

1. Glinsky, G.V., et al., Gene expression profiling predicts clinical outcome of prostate cancer. J Clin Invest, 2004. 113(6): 913-23.

2. Glinsky, G.V., T. Higashiyama, and A.B. Glinskii, Classification of human breast cancer using gene expression profiling as a component of the survival predictor algorithm. Clin Cancer Res, 2004. 10(7): 2272-83.

3. Glinsky, G.V., O. Berezovska, and A.B. Glinskii, Microarray analysis identifies a death-from-cancer signature predicting therapy failure in patients with multiple types of cancer. J Clin Invest, 2005. 115(6): 1503-21.

4. Chen, E.Y., et al., Enrichr: interactive and collaborative HTML5 gene list enrichment analysis tool. BMC Bioinformatics, 2013. 14: 128.

5. Kuleshov, M.V., et al., Enrichr: a comprehensive gene set enrichment analysis web server 2016 update. Nucleic Acids Res, 2016. 44(W1): W90-7.

6. Xie Z, Bailey A, Kuleshov MV, Clarke DJB., Evangelista JE, Jenkins SL, Lachmann A, Wojciechowicz ML, Kropiwnicki E, Jagodnik KM, Jeon M, & Ma’ayan A. Gene set knowledge discovery with Enrichr. Current Protocols, 1, e90. 2021. doi: 10.1002/cpz1.90

7. Glinsky, G.V., Transposable Elements and DNA Methylation Create in Embryonic Stem Cells Human-Specific Regulatory Sequences Associated with Distal Enhancers and Noncoding RNAs. Genome Biology and Evolution, 2015. 7(6): 1432-1454.

8. Glinsky, G.V., Mechanistically Distinct Pathways of Divergent Regulatory DNA Creation Contribute to Evolution of Human-Specific Genomic Regulatory Networks Driving Phenotypic Divergence of Homo sapiens. Genome biology and evolution, 2016. 8(9): 2774-2788.

9. Glinsky, G.V., Activation of endogenous human stem cell-associated retroviruses (SCARs) and therapy-resistant phenotypes of malignant tumors. Cancer Lett, 2016. 376(2): 347-59.

10. Glinsky, G.V., Human-specific genomic features of pluripotency regulatory networks link NANOG with fetal and adult brain development. bioRxiv, 2017: 022913.

11. Glinsky, G.V., Single cell genomics reveals activation signatures of endogenous SCAR's networks in aneuploid human embryos and clinically intractable malignant tumors. Cancer Lett, 2016. 381(1): 176-93.

12. Glinsky, G., et al., Single cell expression analysis of primate-specific retroviruses-derived HPAT lincRNAs in viable human blastocysts identifies embryonic cells co-expressing genetic markers of multiple lineages. Heliyon, 2018. 4(6): e00667.

13. Glinsky, G.V., Contribution of transposable elements and distal enhancers to evolution of human-specific features of interphase chromatin architecture in embryonic stem cells. Chromosome Res, 2018. 26(1-2): 61-84.

14. Glinsky, G. and T.S. Barakat, The evolution of Great Apes has shaped the functional enhancers' landscape in human embryonic stem cells. Stem Cell Res, 2019. 37: 101456.

15. Glinsky, G.V., A Catalogue of 59,732 Human-Specific Regulatory Sequences Reveals Unique-to-Human Regulatory Patterns Associated with Virus-Interacting Proteins, Pluripotency, and Brain Development. DNA Cell Biol, 2020. 39(1): p. 126-143.

16. Glinsky, G.V., Impacts of genomic networks governed by human-specific regulatory sequences and genetic loci harboring fixed human-specific neuro-regulatory single nucleotide mutations on phenotypic traits of modern humans. Chromosome Res, 2020. 28(3-4): 331-353.

17. Glinsky, G.V., Genomics-Guided Drawing of Molecular and Pathophysiological Components of Malignant Regulatory Signatures Reveals a Pivotal Role in Human Diseases of Stem Cell-Associated Retroviral Sequences and Functionally-Active hESC Enhancers. Frontiers in Oncology, 2021. 11: 974. <https://doi.org/10.3389/fonc.2021.638363>
