# Supplementary material for: Discovery of novel thyrointegrin αvβ3 antagonist fb-PMT (NP751) in the management of human glioblastoma multiforme
Source: Neurooncol Adv. 2022 Dec 8;5(1):vdac180. doi: 10.1093/noajnl/vdac180 (PMC9985163; doi:10.1093/noajnl/vdac180)
Supplement: vdac180_suppl_Supplementary_Materials [file vdac180_suppl_supplementary_materials.zip › Supplemntal Figure S1. fbPMT GBM Summary Updated.pptx]

## Slide 1
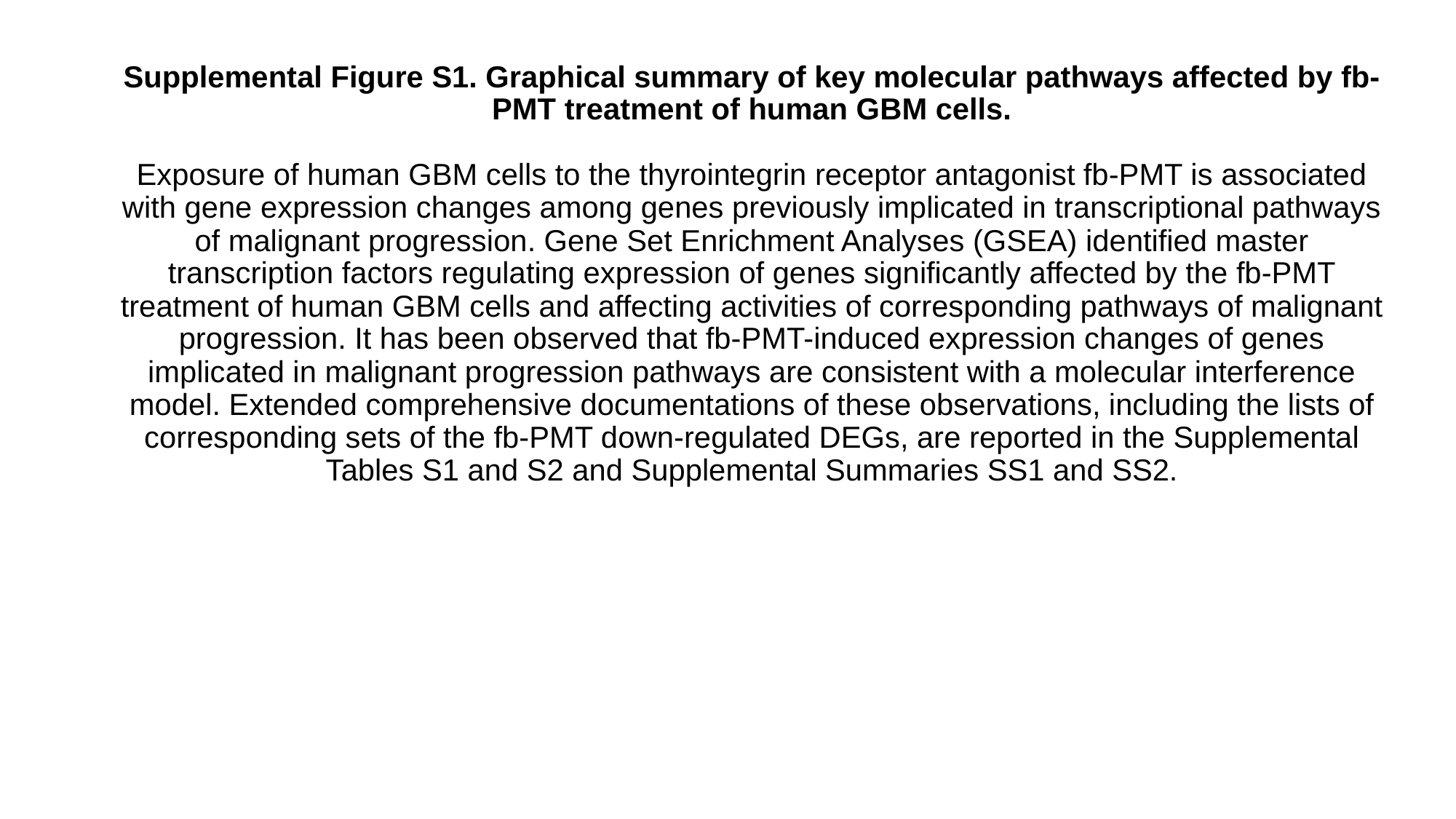

# Supplemental Figure S1. Graphical summary of key molecular pathways affected by fb-PMT treatment of human GBM cells. Exposure of human GBM cells to the thyrointegrin receptor antagonist fb-PMT is associated with gene expression changes among genes previously implicated in transcriptional pathways of malignant progression. Gene Set Enrichment Analyses (GSEA) identified master transcription factors regulating expression of genes significantly affected by the fb-PMT treatment of human GBM cells and affecting activities of corresponding pathways of malignant progression. It has been observed that fb-PMT-induced expression changes of genes implicated in malignant progression pathways are consistent with a molecular interference model. Extended comprehensive documentations of these observations, including the lists of corresponding sets of the fb-PMT down-regulated DEGs, are reported in the Supplemental Tables S1 and S2 and Supplemental Summaries SS1 and SS2.

## Slide 2
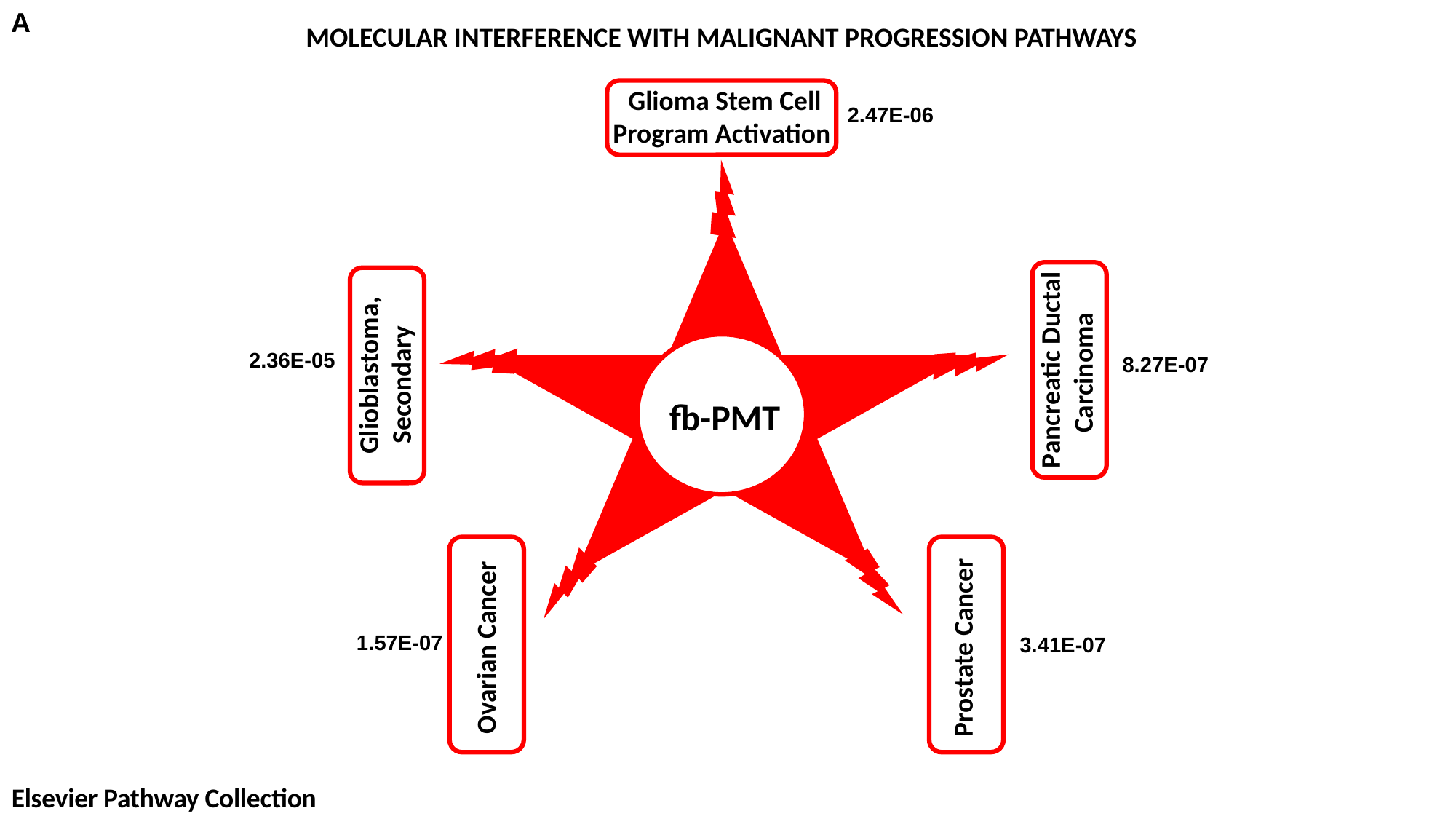

A
MOLECULAR INTERFERENCE WITH MALIGNANT PROGRESSION PATHWAYS
Glioma Stem Cell Program Activation
2.47E-06
Pancreatic Ductal Carcinoma
Glioblastoma, Secondary
2.36E-05
8.27E-07
fb-PMT
Ovarian Cancer
Prostate Cancer
1.57E-07
3.41E-07
Elsevier Pathway Collection

## Slide 3
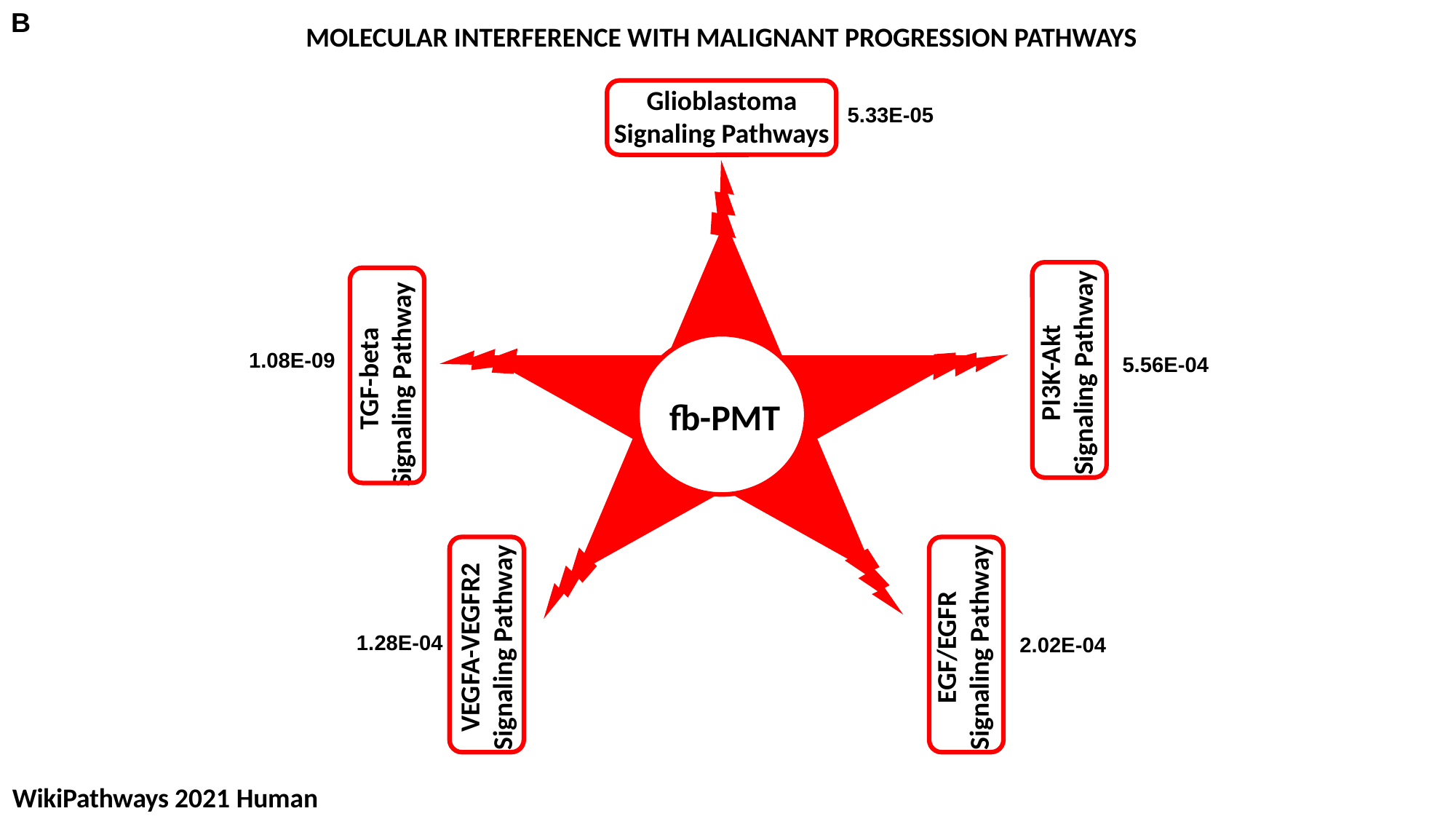

B
MOLECULAR INTERFERENCE WITH MALIGNANT PROGRESSION PATHWAYS
Glioblastoma
Signaling Pathways
5.33E-05
PI3K-Akt
Signaling Pathway
TGF-beta
Signaling Pathway
1.08E-09
5.56E-04
fb-PMT
VEGFA-VEGFR2
Signaling Pathway
EGF/EGFR
Signaling Pathway
1.28E-04
2.02E-04
WikiPathways 2021 Human

## Slide 4
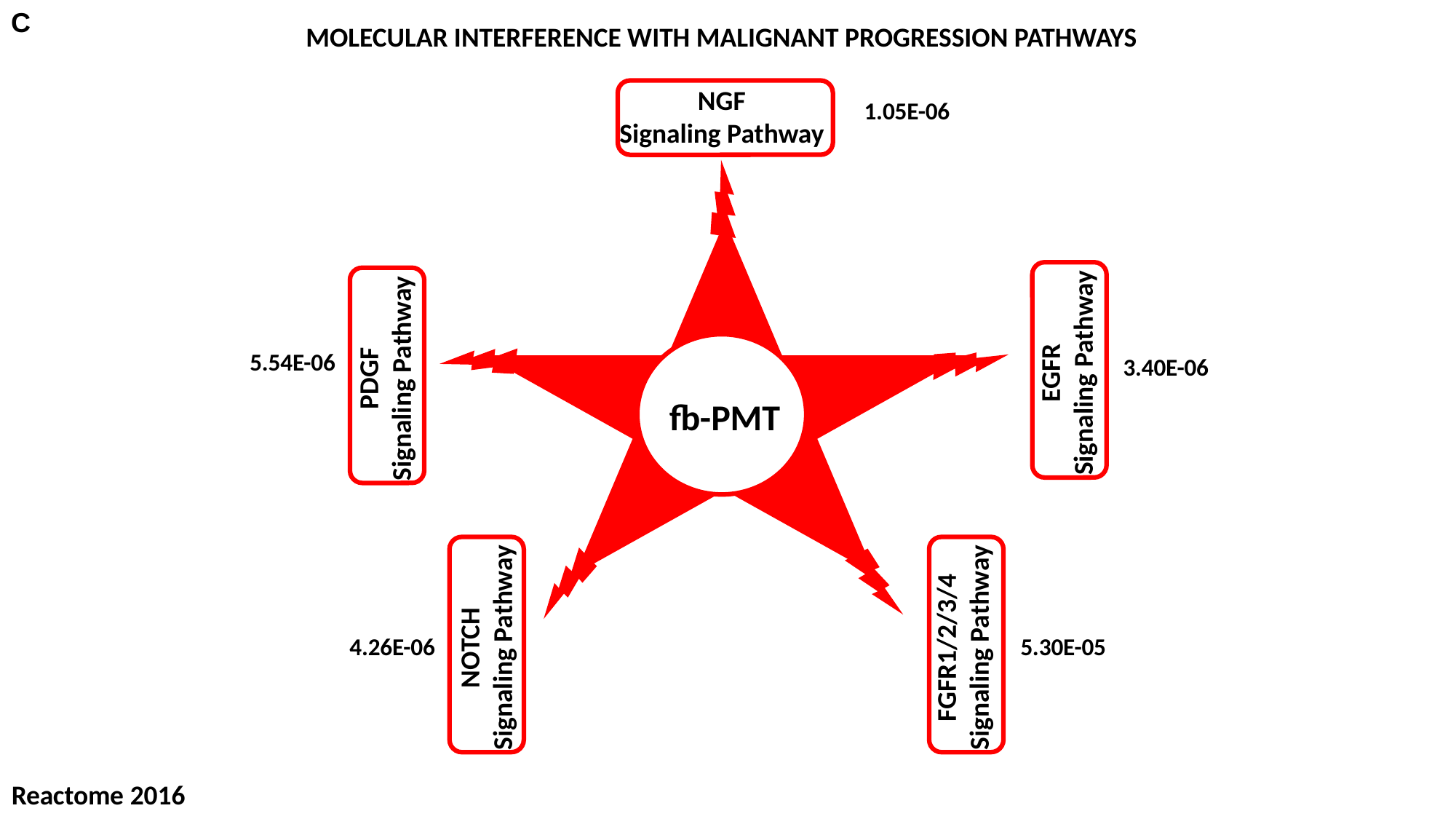

C
MOLECULAR INTERFERENCE WITH MALIGNANT PROGRESSION PATHWAYS
NGF
Signaling Pathway
1.05E-06
EGFR
Signaling Pathway
PDGF
Signaling Pathway
5.54E-06
3.40E-06
fb-PMT
NOTCH
Signaling Pathway
FGFR1/2/3/4
Signaling Pathway
4.26E-06
5.30E-05
Reactome 2016

## Slide 5
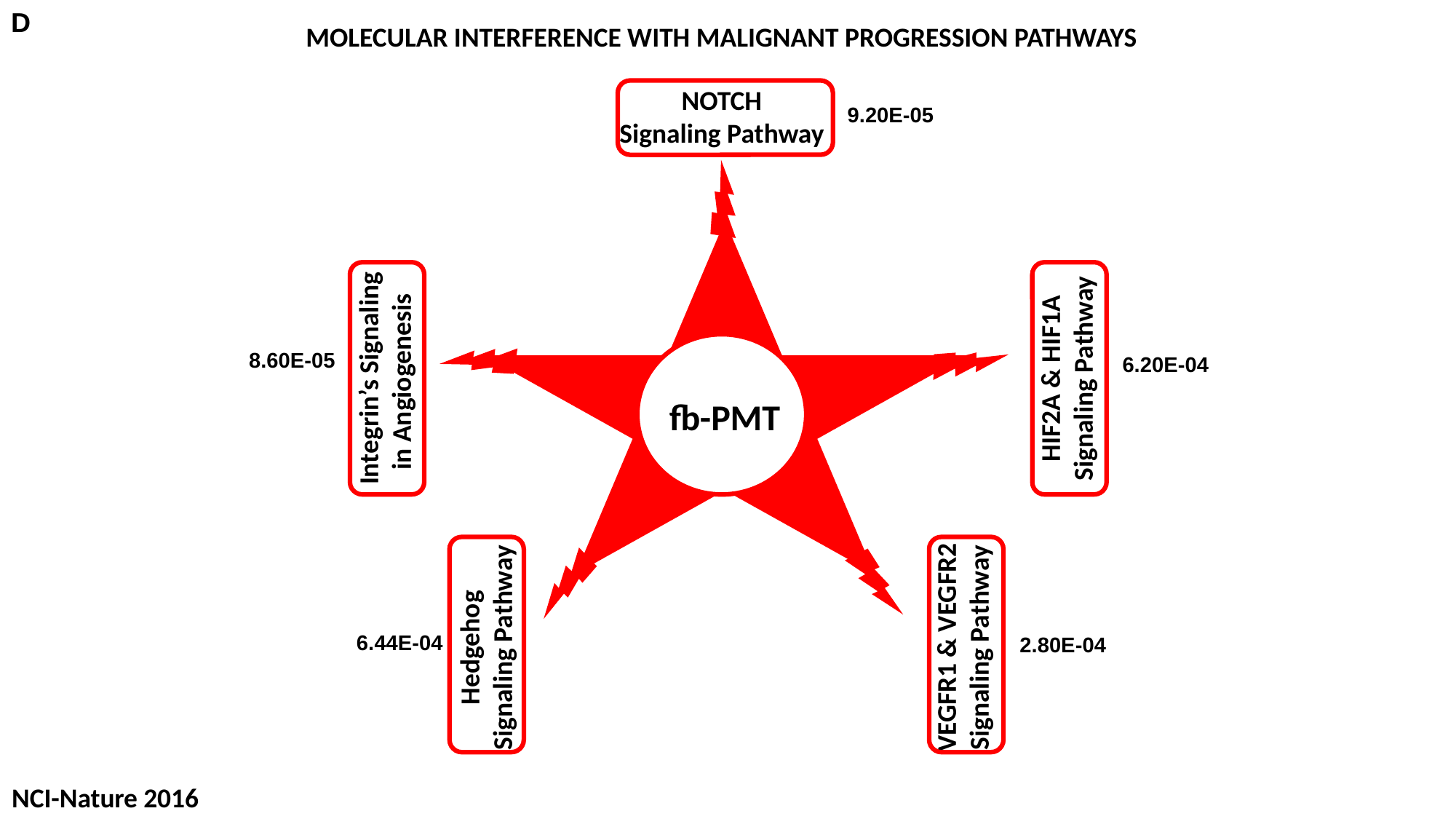

D
MOLECULAR INTERFERENCE WITH MALIGNANT PROGRESSION PATHWAYS
NOTCH
Signaling Pathway
9.20E-05
Integrin’s Signaling in Angiogenesis
HIF2A & HIF1A
Signaling Pathway
8.60E-05
6.20E-04
fb-PMT
Hedgehog
Signaling Pathway
VEGFR1 & VEGFR2
Signaling Pathway
6.44E-04
2.80E-04
NCI-Nature 2016

## Slide 6
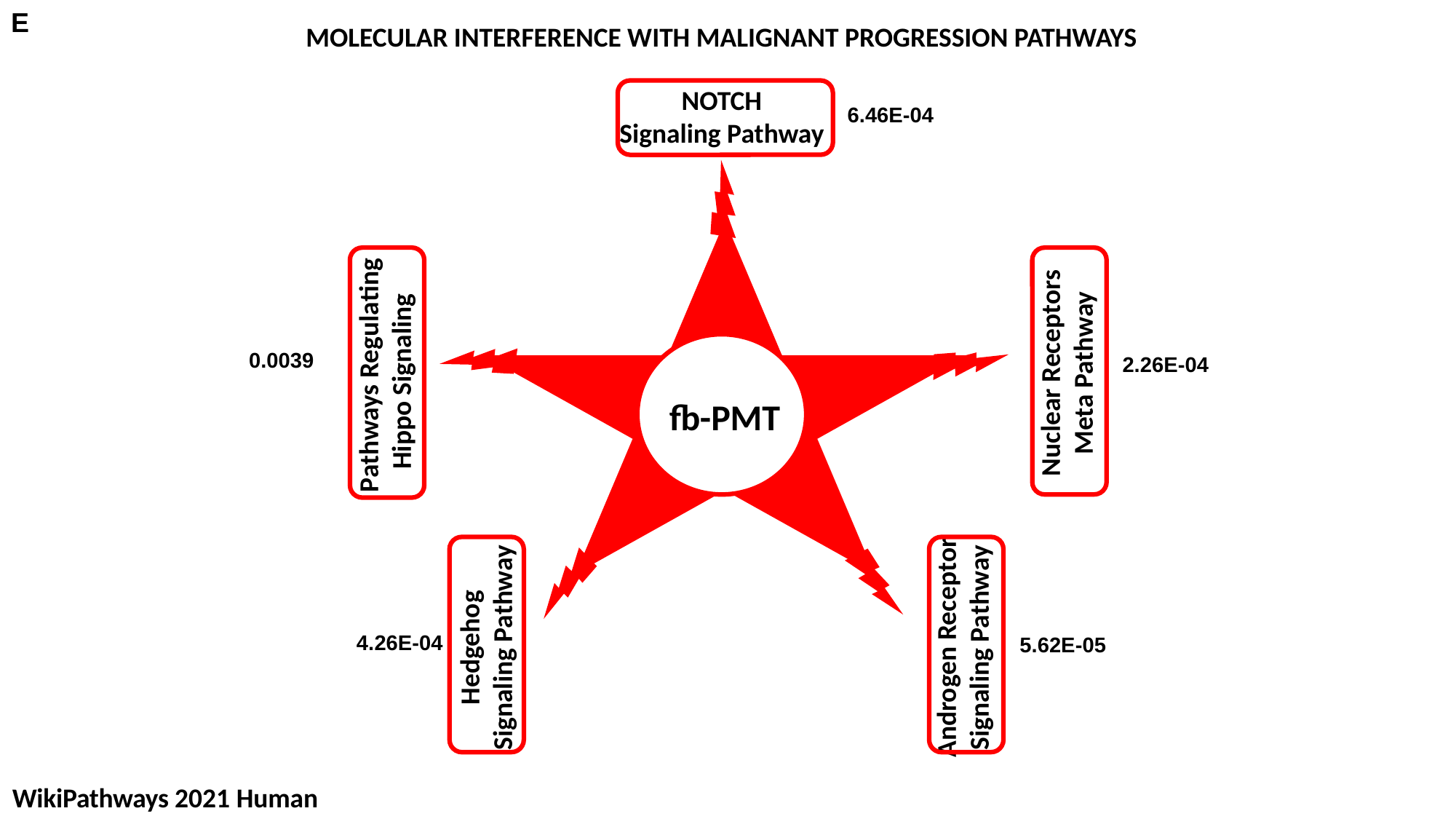

E
MOLECULAR INTERFERENCE WITH MALIGNANT PROGRESSION PATHWAYS
NOTCH
Signaling Pathway
6.46E-04
Nuclear Receptors
Meta Pathway
Pathways Regulating Hippo Signaling
0.0039
2.26E-04
fb-PMT
Hedgehog
Signaling Pathway
Androgen Receptor
Signaling Pathway
4.26E-04
5.62E-05
WikiPathways 2021 Human

## Slide 7
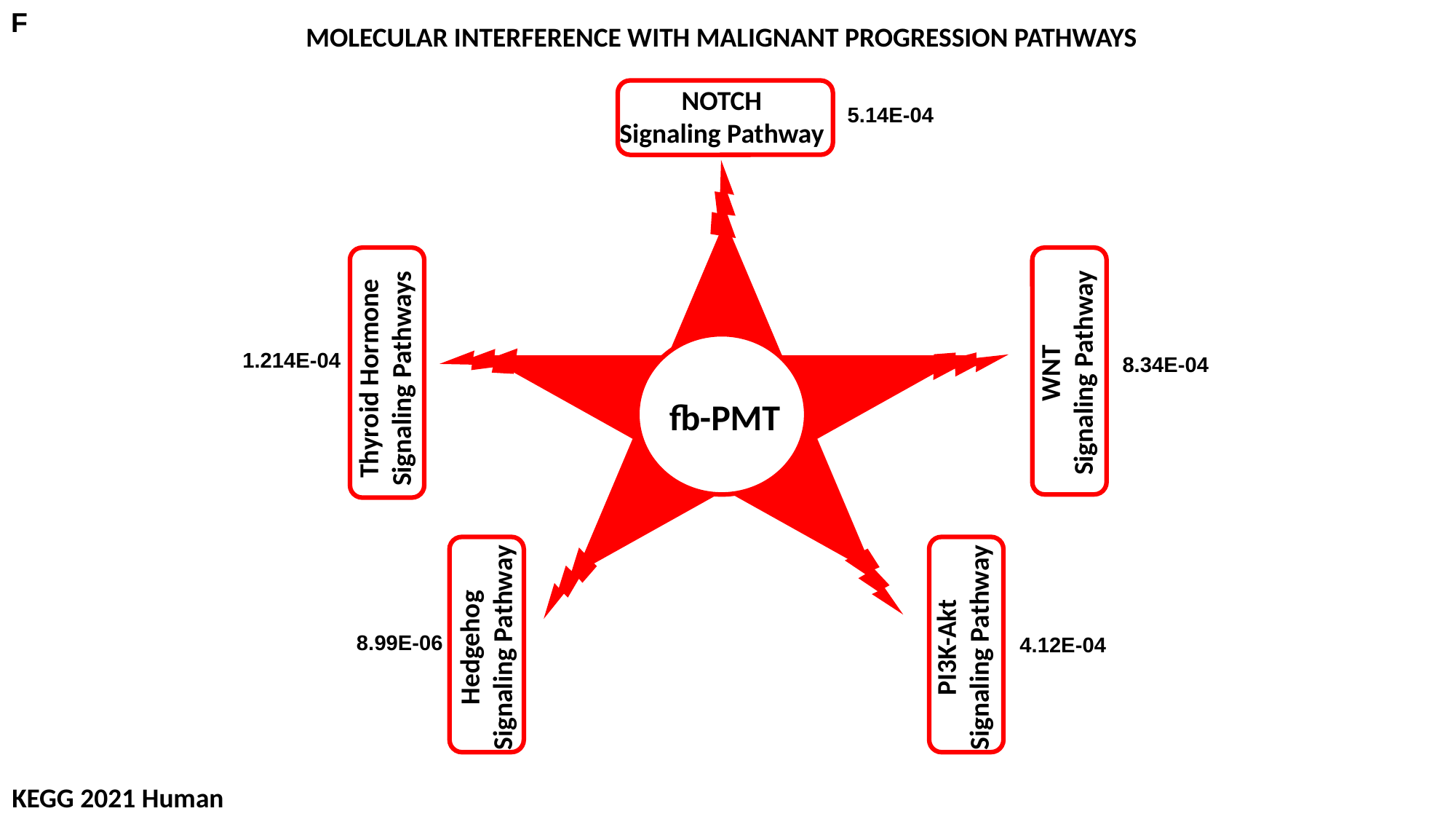

F
MOLECULAR INTERFERENCE WITH MALIGNANT PROGRESSION PATHWAYS
NOTCH
Signaling Pathway
5.14E-04
WNT
Signaling Pathway
Thyroid Hormone Signaling Pathways
1.214E-04
8.34E-04
fb-PMT
Hedgehog
Signaling Pathway
PI3K-Akt
Signaling Pathway
8.99E-06
4.12E-04
KEGG 2021 Human

## Slide 8
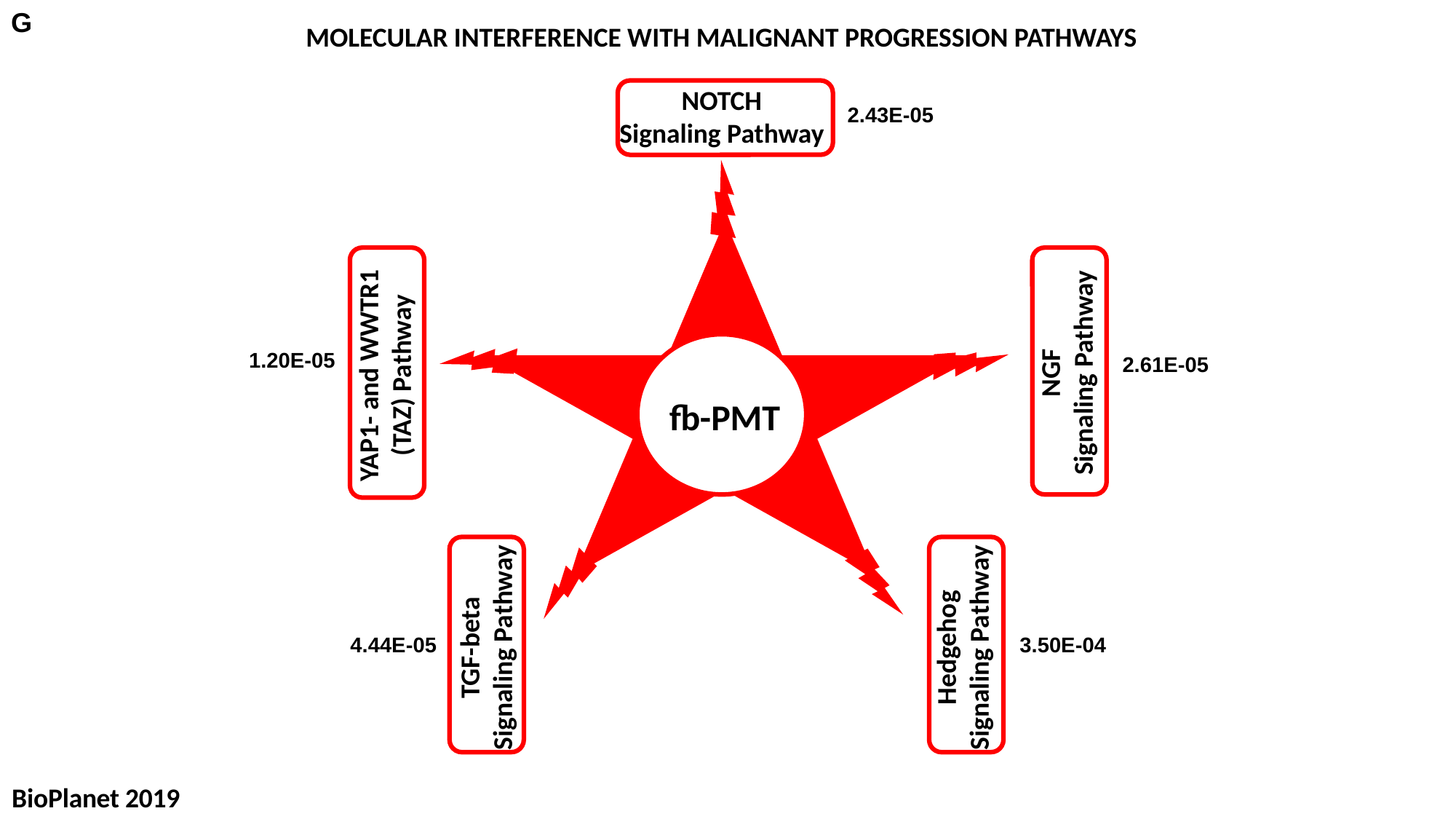

G
MOLECULAR INTERFERENCE WITH MALIGNANT PROGRESSION PATHWAYS
NOTCH
Signaling Pathway
2.43E-05
NGF
Signaling Pathway
YAP1- and WWTR1 (TAZ) Pathway
1.20E-05
2.61E-05
fb-PMT
TGF-beta
Signaling Pathway
Hedgehog
Signaling Pathway
4.44E-05
3.50E-04
BioPlanet 2019

## Slide 9
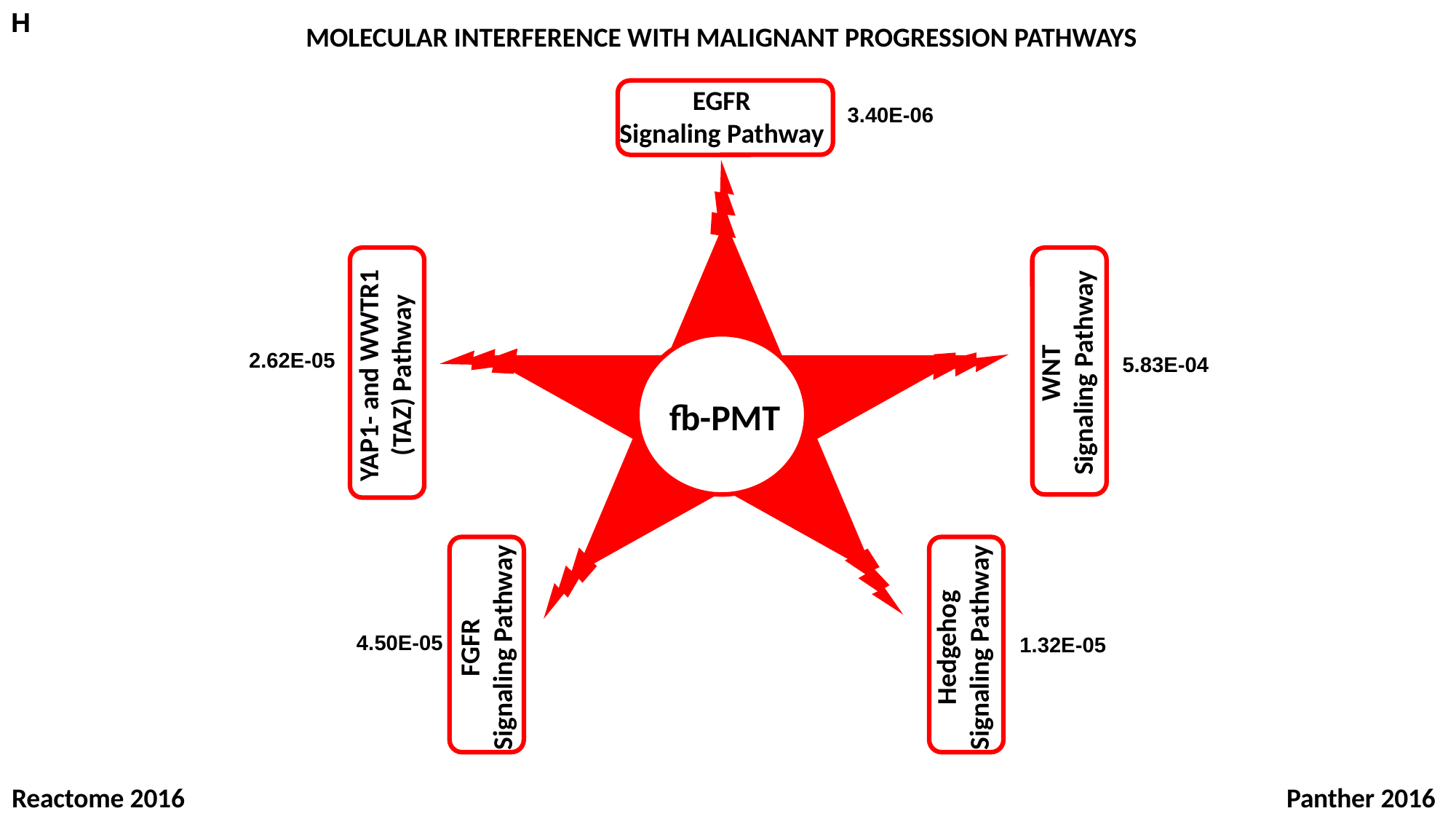

H
MOLECULAR INTERFERENCE WITH MALIGNANT PROGRESSION PATHWAYS
EGFR
Signaling Pathway
3.40E-06
WNT
Signaling Pathway
YAP1- and WWTR1 (TAZ) Pathway
2.62E-05
5.83E-04
fb-PMT
FGFR
Signaling Pathway
Hedgehog
Signaling Pathway
4.50E-05
1.32E-05
Reactome 2016
Panther 2016
